# Cognitive Behavioral Therapy for Individuals With Low Literacy and Perinatal Depression: A Randomized Clinical Trial

**DOI:** 10.1001/jamanetworkopen.2026.11101

**Published:** 2026-05-07

**Authors:** Eliza Kleban, Ashleen Lee, Aminata Koroma, D. Taylor Hendrixson, Joshua Duncan, Mark J. Manary, Kevin B. Stephenson

**Affiliations:** 1 School of Medicine, Washington University, St Louis, Missouri; 2 Project Peanut Butter, Freetown, Sierra Leone; 3 Ministry of Health, Government of Sierra Leone, Freetown, Sierra Leone; 4 Department of Pediatrics, University of Washington, Seattle; 5 Mental Health Coalition-Sierra Leone, Freetown, Sierra Leone

## Abstract

**Importance:**

Perinatal depression is common in sub-Saharan Africa, yet treatment gaps are substantial. Cognitive behavioral therapy (CBT) is effective but relies on written materials.

**Objective:**

To evaluate the efficacy of a culturally adapted CBT intervention delivered by lay counselors to women with perinatal depression in rural Sierra Leone.

**Design, Setting, and Participants:**

This study was an individually randomized, outcomes assessor- and investigator-blinded, clinical trial of an adapted CBT intervention vs enhanced usual care. The trial was conducted in 6 government-run antenatal clinics in Pujehun District, Sierra Leone, which has limited infrastructure, low literacy, high maternal undernutrition, and no perinatal mental health services. Participants were drawn from a concurrent antenatal nutritional supplementation trial between November 2023 and April 2025. Eligible participants were pregnant and postpartum women who were undernourished and had depression (scoring ≥9 on the adapted Patient Health Questionnaire-9 [aPHQ-9]).

**Intervention:**

Participants were randomized 1:1 to receive 6 weekly sessions of CBT adapted for delivery without written materials or only enhanced usual care. All participants received enhanced usual care, which consisted of antenatal nutritional supplementation, monthly malaria chemoprophylaxis, and 2 doses of azithromycin.

**Main Outcomes and Measures:**

The primary outcome was aPHQ-9 score at 8 weeks. Secondary outcomes included clinically meaningful symptom reduction (decrease of >3 points) and remission (aPHQ-9 score <5).

**Results:**

Among 881 women screened, 155 (median [IQR] age, 19 [18 to 22] years) were randomized to CBT (80 women) or control (75 women) groups. Postintervention data were available for 140 of 153 eligible participants (91.5%). Median aPHQ-9 scores at postintervention were lower in the CBT group than the control group (median difference, –4; 95% CI, –5 to –3; *P* < .001). Clinically meaningful symptom reductions were more common with CBT vs control (72 participants [96.0%] vs 36 participants [55.4%]; odds ratio, 19.33; 95% CI, 6.33 to 84.59; *P* < .001), and 59 CBT participants (78.6%) achieved remission compared with 22 participants (33.8%) in the control group (odds ratio, 7.21; 95% CI, 3.39 to 15.33; *P* < .001). Attenuated effects persisted through 9 months after pregnancy (median difference in aPHQ9 for intervention vs control, −1; 95% CI, −2 to 0; *P* = .02).

**Conclusions and Relevance:**

In this study, a culturally adapted CBT intervention delivered by lay counselors reduced depressive symptoms approximately 8 weeks after enrollment compared with enhanced usual care. These findings suggest that CBT adapted for low-literacy settings may offer a scalable model for treatment where mental health professionals are scarce.

**Trial Registration:**

ClinicalTrials.gov Identifier: NCT05949190

## Key Points

**Question:**

Is a culturally adapted cognitive behavioral therapy (CBT) program delivered by lay counselors from the local community efficacious for treating perinatal depression among women who are undernourished?

**Findings:**

In this randomized clinical trial of 155 perinatal women who were undernourished with depression in rural Sierra Leone, participants assigned to receive 6 sessions of CBT had lower depressive symptom scores after treatment compared with those receiving enhanced usual care but no counseling.

**Meaning:**

These findings suggest that CBT adapted for low-literacy settings and delivered by lay counselors may offer a scalable model for evidence-based psychological treatment where mental health professionals are scarce.

## Teaser

This randomized clinical trial investigates the effect of culturally adapted cognitive behavioral therapy delivered by local lay counselors on perinatal depression among women in rural Sierra Leone who were undernourished.

## Introduction

Perinatal mood disorders encompass a spectrum of mental health conditions occurring during pregnancy and up to 1 year after pregnancy, including perinatal depression, anxiety disorders, and psychosis. Perinatal depression, in particular, represents a major global health challenge, with prevalence rates of 10% to 20% in high-income countries and substantially higher rates reported in low- and middle-income countries.^1^ In sub-Saharan Africa, rates of perinatal depression range from 15% to 40%, influenced by poverty, social isolation, intimate partner violence, HIV infection, and limited social support systems.^2,3,4^ Perinatal depression is associated with poor self-care, impaired mother-infant bonding, preterm birth, low birth weight, and compromised offspring physical and neurocognitive development. Access to mental health care remains limited in resource-constrained settings, with treatment gaps exceeding 90% in many sub-Saharan African countries, where cultural stigma around mental health, shortage of trained mental health professionals, and competing health care priorities further compound the challenge.^5,6^

Cognitive behavioral therapy (CBT) has demonstrated efficacy for treating perinatal depression in multiple randomized clinical trials.^7,8,9^ However, most evidence comes from high-income settings with literate populations. Standard CBT protocols rely on written materials and literacy-dependent exercises that may not be feasible or culturally appropriate in low-literacy contexts.

Community-based delivery approaches using trained nonspecialist counselors have shown promise for delivering mental health interventions in resource-limited settings.^6,10,11^ Evidence on perinatal depression programs in rural sub-Saharan Africa remains limited, with few rigorous trials conducted and minimal adaptation for low-literacy populations.^12,13,14,15^ Adaptation requires not only linguistic and cultural translation but also redesign of therapeutic approaches to function without written materials while maintaining core CBT principles.

Undernutrition and depression are bidirectionally associated through shared inflammatory pathways and psychosocial stress, and women who are undernourished have heightened risk for depression yet are less likely to access care.^5,16,17^ We previously developed a CBT intervention adapted for pregnant and postpartum women with depression and low literacy in rural Sierra Leone, a region without mental health care services.^18^ We aimed to evaluate the efficacy of this adapted CBT program in improving perinatal depression compared with enhanced usual care among undernourished perinatal women.

## Methods

The protocol for this randomized clinical trial was approved by the Sierra Leone Ethics and Scientific Review Committee, Pharmacy Board of Sierra Leone, and Washington University Human Research Protection Office. The trial complied with the International Council for Harmonization guidelines for good clinical practice, including local monitoring visits and central data monitoring, and the Consolidated Standards of Reporting Trials (CONSORT) reporting guideline. For participants younger than 18 years who were unmarried, both participant assent and parent or guardian consent were required. Participants provided written informed consent or, for those unable to read, thumbprint consent with an impartial witness present. The full protocol is available in Supplement 1.

### Study Design and Setting

This was an individually randomized clinical, outcomes assessor- and investigator-blinded superiority trial of a CBT program developed for perinatal women with low literacy and depression compared with enhanced usual care without any mental health interventions. This trial (hereafter, *CBT Trial*) was conducted as 1 factor of a 2 × 2 factorial trial in Pujehun District, Sierra Leone, between 2023 and 2025. The parent trial, Improving Cognition and Gestational Duration With Targeted Nutrition (COGENT), included the other factor in the 2 × 2 design and simultaneously tested antenatal supplementation with a ready-to-use supplementary food with or without added fish oil and choline. COGENT and CBT Trial randomizations were independent. All CBT Trial participants were enrolled in COGENT and received nutritional supplementation per their COGENT allocation; CBT trial enrollment was conditional on COGENT enrollment and a qualifying adapted Patient Health Questionnaire-9 (aPHQ-9) score. Only a subset of COGENT participants were enrolled in the CBT Trial.

The CBT trial was conducted at 6 government-run antenatal clinics selected from 12 COGENT sites based on larger catchment populations in Pujehun District. Pujehun is a rural district in southeastern Sierra Leone with no electrical grid, rates of 10% to 15% for undernutrition in pregnancy, low literacy rates, and no existing mental health interventions for perinatal depression at the time of study initiation.

### Participants

CBT Trial participants were perinatal women enrolled in COGENT who had depression at enrollment or at any point during their COGENT participation. All COGENT participants underwent depression screening using the aPHQ-9 at enrollment, monthly during pregnancy, and at 1.5, 3, 6, and 9 months after pregnancy. Women at the 6 selected clinics scoring 9 or greater on the aPHQ-9 at any screening point, including those identified during the postpartum period, were eligible for the CBT trial. Participants were encouraged to present any time if concerned about their mental or physical health.

COGENT eligibility criteria were being pregnant, aged 13 years or older, undernourished (middle upper arm circumference ≤23.0 cm, body mass index [calculated as weight in kilograms divided by height in meters squared] <18.5, or both), willing to adhere to study procedures, and planning to reside in the study area. Exclusion criteria included participation in other supplementary feeding programs, known allergies to study foods or medications, and conditions requiring immediate hospitalization. There were no additional exclusion criteria for the CBT Trial.

### Randomization and Masking

Participants were individually randomized 1:1 to CBT intervention or control using an opaque-envelope manual randomization system (eMethods in Supplement 2). Given the nature of the intervention, participants and treating counselors could not be blinded. Outcome assessors and investigators, including the trial statisticians (D.T.H., M.J.M., and K.B.S.), were blinded to allocation. At each clinic, the counselor who conducted screening, enrollment, and randomization delivered CBT if allocated, while a different counselor was assigned to conduct outcome assessments.

### Study Procedures

The CBT program was developed in 2022 to 2023 in collaboration with the Mental Health Coalition-Sierra Leone (MHC-SL). Four lay counselors with at least some secondary school education were hired based on female sex, Pujehun residency, fluency in the local dialect (Mende), familial experience with perinatal depression, and observed ease establishing rapport with clients. Counselors were not mental health specialists, and prior mental health training was not a qualification for selection. CBT was then adapted for use by clients without literacy. Thought records and written homework were replaced with oral processing, mental rehearsal, role-play, and drawing. Pictorial aids were used to complement symptom assessment (eFigure 1 in Supplement 2). MHC-SL trained counselors during a 2-week intensive training program on depression identification and implementation of this adapted CBT program, followed by role-play practice and supervised field practice with competency assessment prior to seeing participants independently. Ongoing supervision was provided by MHC-SL through monthly 3-day on-site visits to the clinics, during which supervisors directly observed screening and CBT sessions to assess quality and fidelity and held daily debriefing meetings with all counselors. Between visits, counselors were able to contact MHC-SL for ad hoc consultation as needed. Program development is described in detail elsewhere.^18^

The CBT intervention consisted of 6 weekly 45- to 60-minute individual sessions. These sessions followed a structured protocol: (1) problem identification and psychoeducation; (2-3) solution generation and selection using SMART (Specific, Measurable, Attainable, Realistic, Timely) goals; (4-5) evaluation of attempted solutions and skill refinement; and (6) integration and relapse prevention. Between sessions, participants received tailored homework assignments and practiced with counselors to demonstrate comprehension. Sessions occurred primarily at clinics, with occasional home visits when transportation was not feasible (eMethods in Supplement 2).

Control group participants received no mental health intervention. Enhanced usual care consisted of routine antenatal and postnatal care plus antenatal nutritional supplementation, monthly malaria chemoprophylaxis, and single doses of the antibiotic azithromycin in the second and third trimesters of pregnancy through COGENT. The schedule of COGENT study visits is detailed in Supplement 1. A counselor administered monthly aPHQ-9 assessments during pregnancy and at each postpartum study visit, but this was the only counselor contact for participants in the control arm beyond enrollment. No mental health services were available in the study area outside the CBT Trial.

Safety oversight was provided by MHC-SL experts, who were available for immediate referral for aPHQ-9 scores of 20 or greater or participants who reported suicidal ideation, and by the COGENT study local monitor and independent medical reviewer, in accordance with Pharmacy Board of Sierra Leone (PBSL) guidelines. PBSL recommended against establishment of a formal trial steering committee; oversight was provided by the investigator team, local monitoring, and independent ethics review.

### Outcomes

The primary outcome was aPHQ-9 score at postintervention, targeted at 8 weeks after randomization. The PHQ-9 was selected based on its widespread use and validation in sub-Saharan Africa.^19,20,21^ Recently, the PHQ-9 was validated in Sierra Leone, demonstrating a sensitivity of 73.8%, a specificity of 76.2%, and an internal consistency reliability of 0.81.^22^ A cutoff of 9 or greater was selected because validation studies have shown that cutoffs between 8 and 11 provide acceptable diagnostic accuracy for identifying depression.^23,24,25^ A threshold of 9 falls within this validated range and is commonly used in global mental health research to identify individuals with clinically meaningful depressive symptoms who may benefit from low-risk counseling interventions such as CBT.

The aPHQ-9 differs from the PHQ-9 in 4 ways: (1) translation for oral delivery in the local language, Mende; (2) addition of visual aids to help illustrate symptom frequency; (3) removal of question 5 reference to overeating given that all participants were undernourished; and (4) replacement of question 7 “reading newspaper or watching television” with “doing household chores.”^18^ The instrument was written in English and Krio but delivered orally in Mende (eFigure 1 in Supplement 2). These changes were made in consultation with MHC-SL to improve cultural relevance. Translations were iteratively improved with field testing prior to implementation in the CBT Trial.^18^ Like the PHQ-9, the aPHQ-9 consists of 9 items scored 0 (“not at all”) to 3 (“nearly every day”), for a total score of 0 to 27. For participants who missed their target 8-week assessment, postintervention was defined as the aPHQ-9 score collected within a prespecified 7- to 16-week window after randomization, prioritized by proximity to 8 weeks (Supplement 1).

Secondary outcomes were change in aPHQ-9 between baseline and postintervention; a reduction in aPHQ-9 between baseline and postintervention of 3 or less, more than 3, more than 5, or more than 9 points; a reduction between baseline and postintervention of more than 50%, a postintervention aPHQ-9 less than 5; and all aPHQ-9 scores measured as part of routine data collection in COGENT. In a post hoc analysis, aPHQ-9 scores were translated to symptom-days (0 = 0 days; 1 = 3.5 days; 2 = 9 days; 3 = 13 days) over the past 2 weeks. Interassessor reliability of aPHQ-9 assessment was evaluated by having 2 different counselors assess aPHQ-9 scores on a single participant 1 hour apart. All 4 counselors underwent interassessor reliability testing during 2 separate weeks of the trial.

### Statistical Analysis

The study planned to enroll 75 women per arm for a total of 150 participants. Anticipating 10% loss to follow-up, this sample size provided 80% power and a 2-sided α = .05 to detect a 2-point aPHQ-9 difference calculated directly for the Wilcoxon-Mann-Whitney test using G*Power version 3.1.9.7 (Franz Faul, Edgar Erdfelder, Albert-Georg Lang, and Axel Buchner, Heinrich-Heine-Universität Düsseldorf)) (eMethods in Supplement 2). The 10% inflation addressed anticipated attrition but not potential bias from missing data; sensitivity analyses with imputation attempt to address this.

Analyses were conducted in 2 modified intention-to-treat (mITT) populations: participants with postintervention aPHQ-9 data and all correctly enrolled participants. We excluded 2 participants subsequently found not to have been pregnant. The primary estimand was the between-group location shift in postintervention aPHQ-9 scores under a treatment policy strategy (analyzed as randomized regardless of adherence). The Wilcoxon rank-sum test was prespecified as the primary analysis because it makes minimal distributional assumptions appropriate for ordinal, skewed data; it evaluates whether a randomly selected CBT participant is more likely to have a lower aPHQ-9 score than a randomly selected control participant. The Hodges-Lehmann estimator, the natural point estimate associated with the Wilcoxon test, was used to estimate the median of pairwise differences between groups.^26^ A 95% CI was estimated using the normal approximation method with continuity correction. For binary outcomes, odds ratio (ORs) and 95% CIs were estimated using logistic regression. Prespecified covariate-adjusted analyses controlled for maternal age, education, and baseline aPHQ-9. Subgroup analyses were exploratory; the trial was not powered for subgroup comparisons. Detailed analyses are described in the eMethods in Supplement 2. All analyses were conducted using R statistical software version 4.5.0 (R Project for Statistical Computing).^27^

## Results

### Baseline Characteristics

**Figure 1.**
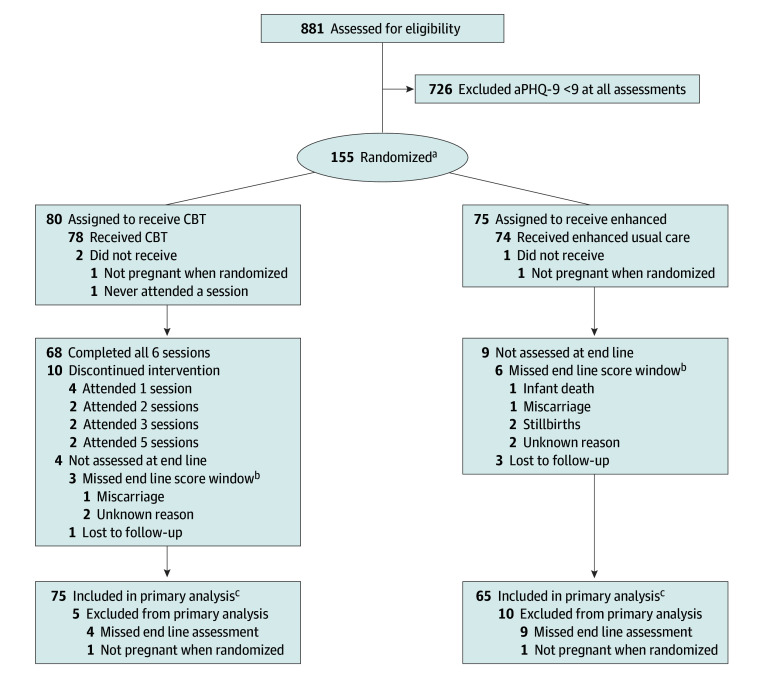
Study Flowchart aPHQ-9 indicates adapted Patient Health Questionnaire-9; CBT, cognitive behavioral therapy. ^a^Of 155 randomized participants, 153 were eligible (2 were excluded because they were not pregnant). Of 153 eligible participants, 134 (87.6%) were enrolled during pregnancy and 19 (12.4%) were enrolled after pregnancy. ^b^Postintervention = aPHQ-9 assessment targeting 8 weeks after randomization (window, 7-16 weeks). ^c^Of 140 participants analyzed, 69 (49.3%) were assessed at exactly 8 weeks (30 in CBT and 39 in enhanced usual care groups).

Between November 8, 2023, and April 28, 2025, a total of 881 women were screened for eligibility, and 155 participants (17.6%; median [IQR] age, 19 [18-22] years) were randomly assigned to the CBT (80 participants) or control (75 participants) group (Figure 1). A total of 153 women were eligible after excluding 2 women who were not pregnant during randomization, with postintervention data available for 140 eligible women (91.5%). Baseline characteristics were similar between groups (Table 1). There were 134 of 153 eligible women (87.6%) enrolled during the antenatal period (Table 1). The self-reported rate of literacy was 38 of 100 participants with literacy data (38.0%). The median (IQR) aPHQ-9 was 11 (10-12). Only 13 of 153 eligible participants (8.5%) were missing postintervention aPHQ-9 data. Baseline characteristics were similar among participants with and without postintervention data (eTable 1 in Supplement 2). Among 79 eligible CBT participants, 68 (86.1%) attended all 6 counseling sessions. No serious adverse events related to the CBT intervention were observed during the trial, and no participants required immediate referral based on aPHQ-9 score of 20 or greater or reported suicidal ideation.

**Table 1.** Selected Baseline Characteristics of Enrolled Participants by Study Arm

Baseline characteristic	Participants, No. (%) (N = 153)	
	CBT (n = 79)	Control (n = 74)
Age, median (IQR), y^a^	19 (18-22)	19 (18-23)
MUAC at enrollment, median (IQR), cm	22.5 (21.8-23.0)	22.4 (21.4-22.9)
Time in supplementary feeding trial before enrollment in this trial, median (IQR), wk^b^	4 (0-10)	4 (0-10)
0	37 (46.9)	33 (44.5)
≤4	14 (17.7)	11 (14.9)
>4	28 (35.4)	30 (40.5)
Pregnancy status at enrollment		
Antenatal	70 (88.6)	64 (86.5)
Gestational age at enrollment among antenatal women, median (IQR), wk^c^	22.5 (18.9-28.7)	21.2 (17.9-26.6)
Postnatal	9 (11.3)	10 (13.5)
Time since delivery at enrollment among postnatal women, median (IQR), wk	13.7 (12.5-19.0)	6.4 (6.0-6.9)
Mother’s highest level of education		
None	14 (17.7)	21 (28.4)
Some primary (grades 1-6)	7 (8.9)	11 (14.9)
≥Secondary	58 (73.4)	42 (56.8)
Currently in school^d^	32 (40.5)	16 (21.6)
History of miscarriage or stillbirth	2 (2.5)	7 (9.5)
No. of adults in household, median (IQR)	4 (3-5)	4 (3-5)
≥2 Children live in household	58 (73.4)	58 (78.4)
Father lives in household	54 (68.4)	47 (63.5)
Household roof made of thatch	10 (12.7)	8 (10.8)
Pit latrine used for stool disposal	67 (84.8)	61 (82.4)
Household has electricity	7 (8.9)	11 (14.9)
Household Food Insecurity Access Scale score (past 4 wk)		
Food secure	2 (2.5)	8 (10.8)
Mildly food insecure	0	1 (1.4)
Moderately food insecure	32 (40.5)	21 (28.4)
Severely food insecure	45 (57.0)	44 (59.5)
aPHQ-9 score at time of enrollment, median (IQR)	11 (10-12)	11 (9-12)
9	11 (13.9)	20 (27.0)
10-12	52 (65.8)	43 (58.1)
≥13	16 (20.3)	9 (12.2)

Abbreviations: aPHQ-9, 9-item adapted Patient Health Questionnaire; CBT, cognitive behavioral therapy; MUAC, middle upper arm circumference.

^a^ There were 2 participants missing from the CBT and 1 participant missing from the control group.

^b^ CBT trial participants are a subset of a larger supplementary feeding study, details of which can be found in Supplement 1.

^c^ There were 2 participants missing from the control group (early pregnancy loss).

^d^ There was 1 participant missing from the control group.

### Primary Outcome

**Figure 2.**
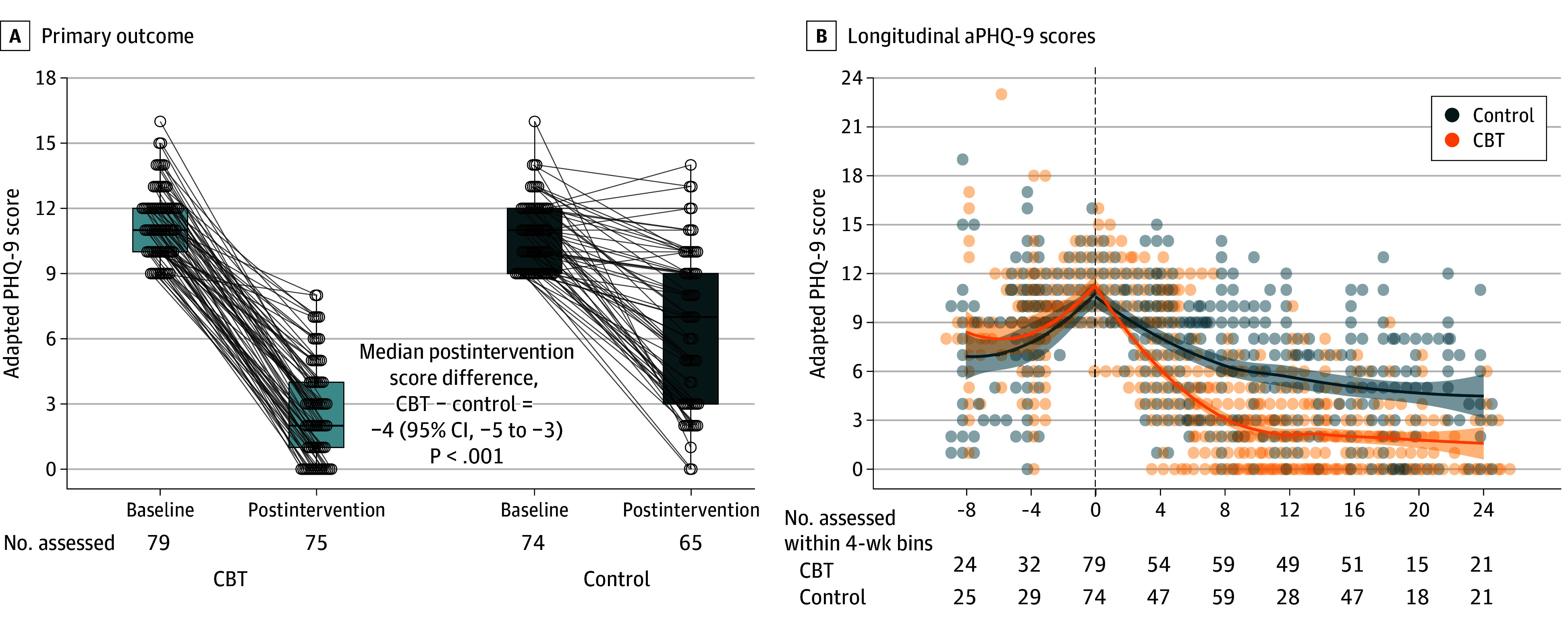
Box and Whisker and Line Graphs of Effect of Intervention on Depressive Symptom Scores A, The central horizontal line of the box plot represents the median adapted Patient Health Questionnaire-9 (aPHQ-9) score, while upper and lower edges of the box represent the IQR and error bars represent 95% of the data range. The dots represent individual scores, which are jittered horizontally in proportion to the number of data points at each score. Lines connect baseline and postintervention scores when both are present. The Hodges-Lehmann median of differences is presented alongside a continuity-corrected 95% CI and *P* value estimated using the Wilcoxon rank-sum test. B, Loess curves were fitted separately for before vs after randomization. Shading around each curve represents an estimated 95% CI. Individual data points are represented as dots. The vertical dashed line at time = 0 indicates the time of randomization. CBT indicates cognitive behavioral therapy.

In the primary mITT analysis among 75 women in the CBT and 65 women in the control group, the median (IQR) aPHQ-9 at 8 weeks was 2 (1-4) in CBT and 7 (3-9) in control groups. The Hodges-Lehmann estimated median of all pairwise differences in postintervention aPHQ-9 scores between CBT and control groups was −4 (95% CI, −5 to −3; *P* < .001), indicating lower scores in the CBT group (Table 2; Figure 2). The probability that a randomly selected CBT participant had a lower postintervention aPHQ-9 score than a randomly selected control participant was 0.80 (95% CI, 0.73 to 0.87).

**Table 2.** Effect of CBT Intervention on Primary and Secondary Outcomes

Outcome	Participants, No. (%) (N = 153)			*P* value^b^
	CBT (n = 79)	Control (n = 74)	Comparison (95% CI)^a^	
Postintervention aPHQ-9				
Participants	75 (94.9)	65 (87.8)	NA	NA
Median (IQR)	2 (1 to 4)	7 (3 to 9)	−4 (−5 to −3)	<.001
Secondary outcomes				
Participants	75 (94.9)	65 (87.8)	NA	NA
Change in aPHQ-9, median (IQR)	−9 (−11 to −7)	−4 (−7 to −2)	−4 (−6 to −3)	<.001
Reduction in aPHQ-9 score from baseline to postintervention, points				
≤3	3 (4.0)	29 (44.6)	0.05 (0.01 to 0.16)	<.001
>3	72 (96.0)	36 (55.4)	19.33 (6.33 to 84.59)	<.001
>5	66 (88.0)	26 (40.0)	11.00 (4.86 to 27.22)	<.001
>9	30 (40.0)	4 (6.2)	10.17 (3.69 to 36.05)	<.001
>50%	68 (90.6)	29 (44.6)	12.06 (5.06 to 32.48)	<.001
Postintervention aPHQ-9 <5	59 (78.6)	22 (33.8)	7.21 (3.39 to 15.33)	<.001
Postnatal outcomes among antenatal enrollees				
Antenatal enrollees	70 (88.6)	64 (86.5)	NA	NA
At 1.5 mo postnatal	62 (88.6)	54 (84.4)	NA	NA
aPHQ-9 score, median (IQR)	2 (0 to 3)	3 (0 to 6)	−1 (−3 to 0)	.009
aPHQ-9 <5	51 (82.3)	30 (55.6)	3.71 (1.63 to 8.90)	.002
At 3 mo postnatal	61 (87.1)	50 (78.1)	NA	NA
aPHQ-9 score, median (IQR)	2 (0 to 3)	4 (1 to 6.75)	−1 (−3 to 0)	.01
aPHQ-9 <5	51 (83.6)	27 (54.0)	4.34 (1.85 to 10.82)	.001
At 6 mo postnatal	55 (78.6)	45 (70.3)	NA	NA
aPHQ-9 score, median (IQR)	2 (0 to 4)	3 (0 to 6)	−1 (−2 to 0)	.12
aPHQ-9 <5	42 (76.3)	28 (62.2)	1.96 (0.83 to 4.74)	.13
At 9 mo postnatal	52 (74.3)	45 (70.3)	NA	NA
aPHQ-9 score, median (IQR)	0 (0 to 3)	2 (0 to 5)	−1 (−2 to 0)	.02
aPHQ-9 <5	46 (88.5)	33 (73.3)	2.79 (0.98 to 8.72)	.06

Abbreviations: aPHQ-9, 9-item adapted Patient Health Questionnaire; CBT, cognitive behavioral therapy; NA, not applicable.

^a^ For continuous outcomes, this represents the Hodges-Lehman median of differences, with 95% CIs calculated using normal approximation with continuity correction. Values less than 0 indicate lower scores for CBT compared with control. For binary outcomes, this represents an odds ratio estimated using logistic regression. Values greater than 1 indicate increased odds for CBT compared with control.

^b^ *P* values were estimated using the Wilcoxon rank-sum test for continuous outcomes and the Wald test for binary outcomes.

### Secondary Outcomes

Among CBT participants, 59 individuals (78.6%) achieved remission (aPHQ-9 < 5) compared with 22 control participants (33.8%) (OR, 7.21; 95% CI, 3.39 to 15.33; *P* < .001) (Table 2). Participants in the CBT group were also more likely to have clinically meaningful (>3 points) reductions in aPHQ-9 scores (72 participants [96.0%] vs 36 participants [55.4%]; OR, 19.33; 95% CI, 6.33 to 84.59; P < .001). Among participants enrolled antenatally, the Hodges-Lehmann estimated difference in postpartum aPHQ-9 scores favored CBT at 1.5 months (−1; 95% CI, −3 to 0; *P* = .009), 3 months (−1; 95% CI, −3 to 0; *P* = .01), and 9 months (−1; 95% CI, −2 to 0; *P* = .02). Remission rates were also higher in the CBT than the control group at 1.5, 3, and 9 months after pregnancy. In a prespecified secondary analysis, adjustment for maternal age, education level, and baseline aPHQ-9 score did not meaningfully alter findings of unadjusted analyses (eTable 2 in Supplement 2). Based on aPHQ-9 frequency ratings over the prior 2 weeks, CBT resulted in a median difference of 1.0 fewer symptoms (95% CI, 0.6 to 1.4 symptoms) per day compared with control (eFigure 2 in Supplement 2).

### Subgroup and Sensitivity Analyses

**Figure 3.**
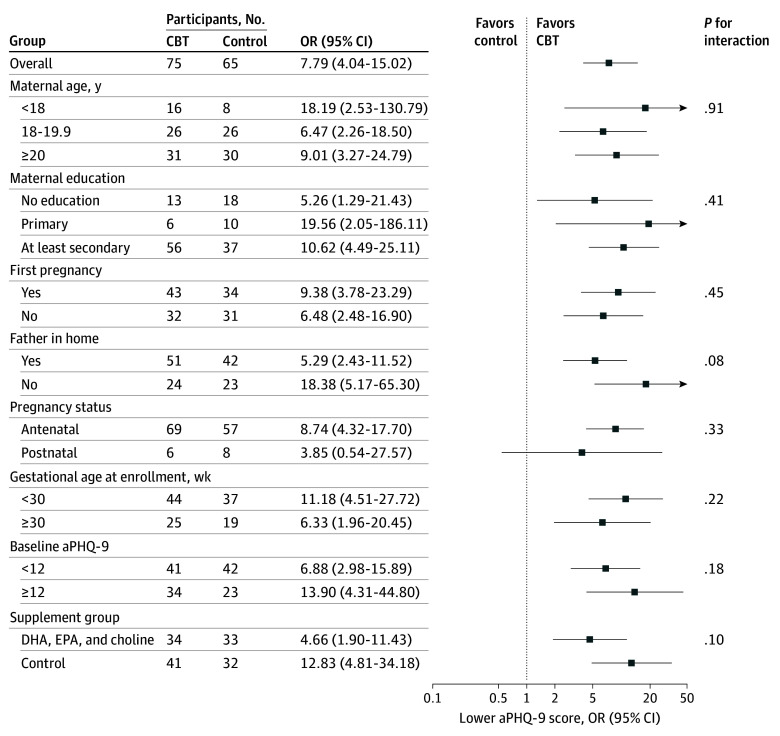
Forest Plot of Odds of Lower Depressive Symptoms Score Odds ratios (ORs) of a lower adapted Patient Health Questionnaire-9 (aPHQ-9) score at postintervention in the cognitive behavioral therapy (CBT) group compared with the control group are shown by selected subgroups. ORs are represented by blue boxes and 95% CIs by lines extending horizontally to each side of blue boxes. These were estimated using ordinal logistic regression and are displayed overall and for each subgroup. *P* values are shown for the interaction term between intervention group and subgroup variable. For maternal age and baseline aPHQ-9 score, the prespecified analysis plan was for modeling these variables as continuous; groupings shown are post hoc to improve visualization. DHA indicates docosahexaenoic acid; EPA, eicosapentaenoic acid.

No effect heterogeneity was identified in subgroup analyses (Figure 3). Participants in the CBT group achieved greater rates of clinically meaningful symptom reduction irrespective of baseline severity (eTable 3 in Supplement 2). In 2 post hoc sensitivity analyses using imputed postintervention aPHQ-9 data for participants without postintervention scores, results were similar to those of the primary mITT analysis (eTables 4-5 in Supplement 2). There was some variability between groups in timing of postintervention assessment, with participants in the CBT group more likely to complete their postintervention assessments at or after 8 weeks compared with participants in the control group (eTable 6 in Supplement 2). In a sensitivity analysis restricted to 69 participants assessed at exactly 8 weeks, results were consistent with the primary analysis (Hodges-Lehmann estimate, −4; 95% CI, −6 to −2; *P* < .001). Participants in the CBT group had lower postintervention aPHQ-9 scores than control participants irrespective of outcome assessment timing (eTable 7 in Supplement 2). Among participants in the CBT group, analysis of postintervention aPHQ-9 score by session attendance was limited by small numbers, with incomplete attendance (eTable 8 in Supplement 2).

### Interassessor Reliability of aPHQ-9 Scores

Interassessor reliability was assessed at 2 times during the trial. At the first point, 71 participants underwent repeat aPHQ-9 testing and the intraclass correlation coefficient was 0.94 (95% CI, 0.91 to 0.97). At the second point, 51 participants underwent repeat aPHQ-9 testing and the intraclass correlation coefficient was 0.91 (95% CI, 0.84 to 0.97).

## Discussion

In this randomized clinical trial of 155 perinatal women in rural Sierra Leone who were undernourished and had depression, a culturally adapted CBT intervention delivered by community counselors led to lower postintervention aPHQ-9 scores compared with enhanced usual care. The estimated 4-point Hodges-Lehmann median difference in postintervention aPHQ-9 scores between CBT and enhanced usual care arms indicates that a typical participant in the CBT group scored 4 points lower than a typical participant in the control group at postintervention. These results were clinically meaningful, with 78.6% of participants in the CBT group achieving remission compared with 33.8% of participants in the control group. The large effect size (OR, 7.21 for remission) likely reflects the treatment-naive population with no baseline mental health services, relatively low baseline severity (median aPHQ-9 = 11), an intensive culturally adapted intervention delivered in the community, and the lack of an attention-matched control. The wide CIs reflect the small sample size.

Our findings are consistent with other community-delivered mental health interventions in more resource-constrained settings. In the Thinking Healthy Programme (THP) in India, peer women delivered a CBT-based intervention that resulted in higher rates of remission from depression at 6 months after pregnancy compared with routine maternal care.^10^ Similarly, the Friendship Bench in Zimbabwe found that village health workers delivering problem-solving therapy for women led to reduced depressive symptoms and lower prevalence of depression in a pre-post pilot evaluation.^28^ This study extends these findings by adapting CBT to rural African participants who were not literate. The intervention was aligned with local beliefs and social structures, incorporating oral processing, role-play, and pictorial methods while maintaining core CBT principles.^18^

For moderate perinatal depression, counseling approaches such as CBT reduce short- and long-term symptoms. These interventions are low risk and may improve accessibility compared with antidepressant medications, which may have adverse effects and for which accessibility is highly setting dependent.^8,29^

To our knowledge, this is the first study to demonstrate sustained benefits in depression at 9 months postpartum follow-up in a rural African population. Unlike prior adaptations that focused on language translation and surface-level modifications, our adaptations specifically addressed literary barriers by including oral delivery methods, pictorial representations, integration with local conceptual frameworks, and accommodation of traditional value systems.^30^ These features may have enhanced skill retention and long-term efficacy. This durability suggests that participants successfully internalized skills taught in the adapted CBT program, which has implications for cost-effectiveness and public health impact.

In the literature, poor maternal mental health during pregnancy and the postpartum period has been associated with adverse birth outcomes, impaired infant growth, and delays in cognitive development.^31,32,33,34,35^ Maternal mental health interventions in resource-constrained settings have been shown to improve children’s developmental and socioemotional outcomes and reduce the risk of growth faltering.^36,37,38,39^ Whether benefits observed in this trial may extend to child outcomes warrants further investigation. Notably, 73.3% of participants who completed follow-up had aPHQ-9 scores less than 5 by 9 months after pregnancy, potentially reflecting the natural course of perinatal depression in this setting, regression to the mean, or benefits of antenatal nutritional supplementation and regular assessment contact. The 17.6% screen-positive rate (155 of 881 participants) is on the lower end of estimates of perinatal depression prevalence in sub-Saharan Africa and may reflect that COGENT participants were receiving fortnightly care and nutritional supplementation from the research team.

In our study, local women were selected and trained to deliver CBT, demonstrating that lay counselors can support maternal mental health interventions. This aligns with successful task-shifting models where community health workers delivered CBT-based interventions for perinatal depression.^11,40,41^ Successful scaling of this model will rely on dedicated funding and integration into existing community and health systems. Future projects should explore large-scale implementation strategies, recognizing that scaling up interventions often comes with the risk of compromising program quality and fidelity. Emerging artificial intelligence (AI)–based applications offer promising ways to support task-shifting CBT delivery at scale. THP, for example, has begun adapting its model to be “technology-assisted, peer-delivered,” in which an AI-enabled virtual avatar guides nonspecialist peers through sessions and reinforces key therapeutic messages.^42,43,44^ This approach incorporates built-in training and supervision and has demonstrated greater effectiveness than THP alone, with the technology designed to function offline to accommodate limited internet or mobile connectivity.

The high retention rate in our study (140 of 153 participants [91.5%]) strengthened our findings. Delivery by local women trained and supervised by the Mental Health Coalition in Sierra Leone further enhanced CBT fidelity. These results are the basis of a new program providing CBT for perinatal depression across several districts in Sierra Leone beginning in 2026. Further research is needed to explore implementation in other settings.

### Limitations

This study has several limitations. Participant blinding could not be achieved, limiting the strength of inference. The lack of an attention-matched control precludes distinguishing specific effects of CBT content from those of counselor attention and therapeutic contact; the observed effect should be interpreted as CBT plus weekly visit attention vs the enhanced usual care of COGENT. Counselors who screened and enrolled participants also delivered the intervention, introducing potential selection bias; this was mitigated by opaque-envelope randomization, outcome assessor blinding, and investigator blinding. Fidelity to the CBT program was not explicitly measured. While adaptations to the PHQ-9 were valuable for this context, they may limit interpretability. The CBT Trial population was conditioned on COGENT enrollment (women who were undernourished), limiting generalizability to nonundernourished populations. Women lost to follow-up in COGENT were not subsequently available for CBT trial enrollment, and their depression trajectories may differ from those of individuals who remained in follow-up. This trial enrolled majority antenatal participants. While subgroup analysis did not identify significant effect heterogeneity by pregnancy status, the small number of participants in the postpartum period limits inference in this subgroup. Results may not generalize beyond rural African settings.

## Conclusions

In this randomized clinical trial, a culturally adapted CBT program reduced perinatal depression among women in rural Sierra Leone who were undernourished. High retention and lay counselor delivery demonstrate feasibility and scalability for low-literacy settings.

## Notes

Supplement 1.
Trial Protocol and Statistical Analysis Plan

Supplement 2. **eMethods.** Sample Size, Randomization, Adapted CBT Intervention Procedure, Data Analysis, and Data Management **eTable 1. **Selected Baseline Characteristics of Enrolled Participants by Study Arm and Presence of Primary Outcome Data **eTable 2. **Adjusted Efficacy Outcomes **eTable 3. **Efficacy Outcomes Stratified by Baseline aPHQ-9 Score **eTable 4. **Efficacy Outcomes From a Sensitivity Analysis Imputing a 2-Point Worsening in aPHQ-9 Scores in Place of Missing Outcome Data **eTable 5. **Efficacy Outcomes From a Sensitivity Analysis Imputing a 9-Point Improvement in aPHQ-9 Scores in Place of Missing Outcome Data **eTable 6. **Distribution of Postintervention Assessment Timing **eTable 7. **Efficacy Outcomes Stratified by Timing of Outcome Assessment **eTable 8. **Efficacy Outcomes Stratified by Session Attendance **eFigure 1. **Adapted PHQ-9 (aPHQ-9) Questionnaire **eFigure 2. **Effect of CBT Displayed in Symptom-Days
eReferences.

Supplement 3.
Data Sharing Statement
