# Supplementary material for: Cognitive Behavioral Therapy for Individuals With Low Literacy and Perinatal Depression: A Randomized Clinical Trial
Source: JAMA Netw Open. 2026 May 7;9(5):e2611101. doi: 10.1001/jamanetworkopen.2026.11101 (PMC13153995; doi:10.1001/jamanetworkopen.2026.11101)
Supplement: Supplement 1. — Trial Protocol and Statistical Analysis Plan [file jamanetwopen-e2611101-s001.pdf]

COGENT - Improving COgnition and GEstational duration with targeted NuTrition

National Clinical Trial (NCT) Identified Number: NCT05949190

Principal Investigator: Mark Manary, MD

Washington University in St. Louis

Funded by: Open Philanthropy

Version Number: 1.3

8 July 2025

Signatures

Date	Name	Signature
8 July 2025	Aminta Shamit Koroma	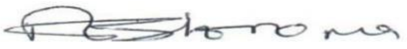
8 July 2025	Mark Manary	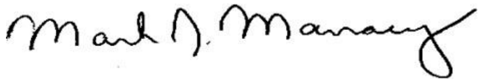
8 July 2025	Kevin Stephenson	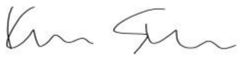

Summary of Changes from Previous Version

Date	Affected Section(s)	Summary of Revisions Made	Rationale
1 August 2023	5.1, 6.2, 8.3, 10.1	Inclusion criteria (age cut-off/marital status for consent vs. assent), add impartial witness for illiterate enrollees, add details for food stability testing	To align with Pharmacy Board of Sierra Leone guidelines
6 March 2024	1.3, 3, 8.1, 9.1, 9.4.3	Addition of serial ultrasound assessments on subgroup of participants	To improve understanding of intrauterine growth in undernutrition
8 July 2025	1.2, 9.2	Increased trial enrollment to 1,660 from 1,600	To ensure adequate statistical power for gestational duration primary outcome

Table of Contents

STATEMENT OF COMPLIANCE 1
1 PROTOCOL SUMMARY 2
1.1 Synopsis 2
1.2 Schema 3
1.3 Schedule of Activities (SoA) 5
2 INTRODUCTION 7
2.1 Study Rationale 7
2.2 Background 7
2.3 Risk/Benefit Assessment 9
2.3.1 Known Potential Risks 9
2.3.2 Known Potential Benefits 10
2.3.3 Assessment of Potential Risks and Benefits 11
3 OBJECTIVES AND ENDPOINTS 12
4 STUDY DESIGN 17
4.1 Overall Design 17
4.2 Scientific Rationale for Study Design 17
4.3 Justification for Dose 18
4.4 End of Study Definition 18
5 STUDY POPULATION 19
5.1 Inclusion Criteria 19
5.2 Exclusion Criteria 19
5.3 Lifestyle Considerations 20
5.4 Screen Failures 20
5.5 Strategies for Recruitment and Retention 20
6 STUDY INTERVENTION 22
6.1 Study Intervention(s) Administration 22
6.1.1 Study Intervention Description 22
6.1.2 Dosing and Administration 24
6.2 Preparation/Handling/Storage/Accountability 24
6.2.1 Acquisition and accountability 24
6.2.2 Formulation, Appearance, Packaging, and Labeling 25
6.2.3 Product Storage and Stability 25
6.2.4 Preparation 26
6.3 Measures to Minimize Bias: Randomization and Blinding 26
6.4 Study Intervention Compliance 27
6.5 Concomitant Therapy 28
6.5.1 Rescue Medicine 28
7 STUDY INTERVENTION DISCONTINUATION AND PARTICIPANT DISCONTINUATION/WITHDRAWAL 29
7.1 Discontinuation of Study Intervention 29
7.2 Participant Discontinuation/Withdrawal from the Study 29
7.3 Lost to Follow-Up 30
8 STUDY ASSESSMENTS AND PROCEDURES 31
8.1 Efficacy Assessments 31
8.2 Safety and Other Assessments 35
8.3 Adverse Events and Serious Adverse Events 36

8.3.1 Definition of Adverse Events (AE) 36
8.3.2 Definition of Serious Adverse Events (SAE) 36
8.3.3 Classification of an Adverse Event 36
8.3.4 Time Period and Frequency for Event Assessment and Follow-Up 38
8.3.5 Adverse Event Reporting 39
8.3.6 Serious Adverse Event Reporting 40
8.3.7 Reporting Events to Participants 41
8.3.8 Events of Special Interest 41
8.3.9 Reporting of Pregnancy 41
8.4 Unanticipated Problems 41
8.4.1 Definition of Unanticipated Problems (UP) 41
8.4.2 Unanticipated Problem Reporting 41
8.4.3 Reporting Unanticipated Problems to Participants 42
9 STATISTICAL CONSIDERATIONS 43
9.1 Statistical Hypotheses 43
9.2 Sample Size Determination 44
9.3 Populations for Analyses 48
9.4 Statistical Analyses 49
9.4.1 General Approach 49
9.4.2 Analysis of the Primary Efficacy Endpoint(s) 49
9.4.3 Analysis of the Secondary Endpoint(s) 50
9.4.4 Safety Analyses 53
9.4.5 Baseline Descriptive Statistics 53
9.4.6 Planned Interim Analyses 53
9.4.7 Sub-Group Analyses 53
9.4.8 Tabulation of Individual participant Data 54
9.4.9 Exploratory Analyses 54
10 SUPPORTING DOCUMENTATION AND OPERATIONAL CONSIDERATIONS 55
10.1 Regulatory, Ethical, and Study Oversight Considerations 55
10.1.1 Informed Consent Process 55
10.1.2 Study Discontinuation and Closure 56
10.1.3 Confidentiality and Privacy 56
10.1.4 Future Use of Stored Specimens and Data 57
10.1.5 Key Roles, Study team, and Study Governance 57
10.1.6 Safety Oversight 58
10.1.7 Clinical Monitoring 58
10.1.8 Quality Assurance and Quality Control 58
10.1.9 Data Handling and Record Keeping 59
10.1.10 Protocol Deviations 59
10.1.11 Publication and Data Sharing Policy 60
10.1.12 Conflict of Interest Policy 60
10.2 Additional Considerations 60
10.3 Abbreviations 61
10.4 Protocol Amendment History 62
11 REFERENCES 63

STATEMENT OF COMPLIANCE

The trial will be conducted in accordance with International Conference on Harmonisation Good Clinical Practice (ICH GCP), the Pharmacy Board of Sierra Leone (PBSL) clinical trial regulations and guidelines, and applicable United States (US) Code of Federal Regulations (CFR). The Principal Investigator will assure that no deviation from or changes to the protocol will take place without prior agreement and documented approval from the Institutional Review Board (IRB) and PBSL, except where necessary to eliminate an immediate hazard to the trial participants.

The protocol, informed consent forms, recruitment materials, and all participant materials will be submitted to the IRB and PBSL for review and approval. Approval of both the protocol and the consent form must be obtained before any participant is enrolled. Any amendment to the protocol will require review and approval by the IRB and PBSL before the changes are implemented to the study. All changes to the consent form will be IRB approved; a determination will be made regarding whether a new consent needs to be obtained from participants who provided consent, using a previously approved consent form.

1 PROTOCOL SUMMARY

1.1 SYNOPSIS

**Title:**	COGENT - Improving COgnition and GEstational duration with targeted NuTrition
**Study Description:**	A randomized, investigator/outcomes assessor-blinded, controlled clinical trial of factorial design among undernourished pregnant women in Sierra Leone testing the hypotheses that (1) adding 500mg docosahexaenoic acid (DHA), 500mg eicosapentaenoic acid (EPA), and 550mg choline to a supplementary food (maternal ready-to-use supplementary food +, M-RUSF+) will prolong gestation and improve infant cognitive development compared with a similar supplementary food but without DHA/EPA/choline (M-RUSF), and (2) a novel cognitive behavioral therapy (CBT) will improve depression among women with ante- or postnatal depression compared with no CBT.
**Objectives:**	Primary Objectives. Comparison 1: To determine whether M-RUSF+ provided to undernourished pregnant Sierra Leonean women will prolong gestation and improve infant cognitive development at 9 months of age when compared with provision of M-RUSF. Comparison 2: To determine whether provision of a novel CBT designed for illiterate end-users will improve depressive symptoms among undernourished pregnant women with ante- or postnatal depression compared with no CBT.<br>Secondary Objectives: To determine whether M-RUSF+ will reduce early and late pre-term birth, improve birth length/weight, reduce low birth weight, improve sub-domain cognitive development scores, reduce neonatal mortality, and reduce depression scores compared with M-RUSF.
**Endpoints:**	Primary Endpoints: Comparison 1: Gestational duration (days), Malawi Developmental Assessment Tool (MDAT) global z-score. Comparison 2: adapted Patient Health Questionnaire-9 score.<br>Secondary Endpoints: Early pre-term (<34 weeks) and late pre-term (<37 weeks) birth, birth weight and length, SGA (<10th %), low birth weight, neonatal mortality, adapted PHQ-9 scores among all participants, MDAT sub-domain scores.
**Study Population:**	1660 pregnant women ≥ 13 years of age with mid-upper arm circumference ≤ 23.0 cm or body-mass index < 18.5 who seek care at a participating antenatal clinic in Kono and Pujehun Districts in Southern Sierra Leone. Gestational duration will be compared among participants with enrollment GA ≤ 30 weeks who have singleton live births.
**Description of Sites Enrolling Participants:**	The study will take place at 30-40 antenatal clinics operated by the Sierra Leonean government.
**Description of Study Intervention:**	The intervention and control supplementary foods will be provided in 100g sachets. They will be composed of peanuts, sugar, palm oil, non-fat milk powder, and pearl millet, for 520 Kcal/day, 18g protein, 33g fat. The intervention food will also contain 500mg DHA, 500mg EPA, and 550mg choline. CBT will be performed weekly for 6 sessions.
**Study Duration:**	30 months
**Participant Duration:**	10-16 months

1.2 SCHEMA

Stage	Activities
Prior to Enrollment	Screen potential participants by inclusion and exclusion criteria. Obtain informed consent.<br>Total N: 1660.
	Randomize
	Arm 1<br>N = 830
	Arm 2<br>N = 830
Visit 1	Baseline demographic, socioeconomic, health questionnaires<br>Perform additional anthropometric measurements // collect blood spot<br>Disburse study food<br>Administer initial study interventions: first trimester (bednet), second or third trimester (azithromycin, malaria chemoprophylaxis, albendazole) **See section 1.3 for full list.**
Visits 2-16 (bi-weekly until birth)	Repeat anthropometry, health questionnaires<br>If enrolled first trimester: Second trimester azithromycin (x1), albendazole (x1), monthly SP // Third trimester azithromycin (x1), monthly SP<br>Disburse study food. **See section 1.3.**
First post-partum visit	Document date of delivery, vital status<br>Maternal and neonate anthropometry, health questionnaires.<br>Cord blood, placental collection. **See section 1.3.**
Post-partum visits 2-4 (6, 12, 24 weeks)	Maternal and child anthropometry<br>Maternal and child health questionnaires. **See section 1.3.**
Post-partum Visit 5 (8-10 months)	Malawi Developmental Assessment Tool testing<br>Maternal and child anthropometry<br>Maternal and child health questionnaires<br>**See section 1.3.**

Following enrollment in M-RUSF+ vs. M-RUSF study

Concurrent: CBT Visits 1-6 (weekly) and, if antenatal, feeding study visits (bi-weekly)

Final visit (8 weeks after enrollment in CBT vs. no CBT comparison)

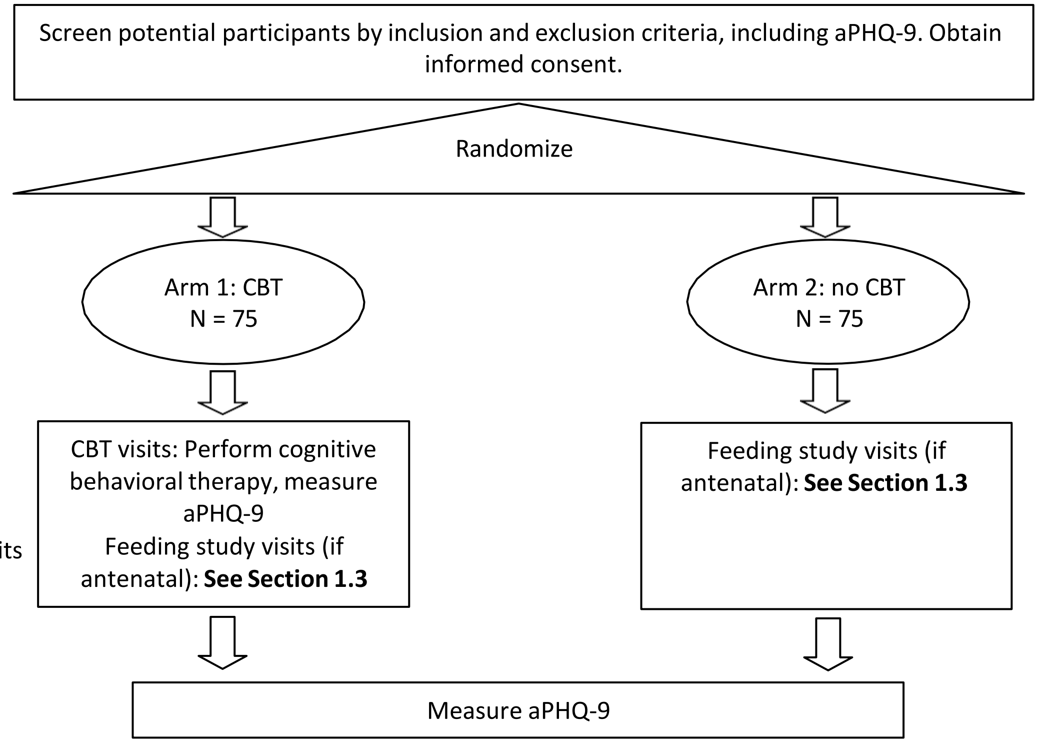

1.3 SCHEDULE OF ACTIVITIES (SOA)

Trial of DHA/EPA/choline-containing supplementary food (M-RUSF+) vs. M-RUSF

	Enrollment (≤ 30 weeks gestation)		Bi-weekly antenatal visits			First postnatal visit	6, 12, 24 weeks postnatal visits	9mo postnatal visit (8 – 10 months)
	1st trimester	2nd/3rd trimester	1st trimester	2nd / 3rd trimester				
**Procedures**				First visit	Subsequent visits			
Informed consent	X							
Demographic questionnaire	X							
Socioeconomic questionnaire								
Medical history	X							
Randomization	X							
Ultrasound gestational age[1]	X							
Disburse study food	X		X	X	X			
Disburse bednets	X							
Azithromycin administration[2]		X		X				
Albendazole administration[2]		X		X				
Sulfadoxine / pyrimethamine[2]		X		X	X (monthly)			
Maternal anthropometry	X		X	X	X	X	X	X
Maternal symptom questionnaire	X		X	X	X	X	X	X
Food insecurity questionnaire	X							
Dietary survey	X							
Blood pressure	X		X	X	X			
Adapted PHQ-9	X		X	X	X (monthly)	X	X	
Blood spot collection[3]	X				X (once)			
Cord blood collection[4]						X		
Placental sample collection						X		
Placental weight measurement						X		
Child anthropometry						X	X	X
Child symptom questionnaire						X	X	X
MDAT								X
Adverse event review and evaluation	X		X	X	X	X	X	X

[1] All participants will undergo US GA assessment at enrollment. A subset of participants enrolled with GA ≤ 24 weeks will undergo serial US assessments every 4-6 weeks.
[2] Albendazole to be given once, either upon enrollment if enrolled during 2nd / 3rd trimester, or at first 2nd trimester visit if enrolled during 1st trimester. Azithromycin to be given 2 times, once during 2nd trimester and once during third trimester. SP to be given monthly starting in 2nd trimester.
[3] A second blood spot will be collected within the 32-36-week gestational age range
[4] Cord blood and placental samples will be collected when possible from mothers at hospitals/clinics with proximity to allow for research staff to be present for collection.

Trial of CBT vs. no CBT for ante- or postnatal depression.

Antenatal Depression. Intervention Group receives CBT. Control = Control Group.

Procedures	Diagnosis visit: aPHQ-9 ≥ 9 Week 0	Week 1	Week 2	Week 3	Week 4	Week 5	Week 6	Post-treatment assessment Week 8**
Mental Health Screening (PHQ-9)	Intervention and Control				Intervention and Control			Intervention and Control
Cognitive Behavioral Therapy	Intervention	Intervention	Intervention	Intervention	Intervention	Intervention	Intervention	
Adverse event review and evaluation	Intervention and Control	Intervention	Intervention	Intervention	Intervention	Intervention	Intervention	Intervention and Control

During the antenatal period, all participants are seen at clinic bi-weekly. They will undergo monthly PHQ-9 assessments. If they are diagnosed with depression, this becomes week 0 in the CBT vs. no CBT study.

* At discretion of counselor

** Target timepoint for endline aPHQ-9 is 8 weeks after enrollment. If this is not obtained, the score at one of the following timepoints and in this order of preference will be used: 9, 7, 10, 11, 12, 13, 14, 15, 16.

Postnatal depression. Intervention Group receives CBT. Control = Control Group.

Procedures	Diagnosis visit: aPHQ-9 ≥ 9 Week 0	Weeks 1-6	Post-treatment assessment Week 8**
Mental Health Screening (aPHQ-9)	Intervention and Control		Intervention and Control
Cognitive Behavioral Therapy		Intervention	
Adverse event review and evaluation	Intervention and Control	Intervention	Intervention and Control

Postnatally, all participants are scheduled for study visits at birth and 6, 12, 24, and 36 weeks after birth. They will undergo aPHQ-9 assessment at each scheduled visit beginning at week 6, which may trigger enrollment in the CBT vs. no CBT study, and may take place when a participant is already enrolled in the CBT vs. no CBT study. Participants will be encouraged to come to clinic if they feel they are developing signs or symptoms of depression, whether on their preset schedule or not.

* At discretion of counselor

** Target timepoint for endline aPHQ-9 is 8 weeks after enrollment. If this is not obtained, the score at one of the following timepoints and in this order of preference will be used: 9, 7, 10, 11, 12, 13, 14, 15, 16.

2 INTRODUCTION

2.1 STUDY RATIONALE

1. Preterm birth remains common worldwide, is the leading risk factor for neonatal mortality, and is associated with short- and long-term developmental complications among those who survive. Many of these deaths occur in Sub-Saharan Africa (SSA); in Sierra Leone, the neonatal mortality rate is 31/1,000 live births.[1] The omega-3 long-chain polyunsaturated fatty acid (PUFA) docosahexaenoic acid (DHA) shows promise in reducing prematurity, but effects have been inconsistent. Choline is a 1-carbon metabolite that plays key roles in DHA trafficking and tissue integration. Maternal undernutrition, which impacts nearly one-quarter of pregnant women in Sub-Saharan Africa, is a state of total-body nutritional depletion that carries increased risks for adverse maternal and fetal outcomes, including preterm birth.[2,3] We hypothesize that adding omega-3 PUFA (DHA+EPA) and choline to a supplementary food in this high-risk population of undernourished pregnant women in rural Sierra Leone will prolong gestation and thereby reduce the most potent risk factor for neonatal mortality and morbidity. A positive finding would have implications for millions of women worldwide, for whom care guidelines are scant.

2. Maternal undernutrition is associated with offspring malnutrition, stunting, and impaired cognitive development. A randomized, controlled, blinded trial published by the PI's research group in 2021 demonstrated that child cognitive development is sensitive to the composition of fatty acid intake during episodes of undernutrition; specifically, cognitive improved when therapeutic food was formulated with lower omega-6 levels and added DHA.[4] We hypothesize adding omega-3 PUFA (DHA+EPA) and choline to a supplementary food provided to undernourished pregnant women in rural Sierra Leone will improve offspring cognitive development at 9 months of age as assessed by the Malawi Developmental Assessment Tool.

3. Ante- and postnatal depression are common yet underrecognized and undertreated in rural SSA, including in Sierra Leone. Cognitive behavioral therapy (CBT) is an evidence-based intervention for perinatal depression, yet current formulations cannot be applied to illiterate populations. In partnership with the Mental Health Coalition of Sierra Leone, the PI developed a novel CBT for use in illiterate populations. We hypothesize that this novel CBT will improve adapted PHQ-9 (aPHQ-9) scores among women diagnosed with ante- or postnatal depression during the aforementioned clinical trial. This will be a factorial trial.

2.2 BACKGROUND

Undernutrition and inflammation in pregnancy

Nearly one-quarter of pregnant women in SSA are undernourished.[2] This is a high-risk state for them and their offspring and requires treatment. Quality supplemental nutrition is an essential but insufficient part of treatment in contexts of high inflammation, such as are common in rural SSA.[5] A recent large, randomized controlled clinical effectiveness trial in Sierra Leone led by the PI of this protocol/trial, hereafter referred to as MamaDutasi, demonstrated a 55% reduction in neonatal mortality as well as longer, heavier newborns when two doses of azithromycin and monthly malaria chemoprophylaxis were added to high-quality supplementary food during pregnancy.[6] Combining this anti-infective treatment

bundle with further improvements in the supplementary food product has the potential to yield even greater benefits for these mothers and their children, as does treatment of ante- and postnatal depression.

Docosahexaenoic acid and choline supplementation in pregnancy

There is a large body of randomized controlled trial (RCT) evidence supporting DHA supplementation in pregnancy, particularly as regards prevention of early preterm birth and especially favoring higher-dose supplementation (>600mg/day) in those with low DHA status at baseline.[7] A 2018 Cochrane review and a 2021 update found that omega-3 PUFA supplementation during pregnancy is likely to reduce preterm birth (analysis included 27,726 participants) and early preterm birth (16,782 participants), prolong gestation (23,359 participants), increase birth weight (20,104 participants), and reduce serious adverse events for neonate/infant, including perinatal death.[8] Findings have not been entirely consistent, however; for example, a large RCT in Australia did not demonstrate a reduction in early preterm birth with omega-3 supplementation, though rates were low and DHA status at baseline was higher than has been seen in other studies.[9,10] The DHA status of undernourished pregnant women in Sierra Leone is unknown. While people in some areas of Sierra Leone may have consistent fish consumption, which would be expected to raise DHA levels depending on type of fish, others do not. It is plausible that DHA status will be low in the setting of undernutrition and pregnancy. Thus, it is possible that supplementation with omega-3 in this likely deficient state might prolong gestation. As gestational duration is tightly linked with neonatal mortality and subsequent development issues, prolonging gestation is a possible path for benefit in this population. The following figure shows this relationship as seen in MamaDutasi (figure unpublished).

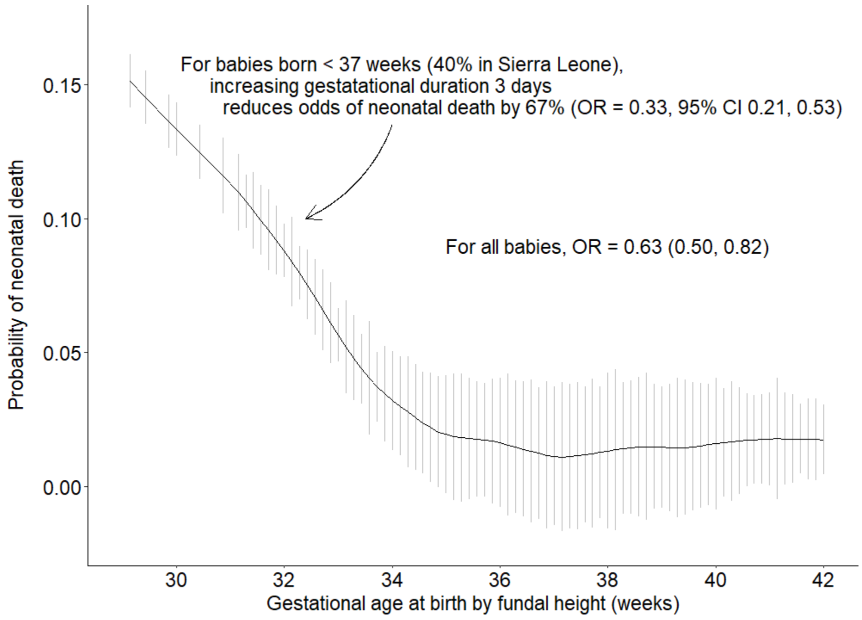

The evidence is more mixed for the effects of antenatal omega-3 supplementation on infant cognitive development. Decades of findings from epidemiological studies and laboratory science, including with animal models, support the essential role of DHA in the structure and function of the brain and retina.[11] Half of the brain's DHA is accumulated during gestation, a process with rapidly increases in the third trimester.[12-14] DHA ultimately composes 10-20% of the brain's lipids by weight. Breast milk contains large amounts of DHA, suggesting its importance for early brain development. In contrast to these lines

of evidence, the 2018 Cochrane review did not find benefit for DHA supplementation in pregnancy across a variety of child cognitive measures.

Two recent studies suggest DHA supplementation's benefits may be context-dependent. An RCT of very pre-term infants demonstrated improved general intelligence 5 years after DHA supplementation during infancy.[15] While supplementation was not antenatal in this study, the results do suggest the importance of DHA during brain development early in life, particularly in the third trimester. The Improved PUFA study, completed by our research group in 2021, offers another such example of cognitive development's sensitivity to fatty acid intake. In the study, children with severe acute malnutrition who received ready-to-use therapeutic food (RUTF) with reduced omega-6 and greater omega-3, including DHA and eicosapentaenoic acid (EPA), demonstrated improved cognitive development compared with a standard RUTF without added omega-3s six months after treatment.[4] Again, while this was not a study of antenatal DHA supplementation, it does support a role for DHA in the setting of undernutrition and likely deficiency. It is possible that DHA transfer to the fetus is inadequate in the setting of maternal undernutrition. Given the rapidity and amount of DHA accumulation in the brain during gestation, this window might present an opportunity for supplementation.

Choline, DHA, and pregnancy

Choline is essential for a variety of physiologic processes, including trafficking and incorporation of DHA into cell membranes. Choline deficiency induces a host of developmental problems in animal studies and has been shown in several small RCTs to improve measures of cognition in children. A recent RCT showed that antenatal choline supplementation improves hepatic DHA export and multiple markers of DHA status in pregnant women.[16] It is possible that co-supplementation with choline and DHA might magnify the benefits of both when given to a population at high risk for deficiencies.

Cognitive behavioral therapy for ante- and postnatal depression

The prevalence of antenatal depression has been found to be approximately 7-20%, while that of postnatal depression is nearly 20%. While prevalence assessments are scarce in Sub-Saharan Africa and non-existent in Sierra Leone, evidence suggests rates are higher in low- and middle-income countries. Both pre- and postnatal depression are associated with failure to seek care, poor diet, use of harmful substances, self-harm and attempted suicide, poor care of offspring and adverse effects on fetal to adolescent development. A 2020 systemic review of 5 RCTs found evidence supporting CBT for antenatal depression.[17] A 2016 systemic review found evidence for increased likelihood of remission with CBT among women with ante- and postnatal depression compared with usual care.[18] A 2018 systematic review of 20 RCTs found that CBT improved postnatal depression.[19] In all cases, CBT was applied in literate populations. Whether a novel CBT designed for use in the setting of illiteracy might benefit pregnant women with ante- or postnatal depression is unknown.

2.3 RISK/BENEFIT ASSESSMENT

2.3.1 KNOWN POTENTIAL RISKS

With respect to omega-3 PUFA/choline addition to a supplementary food, we do not anticipate any harms to participants. The amount of omega-3 PUFA to be given, 1g/day of DHA+EPA, is lower than what is contained in a 6-ounce serving of salmon. Similarly, the 550mg of choline to be included approximates 4 eggs' worth. In addition, the target population – undernourished pregnant women – is

likely to be deficient in these nutrients, as they are undernourished and their diets contain few animal-source foods. Thus, any theoretical risks of over-supplementation are unlikely. In addition, omega-3 PUFAs have been studied as pregnancy supplements in RCTs including over 23,000 women. A 2018 Cochrane review found no evidence for harm in either mothers or offspring.[7] Below, several considerations are explicated in more detail.

Omega-3 PUFA might increase the incidence of pregnancies continuing beyond 42 weeks gestation, which itself increases risk for adverse birth outcomes. Among >16,000 RCT participants included in a 2021 meta-analysis, the relative risk for >42 weeks gestation with omega-3 supplementation vs. none was 1.31 (1.01, 1.70).[8] It is unclear, though, whether this potential risk would translate to undernourished pregnant women in Sierra Leone, a population different from those included in the studies which were meta-analyzed. Because of a lack of ultrasound capabilities, quality data on gestational duration in Sierra Leone are not available. In our recent RCT, fundal height was used to estimate gestational age (Hendrixson); only 9/1431 participants had final fundal heights estimated at > 42 weeks. Indeed, the issue motivating this clinical trial is the suspected high incidence of pre-term birth, suggested by 82% of participants in the above trial with final fundal height < 37cm and ~20% rate of LBW. Thus, we estimate any risk of adverse events related to prolonged gestation to be minimal.

A 1992 RCT identified a trend of greater blood loss during delivery with omega-3 PUFA supplementation.[20] Supplementation in this trial was at a higher dose, 2.7g/day, than will be given in COGENT. This safety concern has not been replicated in subsequent large RCTs, which have used doses closer COGENT's 1g/day.

Several trials that used fish-source omega-3s reported higher rates gastrointestinal symptoms in pregnant women, including burping, constipation, and diarrhea. These symptoms will be tracked among participants.

Several trials have followed participants and their offspring greater than 10 years after the trial. No longer term / delayed risks have been identified.

Choline intakes that are greater than an order of magnitude higher than the supplementation planned in this trial have been associated with hypotension, body odor, sweating, vomiting, and salivation.[21] Doses from involved studies ranged from 7.5 – 16 g daily. The tolerable upper intake level for adults has been set at 3.5 g daily. The dose used in this study will be 550mg/day, as justified in **section 4.3.**

With respect to CBT for ante- and postnatal depression, there is the potential for therapy to cause harm in participants. CBT involves uncovering, probing, and working to gain control over unproductive thought patterns and behaviors. This can cause distress by its nature, as it asks participants to confront negative thoughts, emotions, and physical responses to, for instance, anxiety and depression. A 2018 study of 100 CBT practitioners showed, in the therapists' perceptions, 27% of patients experience distress and 9% worsening of symptoms, with 21% of such effects being severe/very severe, though only 5% were persistent.[22] Of note, nearly half of patients referenced had undergone psychiatric hospitalization and were on medications for a psychiatric condition, suggesting more severe mental health issues than might be seen in this trial's population. However, it is possible that some participants may experience distress, and possibly temporary worsening of depression, as a result of receiving CBT.

2.3.2 KNOWN POTENTIAL BENEFITS

As identified in a 2018 Cochrane review and 2021 update, omega-3 LCPUFA supplementation during pregnancy is likely to reduce preterm birth (27,726 participants) and early preterm birth (16,782 participants), prolong gestation (23,359 participants), increase birth weight (20,104 participants), and reduce serious adverse events for neonate/infant, including perinatal death.

We hypothesize that supplementation of DHA/EPA during pregnancy in undernourished women could impart cognitive developmental benefits in offspring. Decades of epidemiological and animal studies research supports the essential role of DHA in cognitive development and high rate of accretion in the brain during gestation. While previous human studies research has shown inconsistent effects of antenatal DHA supplementation, none of this research was done among undernourished pregnant women.

A 2021 Cochrane review found low certainty evidence of a small benefit of omega-3 PUFAs for depressive symptoms compared to placebo. The evidence was stronger for supplementation with more EPA. We hypothesize that 500mg/500mg DHA/EPA supplementation during pregnancy might reduce aPHQ-9 scores and incidence of ante- and postnatal depression among women likely to be deficient in omega-3 PUFAs.

The 3 systematic reviews previously detailed demonstrate evidence for benefit of CBT on ante- and/or postnatal depression.[17-19]

2.3.3 ASSESSMENT OF POTENTIAL RISKS AND BENEFITS

Everyday consumption of omega-3 PUFAs and choline in some populations in amounts greater than the proposed supplementation in COGENT argues strongly in favor of the safety of this intervention, as do several large RCTs documenting safety. In addition, the target population of undernourished pregnant women are likely to be deficient in both nutrients, further reducing the theoretical risk of over-supplementation. In contrast, the evidence in favor of omega-3 PUFAs for prolonging gestation, increasing birth weight, and reducing serious adverse neonatal events is strong. The potential benefits outweigh the marginal risks.

Regarding offspring cognitive development, there is no evidence of harm to offspring with antenatal supplementation. While the likelihood of benefit is less clear than in the case of prolonging gestation, were it found to be beneficial, this would have significant implications for millions of women suffering from undernutrition, and their children.

While CBT has been associated with distress in patients, 25 RCTs have documented efficacy in ante- and postnatal depression without revealing concerning safety signals. Left untreated, ante- and postnatal depression can have devastating effects on mom and offspring. If the novel CBT were found to be beneficial, it could have implications for millions of women with untreated ante- and postnatal depression who do not have therapeutic options available. The potential benefits outweigh the marginal risks.

3 OBJECTIVES AND ENDPOINTS

OBJECTIVES	ENDPOINTS	JUSTIFICATION FOR ENDPOINTS
Primary		
To determine whether adding 1g DHA/EPA + 550mg choline to a supplementary food provided to undernourished pregnant Sierra Leonean women ≤ 30 weeks GA at enrollment will prolong gestation.	Gestational duration, days	In settings of high rates of prematurity, prolonging gestation improves offspring outcomes. Based on recent RCT data, prolonging gestation by 3 days reduced odds of neonatal death by 67%.
To determine whether adding 1g DHA/EPA + 550mg choline to a supplementary food provided to undernourished pregnant Sierra Leonean women will improve offspring cognitive development.	Malawi Developmental Assessment Tool global z-score	By combining gross motor, fine motor, language, and social developmental assessments, MDAT provides a broad measure of child development. MDAT was developed in Malawi and has been validated in other Sub-Saharan African settings.
To determine whether provision of a novel cognitive behavioral therapy intervention will improve depressive symptoms among women with ante- or postnatal depression.	Adapted Patient Health Questionnaire-9 (PHQ-9) score	PHQ-9 has been validated as a screening and diagnostic tool for ante- and postnatal depression, including in Sub-Saharan Africa. It is easy to use, has been translated to many settings, and has demonstrated good sensitivity to changes in depression over time. PHQ-9 is a patient-centered outcome.
Secondary		
To determine whether adding 1g DHA/EPA + 550mg choline to a supplementary food provided to undernourished pregnant Sierra Leonean women ≤ 30 weeks GA at enrollment will:		
• Reduce early pre-term delivery.	Proportion of deliveries < 34 weeks gestation	ePTB carries a high risk of neonatal morbidity and mortality as well as neurodevelopmental sequelae.
• Improve birth weight and length	Birth weight (g), length (cm)	Birth weight predicts neonatal outcomes; birth length predicts stunting risk

OBJECTIVES	ENDPOINTS	JUSTIFICATION FOR ENDPOINTS
• Reduce LBW	Proportion of deliveries < 2.5kg	LBW is a risk factor for neonatal mortality
• Reduce neonatal mortality	Proportion of deaths < 28 days from delivery	Neonatal death rate is high in Sierra Leone.
• Reduce pre-term birth	Proportion of deliveries < 37 weeks	While not as severe as ePTB, still carries increased risk
• Reduce small-for-gestational age	<10th % birth weight-for-GA.	SGA expected to be more common in undernourished pregnant women
• Increase placental weight	Placental weight (g)	Cause/marker of intrauterine growth restriction
• Reduce depression symptoms	Adapted Patient Health Questionnaire-9 score (aPHQ-9)	As stated under primary objectives.
• Increase maternal and fetal DHA status	DHA in maternal and cord blood	Marker of accretion of DHA and adherence to intervention
• Improve sub-domains of cognitive development	MDAT gross motor, fine motor, language, social sub-domains	Assessment of individual domains to assess possible differential effects of intervention
• Increase post-term delivery	Proportion of deliveries > 42 weeks	Potential adverse effect of omega-3 supplementation in pregnancy
To determine whether adding 1g DHA/EPA + 550mg choline to a supplementary food provided to undernourished pregnant Sierra Leonean women **regardless of GA at enrollment** will:<br>• Prolong gestation<br>• Reduce early pre-term birth<br>• Improve maternal aPHQ-9 scores<br>• Improve birth weight and length<br>• Reduce LBW		
To determine whether provision of a novel cognitive behavioral		

OBJECTIVES	ENDPOINTS	JUSTIFICATION FOR ENDPOINTS
therapy intervention among women with ante- or postnatal depression will:		
• Change in aPHQ-9 from baseline	Endline – baseline aPHQ-9	Accounts for baseline degree of depression
• Improve aPHQ-9 from baseline beyond certain thresholds	Endline aPHQ-9 reduced by ≤ 3, >3, >5, >9, >50%	Common thresholds used to measure effectiveness of depression interventions, accounting for baseline
• Reduce depression at aPHQ-9 of 9	aPHQ-9 ≥ 9	
• Reduce depression at aPHQ-9 of 5	aPHQ-9 < 5	
• Reduce aPHQ-9 postnatal (among antepartum enrollees)	aPHQ-9 score at 6 weeks, 3 mo, 6 mo, 9 mo	To assess whether CBT has effects that persist beyond birth when given antepartum
• Prolong gestation (among antepartum enrollees)	Gestational duration, days	
• Improve gestational weight gain (among antepartum)	Weight gained between CBT and last measurement before delivery	
• Improve offspring birth weight, length		
• Improve infant cognitive development<br><br>(key subgroups are ante vs. postnatal depression, maternal age, maternal education)	MDAT z-scores	
Tertiary/Exploratory		
To determine whether adding 1g DHA/EPA + 550mg choline to a supplementary food provided to undernourished pregnant Sierra Leonean women ≤ 30 weeks GA at enrollment will:		

OBJECTIVES	ENDPOINTS	JUSTIFICATION FOR ENDPOINTS
• Prolong gestation among subgroups by:<br>○ Baseline DHA status<br>○ Baseline MUAC<br>○ Baseline gestational age<br>○ Parity<br>○ Maternal age	Gestational duration, days	Prior research has revealed possible differential effect of omega-3 by maternal DHA status. Parity and maternal age are predictors of gestational duration.
• Reduce incidence of depression	aPHQ-9 ≥ 9	Cut-off commonly used to diagnose depression
• Increase birth chest and head circumference	Chest circumference (cm), head circumference (cm)	Birth chest circumference predicts neonatal and infant mortality
• Alter placental tissue histology	Histological differences in e.g. vascularity	Potential mechanism through which DHA / choline might cause benefit
• Increase gastrointestinal symptoms in mothers	Burping, constipation, diarrhea, vomiting	Patient-centered adverse effects
To determine whether adding 1g DHA/EPA + 550mg choline to a supplementary food provided to a subset of undernourished pregnant Sierra Leonean women ≤ 24 weeks GA at enrollment will:		
• Increase intrauterine growth	Estimated fetal weight, head circumference, femur length on serial USs	DHA may increase birth weight and length via promoting greater intrauterine growth
To determine whether provision of a novel cognitive behavioral therapy intervention among women with ante- or postnatal depression, in combination with DHA/EPA and 550mg of choline, will:		
• Improve ante/postnatal depression	aPHQ-9	It is possible that CBT and **EPA**/DHA/choline will interact to induce further benefits.
• Prolong gestation	Gestational duration, days	

OBJECTIVES	ENDPOINTS	JUSTIFICATION FOR ENDPOINTS
• Improve gestational weight gain<br>• Improve infant cognitive development	Weight gained between CBT and last measurement before delivery<br>MDAT	

4 STUDY DESIGN

4.1 OVERALL DESIGN

We hypothesize that adding 500mg DHA, 500mg EPA, and 550mg choline to a supplementary food (M-RUSF+) provided to undernourished pregnant women in rural Sierra Leone will (1) prolong gestation and (2) improve offspring Malawi Developmental Assessment Tool global z-scores at 9 months of age compared with provision of a similar supplementary food but without DHA/EPA/choline (M-RUSF), and that (3) provision of a novel cognitive behavioral therapy (CBT) program to women with ante- or postnatal depression will reduce depressive symptoms.

This will be a 2x2 factorial, randomized, controlled, partially blinded superiority trial. All participants will be individually randomized, 1:1, to receive either M-RUSF+ or M-RUSF. A subset of participants (enrolled until sample size achieved) who are found to have aPHQ-9 scores ≥ 9 will be individually randomized, 1:1, either to receive (intervention) or not receive (control) a novel CBT program. The table below summarizes the structure.

Depression status	CBT vs. none	M-RUSF+	M-RUSF
aPHQ-9 ≥ 9	CBT		
	No CBT		
aPHQ-9 < 9	No CBT		

The M-RUSF+ vs. M-RUSF comparison will be fully blinded. Care was taken in food design to minimize differences in taste, aftertaste, and texture between the foods. Outcomes assessors will be blinded. In the CBT vs. none comparison, participants and therapists will not be blinded. Outcomes assessors will be blinded, as aPHQ-9 assessments will take place at clinic and not by therapy staff. In both cases, the data analysis team will be blinded.

M-RUSF and M-RUSF+ will be composed of whey protein isolate, skim milk powder, peanuts, palm oil, pearl millet, sugar, and multiple micronutrient mix. M-RUSF+ will also contain 1.7g of fish oil (500mg DHA+ 500mg EPA) and 550mg choline given as choline chloride. CBT will involve 6 weekly 45-minute to hour-long sessions.

This trial will take place across 20-30 government-run antenatal clinics in Pujehun and Kono Districts, Southern Sierra Leone. Total clinic number will depend on rate of accrual.

4.2 SCIENTIFIC RATIONALE FOR STUDY DESIGN

M-RUSF functions as a placebo control. It contains the same ingredients as M-RUSF+ except without DHA/EPA/choline. Care will be taken in food design to minimize differences in taste, after-taste, and texture. Because these ingredients cost money, the trial is designed to demonstrate superiority. CBT will not have a control. Because CBT takes time and effort, the comparison is designed to demonstrate superiority.

4.3 JUSTIFICATION FOR DOSE

The Institute of Medicine does not set a daily recommended intake for DHA in pregnancy. 200mg / day is recommended by some expert groups (Simopoulos 2000, FAO WHO 2004, Koletzko 2007).[23,24] Trials that have found an effect of omega-3 PUFA supplementation on gestational duration / early preterm birth have provided >600 DHA. These include Olsen 1992, DOMInO, KUDOS, and ADORE.[20,25-27] There is uncertainty as to the amount of omega-3 PUFA consumed across Sierra Leone, with likely substantial variability primarily driven by differential access to fish. We expect undernourished women to have low intake and levels of omega-3 PUFA. We will provide 500mg/500mg DHA/EPA, expecting that significant amounts of EPA will be converted to DHA but still allowing the opportunity for EPA supplementation to improve depressive symptoms (secondary outcome).

There is uncertainty as to the ideal recommended dose of choline for pregnant women. The uncertainty is greater still in the setting of undernutrition. The Institute of Medicine recommends pregnant women to consume 450mg choline daily, on average. Subsequent to this recommendation, research suggests that the 25mg increase in recommended dose for women who are pregnant (from 425mg for non-pregnant women) is insufficient to account for greater physiologic demands for choline’s functions during pregnancy.[28] A 2022 trial found that 550mg of choline improved hepatic DHA export and biomarkers of DHA status among pregnant patients consuming DHA.[16] We expect undernourished women in Sierra Leone to have low intakes and levels of choline, as the main sources are animal-source foods including milk and eggs. Thus, recognizing the lack of certainty around what constitutes an adequate dose, we will supplement 550mg of choline daily, which is much lower than doses with documented toxicity.

Albendazole (400mg), azithromycin (1g x2), and sulfadoxine/pyrimethamine (1,500mg/75mg monthly) will be provided at standard doses for use in pregnancy. WHO recommends 400mg albendazole after the first trimester in endemic regions, which includes Sierra Leone. WHO recommends at-least monthly SP at 1,500mg/75mg dose starting in the second trimester in endemic regions, which includes Sierra Leone. These doses were used in our prior RCT among undernourished pregnant women in Sierra Leone without issue.

Azithromycin is not guideline-recommended during pregnancy absent a specific indication. It has, however, been given in the second and third trimesters of pregnancy in multiple RCTs, 7 of which took place in low- and middle-income countries and included >1,200 participants each.[6] Several identified improvements in birth weight, LBW, and neonatal mortality, including MamaDutasi. Given the success of this study, and in light of prior positive findings, we will provide 1g of azithromycin in the second and third trimesters to all participants. The 1g dose is routine.

4.4 END OF STUDY DEFINITION

A participant is considered to have completed the study if she has completed all phases of the study including the last visit as shown in the Schedule of Activities (SoA), Section 1.3.

The end of the study is defined as completion of the last visit or procedure shown in the SoA in the trial globally.

5 STUDY POPULATION

5.1 INCLUSION CRITERIA

All women ≥ 13 years of age who present to a participating antenatal clinic, think they are pregnant, and express a willingness to undergo screening for the trial, will undergo screening. This will first include MUAC and BMI assessment. If a participant is pregnant and meets MUAC/BMI criteria for enrollment, they will be invited to undergo full inclusion/exclusion criteria assessment, consent, and randomization. For girls < 18 years of age who are not married, participation will require guardian/parent consent.

In order to be eligible to participate in the M-RUSF+ vs. M-RUSF element of the study, an individual must meet all of the following criteria:

1. Provision of signed (or thumb-printed) and dated informed consent form
    - 1a. Women ≥ 18 years of age or who are married will be allowed to consent for themselves
    - 1b. Women < 18 years of age and who are unmarried must provide assent and a parent or guardian must provide consent
2. Stated willingness to comply with all study procedures and availability for the duration of the study, including no plan to move from the catchment area of a participating clinic
3. ≥ 13 years of age
4. Pregnant
5. Mid-upper arm circumference ≤ 23 cm or body-mass index < 18.5

In order to be eligible to participate in the CBT vs. no CBT element of the study (factorial design with the above), an individual must be enrolled in the M-RUSF+ vs. M-RUSF study and meet the following criteria:

1. Provision of signed (or thumb-printed) and dated informed consent form
    - 1a. Women ≥ 18 years of age or who are married will be allowed to consent for themselves
    - 1b. Women < 18 years of age and who are unmarried must assent and a parent or guardian consent must provide consent
2. Stated willingness to comply with all study procedures and availability for the duration of the study, including no plan to move from the catchment area of a participating clinic
3. Patient Health Questionnaire-9 score ≥ 9

All undernourished pregnant women presenting to participating antenatal clinics, regardless of inclusion/exclusion criteria (aside from allergy to food/medications), will be offered the entire package of care included in the clinical trial.

5.2 EXCLUSION CRITERIA

An individual who meets any of the following criteria will be excluded from participation in this study:

1. Participation in a concomitant supplementary feeding program

2. Known allergy to components of intervention or control study food or medications
3. Known gestational diabetes
4. Hypertension
5. Severe anemia, or other condition requiring immediate hospitalization

5.3 LIFESTYLE CONSIDERATIONS

There will be no special lifestyle recommendations for this trial.

5.4 SCREEN FAILURES

Screen failures are defined as participants who undergo screening but are not enrolled in the study. Most commonly, this is because they do not meet inclusion/exclusion criteria. Screen failure details will be collected throughout the trial to meet the Consolidated Standards of Reporting Trials (CONSORT) publishing requirements and to respond to queries from regulatory authorities.

Individuals who do not meet the criteria for participation in this trial (screen failure) because of MUAC/BMI above cut-offs for inclusion, age <13 years, or because they are not pregnant, may be rescreened. Individuals who do not meet criteria for participation because of a condition requiring immediate hospitalization may be rescreened.

5.5 STRATEGIES FOR RECRUITMENT AND RETENTION

The target sample size is 1660. All participants will be undernourished, pregnant Sierra Leoneans, who would meet several criteria for vulnerability and under-represented status in clinical trials. In addition, we will include adolescent pregnant persons ≥ 13 years of age.

We anticipate accruing 70-80 participants / month. 30-40 government-run antenatal clinics in Kono and Pujehun Districts, Sierra Leone, will be included as sites of screening, enrollment, and trial operations. These clinics will be chosen in agreement with the District Medical Officers for each district and based on recruitment rates seen in the prior RCT by our group (Pujehun District) or census of pregnant women over the year preceding trial initiation (Kono District). In anticipation of the trial, clinic-catchment and village-level sensitization will be done by trial staff via meetings in local chiefs and elders, community health workers, and outreach visits to villages.

Participants may be involved in the trial for up to 16 months. During pregnancy, retention will be encouraged with bi-weekly visits at antenatal clinic, at which time health assessments will be done and supplementary food, recommended antenatal care, and study medications will be disbursed. Our prior experience with a similar study in a similar population suggests that receipt of food and medications will be a strong means of retention. We will also obtain directions from clinic to home for each participant to allow for visits by community health workers in the event of missed clinic visits (see **section 7.3**). We will obtain telephone numbers for the participant and a neighbor/family member. We will provide pre-paid telephone units with instructions to call clinic staff near the time of delivery. These measures are designed to increase retention and meet trial procedures for post-delivery assessments. In addition,

participants will be given gifts of appreciation at enrollment, delivery, and all post-partum visits. Gifts are generally cloth wraps used for many purposes by women in Sierra Leone. Gifts will be given at each post-partum visit because participants will no longer be receiving food or medication after delivery, and thus incentives are required to maintain retention leading up to evaluation of the co-primary outcome MDAT at offspring 9 months of age.

6 STUDY INTERVENTION

6.1 STUDY INTERVENTION(S) ADMINISTRATION

6.1.1 STUDY INTERVENTION DESCRIPTION

Undernourished pregnant women are recommended by the WHO to receive a balanced energy and protein supplement. All pregnant women are recommended by WHO to receive daily iron (30-60mg of elemental iron) and folic acid supplementation (0.4mg). In populations with low dietary calcium intake, 1.5-2g per day of oral elemental calcium is recommended. There is strong evidence for multiple micronutrient supplementation in pregnancy. Otherwise, there are no international guidelines for nutritional supplementation in undernourished pregnant women.

We have modeled the intervention and control supplementary foods on those used in two previous RCTs by our group treating undernourished pregnant women in Malawi and Sierra Leone. Both foods were well-accepted by participants. A formal acceptability trial will be undertaken prior to main trial initiation to assess the acceptability of both M-RUSF+ and M-RUSF.

Both foods will be balanced protein-energy supplements and contain recommended micronutrients. Both were designed at the Food Laboratory of Mark Manary (Trial PI) at Washington University in St. Louis. Ingredients are listed in the table below. The micronutrient premix included in the RUSFs will provide the same quantities of micronutrients as the UNICEF/WHO/United Nations multiple micronutrient supplement for pregnant and lactating women (UNIMMAP), with additional calcium and magnesium.

Ingredient, g/100g	M-RUSF+	M-RUSF
Pearl millet	7.5	7.5
Non-fat dry milk	21.5	21.5
Whey protein isolate	6.8	6.8
Palm oil	23.8	26.3
Brown sugar	18	18
Peanut	15.9	15.9
Fish oil (Polaris)	1.7	0
Choline chloride	0.825	0
Multiple micronutrient premix (DSM)	4	4

The nutrient content of both foods is listed in the table below.

Nutrient	M-RUSF+, 100g	M-RUSF, 100g	Recommended daily allowance[29-31]
Energy, Kcal	530	540	
Protein, g	18.7	18.7	
Fat, g	33.7	34.5	
n6 Polyunsaturated fatty acids, g	6.5	6.5	
n3 Polyunsaturated fatty acids, g	1.3	0.3	
Vitamin A, mg	770	770	770

Vitamin B1/Thiamine, mg	2.8	2.8	1.4
Vitamin B2/Riboflavin, mg	2.8	2.8	1.4
Vitamin B3/Niacin, mg	32	32	18
Vitamin B6, mg	3.8	3.8	1.9
Vitamin B12, mg	5.2	5.2	2.6
Folic acid, mg	500	500	400
Vitamin C, mg	170	170	85
Vitamin D, mg	30	30	15
Vitamin E, mg	30	30	15
Iron, mg	30	30	27
Zinc, mg	22	22	11
Calcium, mg	1600	1600	1000
Chromium, mg	60	60	30
Copper, mg	2000	2000	1000
Iodine, mg	300	300	220
Magnesium, mg	300	300	350
Phosphorus, mg	687.5	687.5	700
Potassium, mg	892.7	892.7	4700
Selenium, mg	120	120	60

Both foods are light-brown-colored peanut-based pastes. They will be produced at the Project Peanut Butter (PPB) factory in Freetown, Sierra Leone. They will be nitrogen vacuum-sealed in foil sachets. The sachets will be identical in appearance aside from a sticker with a color signifying study group (M-RUSF+ vs. M-RUSF), the code for which will only be known by the staff responsible for packaging.

Each batch of M-RUSF+ and M-RUSF will meet UNICEF specifications for aflatoxin, Enterobacter sp., and Salmonella contamination. Boxes of sachets will be kept in a PPB storage room that is maintained at < 25°C until transfer to the research team for use at clinic.

The novel CBT program will consist of 6 hour-long sessions. Below is a summary of what might occur during a treatment program along with a few examples, though each treatment plan is tailored to the individual at hand by the counselor and participant. Beginning in session 1, a key goal is to create an enabling and empowering environment for women to talk openly and with assurance of confidentiality. Session 1 is also devoted to problem identification. This often involves a degree of unburdening, where the counselor’s primary job is to listen, observe, and encourage sharing. In addition, the counselor will help the client to probe triggers for depressive symptoms as well as thought patterns and behaviors associated with psychological distress. Through so doing, they will attempt to identify targets for therapy and action. Sessions 2-3 often focus on generating potential solutions and choosing one or several for the participant to try. Between sessions, participants will be given so-called “homework,” often involving small, manageable assignments. For instance, if a prominent issue is that the participant is feeling lonely or isolated, the counselor might encourage them to think through possible family, friend, or religious sources of support; for instance, to identify 5 such possibilities, reach out to those individuals to make a time to meet, and meet with them. Sessions 4 and 5 tend to focus on evaluation of attempted solutions and their impacts on maladaptive thoughts, behaviors, and signs/symptoms of depression. Homework assignments may be modified. Session 6 is often devoted to integration in two ways. First, our pilot experience has shown that isolation/shame/relationship challenges often factor heavily into ante- and postnatal depression in Pujehun District. For adolescents, this could mean they

have dropped out of school, for instance, or been shunned by their families for becoming pregnant. The counselors will work with participants on re-integrating into their community to prevent the isolation that may have contributed to their depression. Second, counselors and participants will work on integrating the learned solutions to avoid/terminate unproductive thoughts and behaviors into the participant's repertoire of tools to continue treatment and prevent recurrence. The pilot program also identified group sessions as helpful for individuals who wish to engage in them. Therefore, all participants randomized to CBT will be offered the chance to engage in group sessions rather than exclusively in 1-on-1 sessions. The control group will receive no treatment. It is anticipated that a manuscript detailing CBT program development will be published.

6.1.2 DOSING AND ADMINISTRATION

The amounts of DHA, EPA, and choline (which compose the difference between intervention and control foods) are described in **section 6.1**. Dose justification is provided in **section 4.3**. Participants will begin receiving the supplementary foods on the day of enrollment/randomization and continue until the date of delivery. Participants will receive sufficient supply for 1 packet per day (100g, 5040 Kcal). Participants will be given a 2-week supply at each bi-weekly antenatal visit. Participants will be encouraged to consume their supplementary food every day. Participants will be instructed that the food requires no preparation or modification prior to consumption directly from the sachet, though they may mix it into a porridge, for instance. If a participant misses a study visit, they will not be able to receive their supplementary food for the following 2 weeks.

The earliest anticipated enrollments will occur at 10 weeks' gestation. Thus, we estimate the longest duration of supplementary feeding to be 30-32 weeks. We estimate the shortest duration of supplementary feeding to be 2 weeks, as GA is not a criteria for trial enrollment and early preterm birth is not uncommon in this population.

All participants will receive 400mg of albendazole on or after 13 weeks' gestation, 1g of azithromycin during the second and third trimesters (or only third, if enrolled during the third trimester), and 1,500 mg/75 mg of sulfadoxine/pyrimethamine (SP) every 4 weeks beginning at 13 weeks' gestation. Dose justifications are provided in Section 4.3. Each of these medicines will be given under direct observation at antenatal clinic.

Most CBT sessions will take place individually between study therapists and participants. In some cases, participants may wish to engage in group CBT sessions and, if possible, therapists will then work to set up group sessions for those interested.

6.2 PREPARATION/HANDLING/STORAGE/ACCOUNTABILITY

6.2.1 ACQUISITION AND ACCOUNTABILITY

M-RUSF+ and M-RUSF will be produced at the Project Peanut Butter (PPB) factory in Freetown, Sierra Leone. PPB is an NGO that has been operational in Sierra Leone since 2007. Mark Manary, the trial PI, is the founder and CEO of PPB. The study foods will undergo microbial and toxin testing as per UNICEF specifications. Boxes of study food will be delivered to the research teams in Pujehun and Kono Districts

as needed. Chain of custody will be documented with signatures. Study foods will be disbursed to participants at antenatal clinic by dedicated research staff, who will document receipt including colored sticker signifying study group. Participants will be instructed to return all empty sachets the following clinic visit as a loose measure of adherence and to prevent litter from accumulating in study villages.

Albendazole, azithromycin, and SP will be obtained from registered pharmacies in Freetown, Sierra Leone. Insecticide-treated bednets will also be purchased in Freetown. Safe birth kits will be distributed by the study which include sterile tools to cut/clamp the umbilical cord and clean sheets to dry and wrap the newborn.

6.2.2 FORMULATION, APPEARANCE, PACKAGING, AND LABELING

M-RUSF+ and M-RUSF formulations are detailed in **section 6.1.1**. Both foods will appear as light brown peanut-based pastes. Most ingredients have been used to produce therapeutic and supplementary foods at the PPB factory for nearly 10 years. The new ingredients are DHA, EPA and choline.

DHA/EPA have been added to therapeutic foods at PPB factories in Sierra Leone and Malawi since 2022. Our group performed formal testing of final product DHA content under various conditions and published this work in 2023.[32] In accordance with these findings, the fish oil will be added toward the end of the production process to reduce degradation/oxidation. Samples of the M-RUSF+ will be tested for DHA content quarterly during the trial to assess levels in the final product.

Choline is added to a variety of food products used worldwide. In order to maximize incorporation into the paste, it will be added at the first mixing step.

M-RUSF+ and M-RUSF will be packaged in identical foil sachets, aside from the presence of a sticker with a color indicating study group. The foods will be produced by PPB in Freetown, Sierra Leone.

Albendazole, azithromycin, and SP will be purchased locally in Freetown, Sierra Leone at registered pharmacies. There will likely be several manufacturers.

6.2.3 PRODUCT STORAGE AND STABILITY

M-RUSF+ and M-RUSF will be packaged in vacuum-sealed metallized polyester sachets with a nitrogen flush that are also used to package RUTF to meet UNICEF specifications. These packets will be placed into cardboard boxes which will be stored in a cool, dark, dry storage room at the PPB factory in Freetown and kept at < 25°C. Extrapolating from similar products (RUTF, RUSF) produced by PPB using identical packaging, shelf-life in foil packets will exceed 2 years. Once opened, the contents are recommended to be consumed within 24 hours. Study foods will be disbursed within 6 months of production.

DHA content testing will be done on stored products to assess stability until the stability profile can be determined.

Albendazole, azithromycin, and SP will be kept in original bottles in dry storage rooms at ambient temperature at the research team headquarters in Kono and Pujehun Districts. All medications will be given within 6 months of purchase and preceding indicated expiration dates.

6.2.4 PREPARATION

M-RUSF+ and M-RUSF will be ready for consumption when disbursed, requiring no further preparation or manipulation by study staff or participants. Participants will be told they are allowed to mix the study food into a porridge if that is their preference.

6.3 MEASURES TO MINIMIZE BIAS: RANDOMIZATION AND BLINDING

Randomization

Participants will be randomized individually in a 1:1 ratio to M-RUSF+ vs. M-RUSF; among these participants, those with aPHQ-9 ≥ 9 will be individually randomized in a 1:1 ratio to CBT vs. no CBT. A research team member will prepare the method of randomization for M-RUSF+ vs. M-RUSF as follows. There will be 6 colored stickers, 3 of which will indicate each study group. The stickers will adhere to a piece of paper that will allow for easy removal and re-application. The colors will be black, white, blue, yellow, green, and red. A research team member will collect 4 of each colored sticker (24 total) and 24 small opaque envelopes. They will place one sticker into each envelope, seal the envelopes, and place all 24 small envelopes into a larger opaque manilla envelope. 75 such larger envelopes will be prepared prior to study initiation and will be used one at a time. At the time of randomization, a participant will blindly reach in the larger opaque manilla envelope and withdraw one smaller opaque envelope, open it, and thereby choose their study group. The sticker will then be placed on their study card at two locations.

The method of randomization for CBT vs. no CBT will be similar, with 6 separate symbols, 3 to each group. 10 larger envelopes will be prepared in advance of the trial. After a participant randomizes themselves by choosing a symbol, this symbol will be stuck to their CBT visit card. A team member unblinded to the allocation code for CBT vs. no CBT will then instruct the participant on whether they are to receive CBT and, if so, the schedule for their sessions.

Blinding

<u>M-RUSF + vs. M-RUSF</u>

M-RUSF+ and M-RUSF will be packaged in identical foil sachets aside from a colored sticker, which will indicate study group. The two foods are identical in appearance and similar in texture and taste.

A staff member in the US responsible for scheduling M-RUSF+ and M-RUSF production at the PPB factory and scheduling delivery of boxes of food to research staff will be unblinded as to the color-group code. Staff members at the PPB factory responsible for food production and labeling the sachets will also be unblinded. None of these staff members will take part in the study otherwise, including interaction with participants, clinic visits, or outcomes assessments. They will keep the code locked.

All research clinic staff/outcomes assessors will remain blinded to M-RUSF+ vs. M-RUSF allocation throughout the trial. Staff responsible for disbursing study foods will do so by matching the color to which a participant was randomized (which will be attached to study cards and a master enrollment list)

to the color on the sachets and boxes containing sachets but will not know the underlying allocation code. Three colors will be used per group to minimize the chance of accidental unblinding, e.g., if M-RUSF+ leads to greater levels of burping or constipation, a staff member may be able to link such symptoms to a color but will have more trouble identifying group membership of other colors. Following delivery (first postnatal visit and onward), new study cards will be used ("post-partum cards") for all participants which will not contain information about their randomization group. Thus, randomized group membership will not be displayed from delivery until MDAT testing (end of trial).

Neurodevelopmental assessors will be blinded to group allocation, as the colors to which participants were randomized will not be present on postnatal visit cards.

Staff responsible for database management and data analysis will remain blinded to M-RUSF+ vs. M-RUSF allocation, as randomized group colors will be used in the database and the code linking color to food will remain locked until all analyses have been completed.

<u>CBT vs. no CBT</u>

Neither participants nor therapists providing CBT to an individual participant will be blinded to CBT vs. no CBT allocation. Upon randomization, a sticker indicating group assignment will be placed inside a folded appointment card behind an opaque stapled piece of paper, on the enrollment aPHQ-9 assessment, and on a master enrollment sheet. All counselors will know the code linking randomization symbol to CBT vs. no CBT so they can determine which participants should receive CBT vs. not. The counselor who performed the screening/enrollment aPHQ-9 will link the randomized sticker to CBT vs. not, notify the participant whether they have been randomized to receive CBT, and schedule their first therapy visit with them if so. This counselor will not perform the outcome aPHQ-9 assessment for the individual being randomized. Rather, on the front of the participant's CBT study appointment card, they will write the name of a different counselor who is to obtain subsequent aPHQ-9 assessments, including the outcome (week 8) aPHQ-9 assessment. In this manner, the outcomes assessor may not know participant allocation. They will be instructed not to open the appointment card. In an effort to avoid accidental unblinding, the allocation group sticker will have been placed behind an opaque piece of paper stapled into the card, which outcome-assessor counselors will also be instructed not to fold up and thereby unblind participant allocation. They will be instructed not to ask the participant whether they have received CBT. Despite these measures, it is possible that unblinding of the primary outcome aPHQ-9 assessment may occur. For example, the outcome assessor may see the participant at clinic undergoing CBT, or the participant may reveal they received CBT while the assessor is performing the aPHQ-9.

Neurodevelopmental assessors for offspring will be blinded to CBT vs. no CBT, as the symbols to which participants were randomized will not be present on visit cards.

6.4 STUDY INTERVENTION COMPLIANCE

This trial has been designed to approximate practices that might happen were its results to be applied in resource-constrained settings with high rates of undernutrition in pregnancy. That is, undernourished pregnant women will be provided with food and encouraged to consume it, but this consumption will not be directly monitored to confirm receipt. Adherence to M-RUSF+ vs. M-RUSF will be assessed in several ways, however. First, participants will be asked to bring empty sachets back to clinic fortnightly,

and these will be identified by group color and be counted. Second, we will enlist community health workers stationed at each antenatal clinic to visit participants at their homes at random (schedule made using a random number generator) approximately q6 weeks to document the colored sticker on a participant's sachets and the number of full sachets remaining on date of visit, which will then be compared to expectations based on their last study visit. Third, blood spots will be collected from participants at baseline and between 32-36 weeks' gestation for RBC-PUFA status, which will provide a sense of overall adherence.

Adherence to albendazole, azithromycin, and SP will be known because each medication will be given under direct observation at antenatal clinic.

Adherence to CBT will be known by documentation of visits by therapists.

6.5 CONCOMITANT THERAPY

For this protocol, a prescription medication is defined as a medication that can be prescribed only by a properly authorized/licensed clinician. Medications to be reported on participant data collection forms on a bi-weekly basis are concomitant prescription medications, over-the-counter medications, and supplements.

Receipt of albendazole, azithromycin, and SP will be documented on study cards.

6.5.1 RESCUE MEDICINE

Not applicable.

7 STUDY INTERVENTION DISCONTINUATION AND PARTICIPANT DISCONTINUATION/WITHDRAWAL

7.1 DISCONTINUATION OF STUDY INTERVENTION

Discontinuation from supplementary food receipt or CBT does not mean discontinuation from the study, and remaining study procedures should be completed as indicated by the study protocol. If a clinically significant finding is identified after enrollment, the first priority will be clinical management of the participant, including a triage decision. This decision will be made by the most clinically experienced staff member present, often a nurse-midwife. The research associate will notify the principal investigator within 3 days to determine if any change in study management is needed. Any new clinically relevant finding will be reported as an adverse event (AE). The two foreseeable reasons for intervention discontinuation are participant choice and allergy to M-RUSF+ / M-RUSF. The data to be collected at the time of study intervention discontinuation will include the following:

- Reason for discontinuation
- Maternal anthropometry
- Physical examination and symptom evaluation (e.g., rash, abdominal discomfort, trouble breathing)
- Need for hospitalization
- Outcome of hospitalization, if needed

Participants who discontinue the intervention will be encouraged to remain in the study for follow-up. If they agree, they will undergo the same data collection as those who remain on the intervention.

In the case of participants who have been diagnosed with depression (whether randomized to CBT or not), the same procedures will be undertaken as above. In the event of active suicidal or homicidal ideation, the participant will be offered transport to the Pujehun District Hospital and the counselor's clinical supervisor will be informed to advise and direct care. The research associate will notify the principal investigator within 3 days.

7.2 PARTICIPANT DISCONTINUATION/WITHDRAWAL FROM THE STUDY

Participants are free to withdraw from participation in the study at any time upon request. An investigator may discontinue or withdraw a participant from the study for the following reasons:

- If any clinical adverse event (AE), laboratory abnormality, or other medical condition or situation occurs such that continued participation in the study would not be in the best interest of the participant
- If the participant meets an exclusion criterion (either newly developed or not previously recognized) that precludes further study participation
- Participant defaults, i.e. misses 3 consecutive study visits

The reason for participant discontinuation or withdrawal from the study will be recorded on the participant data card. Individuals who sign the informed consent form, are randomized and receive the

study intervention, and subsequently withdraw, or are withdrawn or discontinued from the study, will not be replaced.

7.3 LOST TO FOLLOW-UP

A participant will be considered lost to follow-up (defaulted) if they fail to return for 3 consecutive study visits and are unable to be contacted by the study site staff.

The following actions will be taken if a participant fails to return to the clinic for a required study visit:

- Staff will attempt to contact the participant via telephone, send a message with their next study visit date via a community health worker (CHW), ask whether the woman remains pregnant, reschedule the missed visit, counsel the participant on the importance of maintaining the assigned visit schedule, and ascertain if the participant wishes to and/or should continue in the study. CHWs will receive a small gift for any successful participant tracing or information obtained via their visit.
- Before a participant is deemed lost to follow-up, the investigator or designee will make efforts to regain contact with the participant: at least 3 telephone calls, asking a CHW to visit the participant at their home 2 times, and taking a trip with the research team to the participant's documented address after 3rd consecutive missed visit. These contact attempts will be documented in the participant's study file.

If a participant is not able to be tracked and misses three consecutive visits, but then returns to study clinic thereafter and wishes the rejoin, they will be re-engaged and their data will be collected as was originally intended to the most accurate degree possible (e.g., if they were lost to follow-up during the antenatal period, and then present to clinic when the child is 6mo of age, e.g., date of birth / birth weight / birth length may not be known/identifiable; note will be made of uncertainty where relevant, and the period during which they were lost to follow-up will be documented). The overarching goal will be to collect as much data as possible for intention-to-treat analysis, and document uncertainty and note missing data where relevant.

8 STUDY ASSESSMENTS AND PROCEDURES

8.1 EFFICACY ASSESSMENTS

Screening: M-RUSF+ vs. M-RUSF

There will be two settings for screening. The first, which will afford partial screening/sensitization, will be done by antenatal clinic nurses and community health workers (CHWs). The study team will disburse mid-upper arm circumference (MUAC) tapes at each participating antenatal clinic for use at clinic. Nurses and CHWs at each clinic will be informed to offer screening with MUAC measurement to any woman who thinks she may be pregnant. All women who think they are pregnant, regardless of MUAC, will be recommended to attend the next antenatal clinic day. If a woman is found to have MUAC ≤ 23.0 cm, they will be informed of the trial and possibility of their enrollment pending further evaluation. If possible, antenatal clinic staff may also pursue BMI measurements, in which case BMI < 18.5 would trigger the same discussion. All women who think they may be pregnant will be encouraged to visit antenatal clinic. Those with low MUAC will be informed of the trial and possibility for enrollment if all criteria are met.

Screening for trial enrollment will take place at bi-weekly antenatal clinic visits by research staff, primarily Sierra Leonean nurses, a full-time research associate, and US-trained MDs when they are present in Sierra Leone. Women who think they may be pregnant will be asked whether they would like to be screened for possible trial enrollment, a process which will involve anthropometry, brief history questions, fundal height measurement, and ultrasonography for estimated gestational age. Women who consent will first be asked their age. If they are < 13 years of age, they will not be eligible for the trial. Next, MUAC and BMI measurements will be done by research staff. If potential participants meet eligibility criteria by MUAC or BMI, they will undergo fundal height measurement. If there is ambiguity about whether the participant is pregnant on the basis of LMP report and physical examination, urine pregnancy testing will be done. On the basis of prior trial experience in this population, this situation is expected to be rare. If the participant is confirmed positive, they will proceed to ultrasound assessment of gestation age (as detailed elsewhere). All ultrasonography will be done by research staff who have received training in use of gestational age estimation software (as detailed elsewhere).

There may be instances where the fundal height is palpable and participant reports they are pregnant, which later it is determined the participant is not pregnant. This will be considered a randomization failure (randomization of a participant who did not meet inclusion criteria, because they were not pregnant). This is not the same as miscarriage or stillbirth, which will not qualify as randomization failures, but will instead be documented and reported.

Following successful GA estimation, exclusion criteria will be assessed: involvement in a separate supplementary feeding program, allergy to intervention components, history of gestational diabetes, blood pressure measurement, and general clinical assessment for pregnancy complications requiring immediate hospitalization, such as signs and symptoms of pre-eclampsia or severe anemia. If their blood pressure is > 140/90 on 2 measurements >1 hour apart or >160/110 on any one measurement, they will not be eligible for enrollment that day and will be referred for further care. If they have a clinical indication for immediate hospitalization, they will not be eligible for enrollment at that visit and will be offered transport to Pujehun District Hospital accompanied by our research nurses and field staff. If such a participant's blood pressure is controlled on subsequent visits, they will be eligible for re-screening.

If they meet all study inclusion/exclusion criteria, full discussion of trial procedures and formal consent process will be done. For individuals who are illiterate or indicate they cannot follow the consent process (e.g., due to lack of schooling), an impartial witness will witness the consent process and also sign the informed consent document. If the woman consents (or, if <18 years of age and unmarried, assents with caregiver/guardian consent), they will then undergo randomization, as described elsewhere. Women who meet enrollment criteria and consent to participation will be enrolled, with interventions initiated, that same day. This includes study food and medications with timing detailed elsewhere in this protocol.

It is likely that participants pregnant with more than a singleton, e.g. twins, will be included in the study. Staff and participant may not know about presence of twins until birth. Data on these participants will be collected and reported, but will not be included in the main outcome comparisons.

If the woman does not meet criteria by MUAC or BMI, they will be encouraged to continue attending antenatal clinic and may be screened on subsequent clinic days; if they meet criteria in the future, the above procedures will be done. Similarly, if a woman is < 13 years of age, she will be encouraged to continue attending antenatal clinic and may be screened on subsequent clinic days. Women who are referred for hospitalization may also return to antenatal clinic after recovery for repeat screening.

Screening: CBT vs. no CBT

All participants in the M-RUSF+ vs. M-RUSF comparison will undergo aPHQ-9 testing monthly throughout their pregnancy and at each postnatal study visit. At the subset of clinics chosen for CBT study enrollment, if their aPHQ-9 ≥ 9 at any time, they will be offered enrollment into the CBT vs. no CBT comparison until adequate sample size has been achieved for this sub-study; once adequate sample size has been achieved, enrollment into CBT vs. no CBT comparison will be halted. If they meet all inclusion/exclusion criteria and consent (or assent + parental/guardian consent if <18 yo and unmarried) to participation, they will then be randomized. CBT will start within the week following randomization.

Efficacy assessments

- Gestational duration: this will be determined by adding the estimated gestational age (GA, days) at enrollment to the time (days) between enrollment and date of delivery. Enrollment GA estimation ultrasonography will be done by trained research staff nurses using Butterfly probes and the Butterfly Gestational Age Tool, which uses an algorithm (not commercially available as of this writing) imbedded within the Butterfly application. This program was developed by The Fetal Age Machine Learning Initiative (FAMLI), an ongoing project chaired by Jeffrey Stringer, MD, of the University of North Carolina. The algorithm was validated in a large Zambian cohort, including when used by untrained sonographers performing blind transabdominal sweeps.[33] GA estimation ultrasound protocol is detailed in an SOP elsewhere. Briefly, first, fundal height will be measured and entered into the program to determine the number of lateral sweeps necessary. The Tool will then guide the user to conduct cranio-caudal and lateral sweeps. Each sweep is expected to last 10 seconds; if the sweep is done too quickly or slowly, the program automatically prompts the user to re-do the sweep. For each sweep, the app directs the user on location and direction, and counts down from 10 seconds. First, 5 cranio-caudal sweeps will be done. The first starts at the midline at the pubis and ends at the xyphoid. Next, 2 cranio-caudal sweeps are done to the left of midline, and then 2 to the right, with each sweep overlapping ~50% which its nearest neighbor. The Tool will then guide the user to perform lateral sweeps, beginning just above the pubic symphysis, collecting from the left lateral edge of the uterus to the right lateral edge of the uterus and repeating sweeps superiorly with 1 probe's distance

between. The user may be guided to conduct 3-4 sweeps depending on fundal height. After the last sweep is complete, the user selects “Calculate” on the Tool. If the sweeps were sufficient, the Tool will calculate an estimated GA, which the user then saves and transcribes to the participant’s data card. Once staff can access WiFi, the studies (video loops of each sweep) and results will be uploaded to a secure cloud-based database. Every ultrasound video collected will be uploaded to a secure storage cloud within a week of collection. The GA tool development did not include sufficient participants to estimate GA < 9 weeks. For this reason, only participants with palpable fundal height will undergo US GA estimation, and only estimates > 9 weeks will be used for pregnancy dating. If US estimates are not available, fundal height will be used as a proxy for GA at enrollment. How the GA was estimated will be documented and reported.

- Malawi Developmental Assessment Tool: testing will take place according to the SOP detailed elsewhere. Briefly, neurocdevelopmental assessors will undergo training prior to trial initiation. At the 9mo postnatal visit, these neurodevelopmental assessors will assess gross motor, fine motor, and language development via direct testing of the infant, and social development via caregiver interview, across 136 items total. Each item is scored as pass, fail, or NA. NA is used when a child does not engage sufficiently to allow a determination. As per testing recommendations, six consecutive fail determinations will trigger an automatic jump to the next domain of testing. This testing will be done by trained neurodevelopmental assessors in the participant’s native language. Data will be collected on tablets with secure storage cloud upload. A program developed for the MDAT automatically calculates age-adjusted z-scores globally and for each sub-domain.
- aPHQ-9: Adaptation of the standard 9-item questionnaire will be used wherein respondents are asked to estimate the number of days in the preceding 2 weeks that they have experienced each item. Scores range from 0-27, with up to 3 points per item. Points correspond to symptom/sign frequency, with 0 = not at all, 1 = several days, 2 = more than half the days, and 3 = nearly every day. aPHQ-9 testing will be done by research counselors in participant native language and with pictorial guides that have been developed and successfully piloted for use in Pujehun District, Sierra Leone, by the trial PI. Scores will be recorded on participant data sheets.
    - The PHQ-9 was lightly adapted based on pilot usage in Pujehun and for contextual and cultural factors by Mental Health Coalition of Sierra Leone collaborators Abdulai Jawo Bah, MPCPharm, Joshua Abioseh Duncan, and Isata Timsah-Delaney. Several of these adaptations were made during Abdulai’s PhD research on tools for assessing psychological distress in Sierra Leone (publication pending). The adaptations are as follows:
        - Q5: “Poor appetite or overeating” -> “Poor appetite”
        - Q7: “Trouble concentrating on things, such as reading the newspaper or watching television” -> “Trouble concentrating on things, such as doing household chores”
    - In addition, through testing of various visual representations of sign/symptom frequency on PHQ-9, it was determined that the use of empty ((0 = Not at all), a third full (1 = several days), two thirds full (2 = More than half the days), and full (3 = Nearly every day) jerry cans should be added to the questionnaire to aid mothers, particularly illiterate mothers, in determining how to answer each questions. Finally, the collaborators also translated the PHQ-9.
- aPHQ-9 ≥ 9: defines depression in this study
- aPHQ-9 < 5: common cut-off used to deem someone with no depression (even mild)
- Maternal MUAC: MUAC will be measured in duplicate by 2 study staff to the nearest 1mm with a standard insertion measuring tape (TALC, St. Albans, UK), according to standard procedures.

- Maternal weight: weight will be measured in duplicate with a Seca 803 digital scale to the nearest 100g.
- Maternal height: height will be measured in duplicate with a Seca 213 stadiometer to the nearest 1mm, at enrollment visit only.
- Fundal height: fundal height will be measured in duplicate to the nearest 5mm using a non-elastic measuring tape.
- Blood spot: a blood spot will be taken at enrollment and at gestational age 32-36 weeks in selected subjects according to SOP detailed elsewhere.
- Cord blood: a cord blood spot sample will be collected from participants at selected centers as possible, according to SOP detailed elsewhere.
- Date of birth (as it affects other outcome assessments): a birth assessment team will receive notification of birth from participant or designee via phone call. They will travel to meet the participant and offspring as soon as possible to record date and time of birth, when possible.
- Placental weight: the placenta will be weighed from participants at selected centers as possible, according to SOP detailed elsewhere.
- Intrauterine growth: algorithms developed by UNC collaborators will be used for assessing intrauterine growth on serial ultrasounds
- Placental histology: inspection of changes in e.g. vascularity in subset of participants
- Weight for GA, SGA: US-estimated gestational age and birth weight will be used to assess weight-for-gestational-age. SGA is defined as weight-for-GA < 10th percentile.
- Offspring vital status. If found to be deceased, a standardized Verbal Autopsy Tool will be used. Date / time of death will be recorded.
- Hospitalization for participant or offspring: date, reason, outcome
- Infant MUAC: measured in duplicate by 2 study staff to the nearest 1mm with a standard insertion tape
- Infant weight: measured in duplicate using a Seca 334 digital scale
- Infant length: measured in triplicate to the nearest 2mm using a rigid length board
- Infant chest circumference: measured in duplicate to the nearest 1mm with a standard insertion tape
- Infant head circumference: measured in duplicate to the nearest 1mm with a standard insertion tape
- Delivery method

All maternal and infant anthropometry will be performed by trained research nurses, the full-time research associate, or US-trained MDs. Birth assessments will be done by a team designated to respond to notification of birth as soon as possible.

Participants will be notified of their anthropometric measurements, blood pressure, estimated gestational age, and fundal height measurements.

Administration of trial interventions

Participants will be provided with 2 weeks of their randomized study food prior to leaving clinic on their day of enrollment. They will be instructed on opening and administration of the study food, with particular attention given to avoiding sharing of the food amongst others. They will be counseled that this food has been designed to help them and their baby. On appropriate clinic days, they will be given albendazole, azithromycin, and/or SP under direct observation by research staff nurses. Timing of these is described elsewhere.

In addition to trial interventions, all participants will receive standard antenatal care as per Sierra Leone national guidelines. This care will be provided by Sierra Leone district medical staff. They will also receive safe birth kits.

8.2 SAFETY AND OTHER ASSESSMENTS

Safety Assessments

- Participants will be asked about bleeding at each bi-weekly antenatal visit
- Participants will be asked about diarrhea, fever, rash, cough in the 2 weeks preceding each visit, at each visit
- Maternal death
- Maternal antenatal hospitalization not for childbirth
- Post-term delivery (≥ 42 weeks gestation)
- Peripartum complication requiring transfer to facility with higher level of care
- Miscarriage
- Stillbirth
- Neonatal death
- Infant death
- Offspring diarrhea in preceding 2 weeks

Additional Assessments

- Demography: maternal date of birth, age, village of residence, level of education, # of children in household, # adults in household, father of coming child live with mother, # people sleep in same room as mother last night, household roof material, # radios/bicycles in home, house with electricity, cellphone in home, household water source, # times per day collecting water, use of pit latrine, animals sleep in home last night
- Adherence: participants will be asked whether they finished their RUSF
- Attendance at clinic: dates of attendance will be collected
- Attendance at CBT visits: among participants randomized to receive CBT, attendance will be tracked
- Receipt / date of receipt of study interventions: azithromycin, albendazole, bednet, malaria chemoprophylaxis
- Health history: illness in last 2 months (none, diarrhea, pneumonia, TB, malaria), reduction in food intake during past 6 months, mother on TB treatment, other person in house on TB treatment, sleep beneath bed net, chew/sniff tobacco, kola nut use, alcohol consumption, pregnancy history (#, # live births, age of youngest child, # miscarriages, # stillbirths), currently breastfeeding, currently taking supplements, HIV test/status
- Household food insecurity access questionnaire
- Participant consumption of fish or egg
- Blood pressure: BP will be measured using a digital BP cuff at each antenatal visit.
- Last menstrual period (as estimated by mother)
- Location of birth
- Date and time of birth visit: recorded by birth measurement team
- Number of offspring, e.g. singleton, twins

- Offspring time to breast
- Offspring breastfeeding status
- Offspring received other food / liquid besides breastmilk
- Offspring eaten egg or fish
- Offspring vaccination status
- Inter- and intra-rater reliability assessments of MDAT, aPHQ-9

8.3 ADVERSE EVENTS AND SERIOUS ADVERSE EVENTS

8.3.1 DEFINITION OF ADVERSE EVENTS (AE)

As adopted from 21 CFR 312.32 (a):

An adverse event means any untoward medical occurrence associated with the use of an intervention in humans, whether or not considered intervention-related.

In the context of this trial, miscarriages and stillbirths will not be considered adverse events. Complications of either would be considered AE or SAEs. Both will be tracked and recorded throughout the trial. A previous study in the same location and study population identified approximately 4% combined rate of stillbirth and miscarriage.[6] Rates in the present trial will be compared quarterly to this expectation by the PI and independent medical monitor. If there is imbalance or higher rates than expected, SLESRC, PBSL, and IRB will be notified and will determine how to proceed.

Hospitalization for events that are expected are also not considered SAEs, including: routine treatment or monitoring of miscarriage or threatened preterm birth, not associated with deterioration in condition; elective or pre-planned treatment for a pre-existing condition that was unrelated to the indication under study and did not worsen, including elective caesarean section; hyperemesis that quickly resolved.

8.3.2 DEFINITION OF SERIOUS ADVERSE EVENTS (SAE)

As adopted from 21 CFR 312.32 (a):

An adverse event or suspected adverse reaction is considered "serious" if, in the view of the investigator, it results in any of the following outcomes: death, a life-threatening adverse event, inpatient hospitalization or prolongation of existing hospitalization, a persistent or significant incapacity or substantial disruption of the ability to conduct normal life functions, or a congenital anomaly/birth defect. Important medical events that may not result in death, be life-threatening, or require hospitalization may be considered serious when, based upon appropriate medical judgment, they may jeopardize the participant and may require medical or surgical intervention to prevent one of the outcomes listed in this definition.

8.3.3 CLASSIFICATION OF AN ADVERSE EVENT

8.3.3.1 SEVERITY OF EVENT

For adverse events, the following guidelines will be used to describe severity.

- **Mild** – Events require minimal or no treatment and do not interfere with the participant's daily activities.
- **Moderate** – Events result in a low level of inconvenience or concern with the therapeutic measures. Moderate events may cause some interference with functioning.
- **Severe** – Events interrupt a participant's usual daily activity and may require systemic drug therapy or other treatment. Severe events are usually potentially life-threatening or incapacitating. Of note, the term "severe" does not necessarily equate to "serious".

8.3.3.2 RELATIONSHIP TO STUDY INTERVENTION

On the basis of prior trial data as well widespread use of antenatal fish oil supplementation and presence of comparable amounts of choline in widely consumed foods, there are no anticipated serious adverse events associated with addition of fish oil/choline to the supplementary food. Similarly, while CBT can lead to short-term distress during therapy, serious adverse events are also not anticipated for this technique that is widely used for management of ante- and postnatal depression. In a pilot program ongoing in Sierra Leone that has included >500 women with ante- or postnatal depression, there have been no cases of SI or HI that developed during CBT. In addition, and in line with prior clinical trials of antenatal fish oil supplementation, while non-serious adverse events typically associated with pregnancy will be monitored, recorded, and reported at the quarterly data safety monitoring meetings, they will not be actively assessed for relation to study intervention.[25]

As detailed below in several sections, on a monthly basis, the study monitor will compile all adverse events by study group. If there is concern for imbalance (especially if statistically significant), they will inform the principal investigator and independent medical reviewer (IMR) within 3 days. The IMR and PI will then review the unblinded aggregate data to make a determination about potential relatedness to the intervention using the categories below. They will then notify SLESRC, the Pharmacy Board of Sierra Leone (PBSL), and the Wash U IRB within 24 hours. The principal investigator will then make a decision as to whether the trial can proceed.

- **Definitely Related** – There is clear evidence to suggest a causal relationship, and other possible contributing factors can be ruled out. The clinical event occurs in a plausible time relationship to study intervention administration and cannot be explained by concurrent disease or other drugs or chemicals. The response to withdrawal of the study intervention should be clinically plausible. The event must be pharmacologically or phenomenologically definitive, with use of a satisfactory rechallenge procedure if necessary.
- **Probably Related** – There is evidence to suggest a causal relationship, and the influence of other factors is unlikely. The clinical event occurs within a reasonable time after administration of the study intervention, is unlikely to be attributed to concurrent disease or other drugs or chemicals, and follows a clinically reasonable response on withdrawal. Rechallenge information is not required to fulfill this definition.
- **Potentially Related** – There is some evidence to suggest a causal relationship. However, other factors may have contributed to the event (e.g., the participant's clinical condition, other concomitant events). Although an AE may rate only as "possibly related" soon after discovery, it

can be flagged as requiring more information and later be upgraded to “probably related” or “definitely related”, as appropriate.

- **Unlikely to be related** – A clinical event whose temporal relationship to study intervention administration makes a causal relationship improbable (e.g., the event did not occur within a reasonable time after administration of the study intervention) and in which other drugs or chemicals or underlying disease provides plausible explanations (e.g., the participant’s clinical condition, other concomitant treatments).
- **Not Related** – The AE is completely independent of study intervention administration, and/or evidence exists that the event is definitely related to another etiology. There must be an alternative, definitive etiology documented by the clinician.

8.3.3.3 EXPECTEDNESS

There will be two mechanisms of expectedness assessment, including both trial PI Mark Manary and the Independent Medical Reviewer (IMR). AEs and SAEs will be considered unexpected if the nature, severity, or frequency of the event is not consistent with the risk information previously described for the study intervention. Unexpected AEs and SAEs will be reported to SLESRC, PBSL, and WUSTL IRB.

Expected AEs:

- Based on prior trial experience involving undernourished pregnant women in rural Sierra Leone, several AEs are expected to occur during the trial:
    - Nausea
    - Diarrhea
    - Vomiting
    - Rash
    - Fever
    - Vaginal bleeding
    - Headache
    - Malaria
    - Heartburn/acid reflux
    - Decreased fetal movement
    - Mastitis

Expected SAEs

- Based on prior trial experience involving undernourished pregnant women in rural Sierra leone, several SAEs are expected to occur during the trial
    - Maternal death (<1%)[6]
    - Neonatal death (2-5%)[6]
    - Infant death (6-9%)[6]
    - Pre-eclampsia, hospitalization for
    - Severe hypertension, hospitalization for
    - Malaria, hospitalization for
    - Offspring seizures

8.3.4 TIME PERIOD AND FREQUENCY FOR EVENT ASSESSMENT AND FOLLOW-UP

The occurrence of an AE or SAE may come to the attention of study personnel during study visits (bi-weekly antenatal and 5 postnatal visits), either solicited or unsolicited, or via report from antenatal clinic staff regarding events that occurred between study visits.

All AEs including local and systemic reactions not meeting the criteria for SAEs will be captured on the participant's data card. All AEs not typically associated with pregnancy and not expected (see Section 8.3.3.3) in the clinician's judgement will undergo further assessment. This can include symptoms, signs, severity, duration, or other aspects of the AE not typically associated with pregnancy. Information to be collected for these AEs includes event description, duration, clinician's assessment of severity, possible relation to study product (food, medication), and time of resolution/stabilization of the event. All AEs occurring while on study must be documented appropriately regardless of relationship. All AEs will be followed to adequate resolution.

Any medical condition that is present at the time that the participant is screened will be considered as baseline and not reported as an AE. However, if the study participant's condition deteriorates at any time during the study, it will be recorded as an AE.

Changes in the severity of an AE will be documented to allow an assessment of the duration of the event at each level of severity to be performed. AEs characterized as intermittent require documentation of onset and duration of each episode.

All reportable events will be recorded with start dates (may be approximate) occurring any time after informed consent is obtained until the last day of study participation.

In addition to monitoring in the normal course of clinical trial operations, the IMR will be responsible for reviewing AE and SAE data yearly.

8.3.5 ADVERSE EVENT REPORTING

AEs typically associated with pregnancy that are occurring in an expected manner in the clinician's (most often, Sierra Leonean nurse-midwife) judgment will not be actively reported. If an AE typically associated with pregnancy differs in any way deemed significant by the clinician, such will be documented on the study form and discussed with at least a nurse-midwife and the research associate to determine if clinical management steps need to be taken. If the research team has concerns regarding the safety of continued intervention receipt, they will stop the intervention(s) and must then notify the study monitor, IMR, and PI of the issue and their actions within 24 hours. The study monitor and IMR will analyze data collected up to that point to determine if the AE might be occurring at greater-than-expected rates or at greater rates in one or several groups compared to others. They will report to the PI , who will then decide how to proceed in consultation with nurse-midwife team.

If any research team member suspects AEs to be occurring at rates higher than anticipated or in a/several study groups more than others, they will notify the research associate, who will notify the PI, IMR, and study monitor within 3 days. The IMR and study monitor will investigate and report to the PI, who will decide how to proceed. Otherwise, on a monthly basis, the study monitor will compile AEs by study group. If rates are imbalanced or higher than anticipated based on comparison to prior trial data, they will notify the IMR and PI within 3 days and the PI will evaluate the unblinded aggregate summary data. The PI will notify SLESRC, PBSL, and WUSTL IRB and make a determination about how to proceed.

The Independent Medical Reviewer (IMR) will receive information on all reportable adverse events (events that are unexpected and related or probably related to the interventions) within 5 working days. The IMR will independently review AEs at least yearly. The IMR will generate a report on AEs for the PI, SLESRC, PBSL, and WUSTL IRB. Through its reviews of the study, the IMR will determine whether cumulative data indicates the need to change the research design, to modify information presented to participants, or to terminate the project. Any action taken to suspend or terminate the project will be reported to the PI, SLESRC, PBSL, and WUSTL IRB. The IMR will evaluate the final study manuscripts to assure results are fairly presented and conclusions are appropriate.

8.3.6 SERIOUS ADVERSE EVENT REPORTING

In the event of an SAE (specifically, notification of SAE or staff becoming aware of SAE through other means), research staff must notify the research associate within 24 hours. The RA will attempt to gather information regarding the SAE. They will complete the SAE report form as outlined by the Pharmacy Board of Sierra Leone. They will send this report to the study monitor and PI within 24 hours, and to the PBSL within 48 hours. Monthly, the study monitor will assess rates of this SAE in total and between groups and, if imbalance or concern for greater-than-expected rates are found, they will notify the PI and IMR who will notify SLESRC, PBSL, and WUSTL IRB and make a determination about how to proceed.

If any research team member suspects SAEs to be occurring at rates higher than anticipated or in participants within a study group more than others, they will be expected to notify the research associate, who will be required to notify the study monitor and principal investigator within 24 hours and PBSL within 48 hours. The study monitor will investigate by compiling SAE rates overall and by group and report to the IMR and PI within 24 hours, who will decide how to proceed. SAE rates irrespective of group will be compared to those identified in the 2019-2021 maternal undernutrition trial that took place amongst a similar population and with similar interventions in an effort to detect higher than anticipated SAE rates.

On a monthly basis, the study monitor will compile SAEs in total and by color/group. If rates are higher (statistically) than expected (compared to 2021 study), the study monitor will notify the principal investigator and IMR within 24 hours. The PI will notify SLESRC, PBSL, and the WUSTL IRB and make a determination about how to proceed. In addition, the study monitor will compare rates of SAEs between study groups. If rates are statistically imbalanced, they will notify the IMR and PI within 24 hours and the PI will evaluate the unblinded summary data. The PI will notify SLESRC and WUSTL IRB and make a determination about how to proceed.

Follow-up reports on SAEs will be made and presented to SLESRC, PBSL, and WUSTL IRB when there is a change in the severity of SAE, when there is a new development on a previously reported SAE, or when the SAE resolved.

In the case of fatalities, if a formal autopsy is completed, this report will be saved and presented to SLESRC and PBSL. If a formal autopsy is not practicable, as is expected often to be the case based on prior experience conducting trials in similar settings, a verbal autopsy report will be completed and submitted to SLESRC, PBSL, and the WUSTL IRB. The verbal autopsy will be conduced in line with the World Health Organization guideline. If obtainable, the cause of death shall be classified according to the current ICD guideline.

The IMR will independently review SAEs at least twice yearly. The IMR may generate a report on SAEs for the PI, SLESRC, PBSL, and WUSTL IRB. Through its reviews of the study, the IMR will determine whether cumulative data indicates the need to change the research design, to modify information presented to participants, or to terminate the project. Any action taken to suspend or terminate the project will be reported to the PI, SLESRC, PBSL, and WUSTL IRB.

8.3.7 REPORTING EVENTS TO PARTICIPANTS

In the event that SAEs occur at a higher rate in the intervention group that in the control group, participants will be informed.

8.3.8 EVENTS OF SPECIAL INTEREST

Not applicable

8.3.9 REPORTING OF PREGNANCY

Pregnancy is a trial inclusion criterion.

8.4 UNANTICIPATED PROBLEMS

8.4.1 DEFINITION OF UNANTICIPATED PROBLEMS (UP)

The Office for Human Research Protections considers unanticipated problems involving risks to participants or others to include, in general, any incident, experience, or outcome that meets **<u>all</u>** of the following criteria:

- Unexpected in terms of nature, severity, or frequency given (a) the research procedures that are described in the protocol-related documents, such as the Institutional Review Board (IRB)-approved research protocol and informed consent document; and (b) the characteristics of the participant population being studied;
- Related or possibly related to participation in the research ("possibly related" means there is a reasonable possibility that the incident, experience, or outcome may have been caused by the procedures involved in the research); and
- Suggests that the research places participants or others at a greater risk of harm (including physical, psychological, economic, or social harm) than was previously known or recognized.

8.4.2 UNANTICIPATED PROBLEM REPORTING

When an unanticipated problem that is a SAE is suspected, the principal investigator must be informed within 24 hours and then must inform SLESRC, PBSL, and the WUSTL IRB within 24 hours. The UP report will include the following information:

- Protocol identifying information: protocol number, PI's name, and the IRB project number;
- A detailed description of the event, incident, experience, or outcome;
- An explanation of the basis for determining that the event, incident, experience, or outcome represents an UP;
- A description of any changes to the protocol or other corrective actions that have been taken or are proposed in response to the UP.

Any other UP will be reported to the IRB within 3 working days of the investigator becoming aware of the problem.

8.4.3 REPORTING UNANTICIPATED PROBLEMS TO PARTICIPANTS

UPs will be reported to all participants who remain enrolled. Efforts will be made to contact participants who have completed the trial to inform them of UPs.

9 STATISTICAL CONSIDERATIONS

9.1 STATISTICAL HYPOTHESES

All analyses will be compared under a superiority framework.

Primary Efficacy Endpoints:

- We hypothesize that adding 500mg DHA, 500mg EPA, and 550mg choline to a supplementary food provided to undernourished pregnant Sierra Leonean women with enrollment gestational age ≤ 30 weeks will prolong gestation (days) compared with provision of a similar supplementary food that does not contain DHA, EPA, or choline.
- We hypothesize that adding 500mg DHA, 500mg EPA, and 550mg choline to a supplementary food provided to undernourished pregnant Sierra Leonean women will improve offspring cognitive development at 9 months of age (MDAT global z-score) when compared with provision of a similar supplementary food that does not contain DHA, EPA, or choline.
- Among women enrolled in the above trial at select clinics who develop ante- or postnatal depression, we hypothesize that providing a novel cognitive behavioral therapy (CBT) will improve depression (score on adapted PHQ-9) compared with no CBT.

Secondary Efficacy Endpoints

- We hypothesize that adding 500mg DHA, 500mg EPA, and 550mg choline to a supplementary food provided to undernourished pregnant Sierra Leonean women with enrollment GA ≤ 30 weeks will:
    - Reduce early preterm birth (<34 weeks)
    - Increase birth length and weight
    - Reduce low birth weight (<2.5 kg)
    - Reduce preterm birth (<37 weeks)
    - Reduce neonatal mortality (live birth and subsequent death < 28 days from delivery)
    - Reduce SGA (<10th percentile birth weight for GA)
    - Increase placental weight
    - Increase maternal/cord blood DHA status
    - Reduce depression symptoms (aPHQ-9 scores)
    - Increase MDAT sub-domain scores
- We hypothesize that adding 500mg DHA, 500mg EPA, and 550mg choline to a supplementary food provided to undernourished pregnant Sierra Leonean women will:
    - Improve MDAT sub-domain (gross motor, fine motor, language, social) z-scores
    - Prolong gestation
    - Improve offspring birth weight, length
    - Reduce depressive symptoms (adapted PHQ-9) prior to delivery
    - Increase maternal and cord blood DHA levels
- Among women enrolled in the above trial at select clinics who develop ante- or postnatal depression, we hypothesize that providing a novel cognitive behavioral therapy (CBT) will:
    - Reduce depression, defined as aPHQ-9 ≥ 9
    - Increase proportion with more strict criterion for no depression, aPHQ-9 < 5
    - Prolong gestation
    - Improve gestational weight gain
    - Improve offspring birth weight, length

- Improve infant cognitive development

9.2 SAMPLE SIZE DETERMINATION

Trial sample size determination was done in reference to both primary outcomes, duration of gestation in days and MDAT global-z-score. The goal was to achieve at least 80% power for both primary outcomes. A simulation-based power analysis approach was undertaken. All analyses were done using a two-sided α of 0.05.

Gestational Duration

The population for analysis for this outcome variable will be women with enrollment GA ≤ 30 weeks.

A preliminary goal of powering that study to detect a difference gestational duration of 3 days was made based on a secondary analysis of the 2021 MamaDutasi trial[6] performed by our group amongst the same population, which showed a 3-day increase in gestational duration reduced the odds of neonatal death by 67% among preterm births (results not published). Results from this trial were also used to estimate the mean (for the control group) and SD (both groups) of gestational duration for preliminary power analysis.

In the prior trial, fundal heights were used to estimate gestational duration, with estimated gestational duration (days) = (final measured fundal height (cm) x 7) + time (days) between measurement and delivery date. In undernourished women, it is possible that this method underestimated gestational duration due to slower intrauterine growth and also might have affected the skew (e.g., if IUGR babies were more likely to be born preterm). However, the SD (16.5 days) approximates those commonly reported in other trials of DHA supplementation where GA was an outcome. Simulations were done to assess potential impact of different distributions, as detailed below.

Gestational duration has a left/negative skew. The severity of skew can vary by setting. There are limited data in Sierra Leone on the distribution of gestational duration, and none are high-quality due to lack of ultrasound dating and high frequency of late presentations making LMP unreliable. We opted to use our 2021 trial data for this purpose. First, the 2021 gestational duration data were compared between intervention and control groups of that trial to assess whether there might be issues using parametric tests such as a t-test for this analysis. Doing so revealed that residuals violated normality and that there was heteroscedasticity. This prompted further investigation as to whether parametric or non-parametric tests would be better suited to evaluate this outcome variable. As a next step, the distribution of gestational duration from the 2021 trial was abstracted by day of gestational duration (e.g., 0.08% of deliveries occurred at 24 + 5 days....0.24% of deliveries occurred at 31 + 3 days, 0.15% occurred at 31 + 4 days ... 1.98% occurred at 37 + 6 days, and so on). 10,000 simulated datasets with 700 rows (participants) were generated from this distribution (group 1), by sampling with replacement. 3 days were added to the distribution (i.e., distribution shifted rightward by 3 days), and 10,000 further simulated datasets with 700 rows were generated (group 2), again by sampling with replacement. These simulated datasets were merged, and a t-test was used to compare group 1 vs. group 2. Again, non-normality of residuals and heteroscedasticity were identified. Thus, the decision was made to plan to use non-parametric tests to compare groups.

It is known that second trimester and third trimester gestational age estimations by last menstrual period (LMP) are unreliable, while GA estimations done by ultrasound with standard biometrics in the second and third trimesters have measurement error. Judging by the 2021 trial results, we expect the median (IQR) of enrollment GA to be approximately 24 (18-28). Using ultrasound dating is likely to shift this to some extent (perhaps 2 weeks), as we expect growth restriction to impact fundal height more than US dating. In addition, attempts will be made to recruit at earlier GA in this trial, though it is not clear if this will be possible, in part because there are no high-quality data on when undernutrition develops during pregnancy. It may be that some pregnant women develop undernutrition primarily during second/third trimesters and thus would not be eligible for enrollment until that time. Thus, it was assumed that most women will be enrolled during the second and third trimesters. Using ultrasound, GA estimation in second trimester is considered to add +/- 14 days of error to the estimate (2SD = 14 days). This error can be added to the outcome measure using simulation-based power analysis.

Ten thousand simulations were undertaken wherein control and intervention groups were simulated using the aforementioned distribution (control) or the aforementioned distribution + 3 days (intervention). Random errors with mean = 0 and SD = 7 days were then added to both groups' distributions. The Wilcoxon-Mann-Whitney test was used to compare groups across all simulations and the percent with p-values < 0.05 was determined. Across iterations, it was determined that 510 individuals per group were needed to achieve 80% power; the distribution is shown below. All power analyses were done using R.

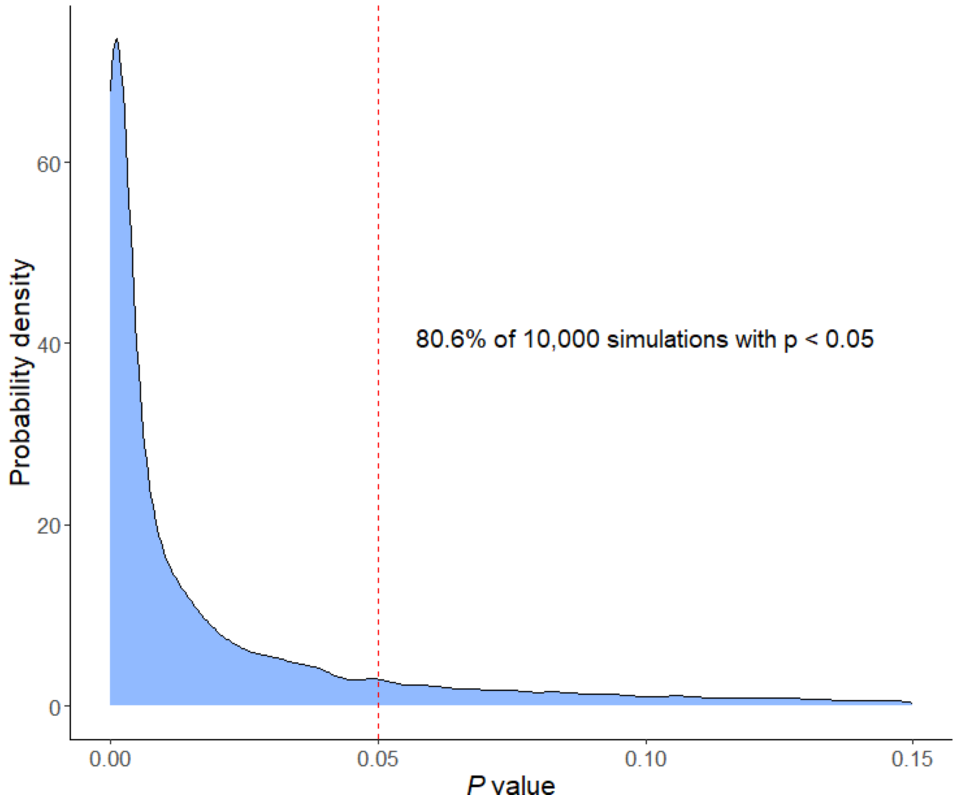

In the 2021 trial, 85% of participants remained in the trial (i.e., did not default) through delivery and had live singleton births. Assuming a similar 15% loss prior to primary outcome (gestational duration) determination, 510 participants per group for the primary outcome will require enrollment of 600

participants with enrollment GA ≤ 30 weeks per group, which will itself require 800 participants total per group. This estimation of 25% enrollment GA > 30 weeks is also drawn from the 2021 trial. In that study, 15% of participants had enrollment fundal height > 30 weeks. We expect fundal height to have underestimated GA by 2-3 weeks at fundal heights > 30 weeks due to intrauterine growth restriction. Evidence for this estimate comes from assessing birth weights across estimated gestational duration, which suggest a 2-3 week shift.

Protocol version 1.3 update: Given the multiple assumptions listed above regarding GA distribution, default rates, and singleton live birth rates, and the uncertainty therein, we performed a blinded review of enrollment GA and singleton live birth numbers in June of 2025, before trial enrollment was completed. In the course of this review, and in projecting numbers for the remaining ~100 enrollees based on historical proportions (e.g., proportions of enrollments with GA >30, as well as proportions of stillbirths, miscarriages, antenatal defaulters, and twin births), it was determined that the sample size for the primary outcome was likely to fall short of the required 1,020 by roughly 30-40 participants. The PI then decided to request ethics board approvals to expand trial enrollment by 60 participants, so as to assure adequate sample size to achieve 80% power for gestational duration outcome. Once approval was granted, this change was implemented.

Malawi Developmental Assessment Tool

For the second primary outcome – MDAT at 9 months of age – 800 participants per group will provide 80% power at a two-sided α of 0.05 to detect a 0.2 z-score increase in global MDAT score among offspring, assuming 28% of participants/offspring will either default or not be alive at 9 months of age, leaving 576 per group. The assumptions underlying this power analysis were drawn from two prior studies from our group, the Improved PUFA trial and the 2021 MamDutasi trial. The target 0.2 z-score increase is close to the 0.19 difference detected in the Improved PUFA trial, which tested adding DHA to therapeutic food for severely undernourished children. The 28% default/death estimation is based on the MamaDutasi trial, in which 25% of participants/offspring either were lost to follow-up or had died by 6 months after delivery. We assume 3% more participants will default between 6 -> 9 months based on the rate per month seen in that study.

The power analysis was done two ways. The expected distribution of MDAT scores in these 9mo children whose mothers were undernourished is unknown. First, a normal distribution was assumed, with control group mean = 0 and SD = 1.2, intervention group mean = 0.2 and SD = 1.2. This SD was drawn from the Improved PUFA trial. It is possible that the history of severe acute malnutrition among the children in this study increased the SD above what would be expected in COGENT; however, all of the children in COGENT will have been born to undernourished mothers, which also may increase the SD of their scores. Using a t-test, 576 participants per group provided 80.7% power across 10,000 simulations.

Second, given the uncertainty regarding the distribution and potential for left skew (common for developmental tests, more likely among a disadvantaged population), an alternative approach was undertaken. The distribution of MDAT z-scores from the Improved PUFA trial was abstracted. For the control group, 10,000 simulated datasets of populations sampled with replacement from this distribution were generated. 5,000 such simulations are plotted below, in which each line represents a single sample and drawn along a scale from yellow to green. As displayed, the distributions can vary by 0.5 – 1 z-score.

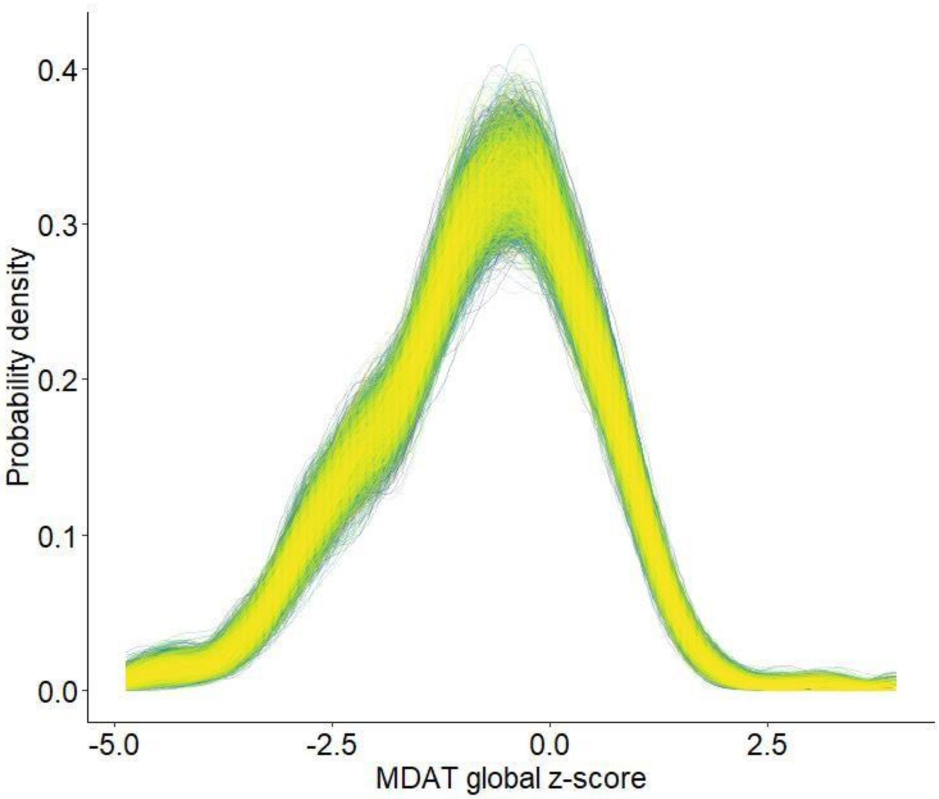

For the intervention group, 0.2 z-scores were added to the parent distribution and 10,000 simulated datasets were again generated, then merged with the control data. The Wilcox-Mann-Whitney test was used to compare all 10,000 simulated datasets and the power was 80%.

aPHQ-9 score

The study was not powered specifically for the CBT vs. no CBT comparison, which will only apply to the subgroup of participants who develop depression (aPHQ-9 ≥ 9) at the included clinics during the trial. A target effect size of 2-point improvement on the aPHQ-9 scale was chosen. This would correspond to a Cohen's d of 0.5, when accounting for an expected SD of 4 points, which was derived from an assessment of aPHQ-9 scores done in Pujehun District during the development of a perinatal counseling program. This Cohen's d is in the range of effect sizes seen in prior trials of CBT for peripartum depression.[34]

aPHQ-9 scores are anticipated to be best-analyzed using non-parametric tests, given their ordinal nature and skewness. Using G*Power and the Wilcoxon-Mann-Whitney test, 67 participants per group would be required to detect this effect size at a two-sided alpha of 0.05 with 80% power. In a simulation-based power analysis, the distribution of aPHQ-9 scores among those with scores ≥ 9 was abstracted from the aforementioned data collected during the counseling program development. Control group scores were simulated by sampling with replacement from this distribution. An effect size of 2 points' improvement was simulated for the intervention group, which was then sampled with replacement from this distribution. Groups were then compared using the Wilcoxon-rank-sum test, and a sample size of 59 per group was required to exceed 80% power at a two-sided alpha of 0.05. Given the lack of certainty about score distribution and variance in how scores may change with the intervention, it was conservatively decided to use the G*Power estimate, as it recommended a slightly larger sample size. Anticipating 10%

default/non-collection rate for outcome aPHQ-9 scores, 75 participants will be enrolled to achieve adequate power.

9.3 POPULATIONS FOR ANALYSES

Primary outcome analyses (gestational duration and MDAT in the M-RUSF factor, and aPHQ-9 in the CBT factor) will be done in separate modified intention-to-treat populations. For each of these three primary outcomes, the following modifications (for mITT) apply: (1) participants who are found to have an exclusion criterion after enrollment/randomization will be excluded when it is identified, (2) participants without the required outcome data / who did not have the outcome assessment done will not be included. Reasons for lack of outcome assessment include death or defaulting prior to its completion. There may also be cases where gestational duration be computed because a participant is temporarily lost to follow-up and, when ultimately found again, does not recall the date of birth and the study team is unable to identify this datapoint. All cases where participant data is not included in an outcome analysis will be detailed, with reason(s) why. Participants will contribute outcome data to all outcomes for which they meet mITT criteria e.g., if a participant meets mITT for gestational duration but not MDAT, they will be included in gestational duration but not MDAT.

There is an additional modification in the case of primary outcome gestational duration: this will only include singleton births to women with enrollment GA ≤ 30 weeks. This decision was made as a compromise between arguments in favor of more restrictive enrollment criteria:

- preceding data from studies showing DHA benefit generally only included women with enrollment GA < 20 weeks
- the mechanisms underlying pre-term birth might begin earlier in pregnancy
- there is a hypothesis that most benefit is derived from shifting early pre-term births right-ward

and those in favor of more broad criteria:

- many women in LMICs present later in pregnancy, and thus results of a trial restricting enrollment to, e.g., GA < 20 weeks would likely not apply to many of these women
- for some women, undernutrition does develop during the second and third trimesters of pregnancy, and it would be valuable to know if this intervention might help these women as well
- undernutrition is a state of total-body depletion, and thus it is possible that DHA supplementation even later in pregnancy might benefit these women and their children

Thus, ≤ 30 weeks was chosen as a compromise cut-off for gestational duration co-primary outcome.

The outcomes of twins will be reported, but will not be included in the main efficacy assessments (gestational duration, early preterm / preterm birth, SGA, offspring vital status, offspring birth measurements). We will likely not know if twins are present until time of delivery, and thus it is probable twins will be included in the study.

All participants will be included in safety analyses.

A subset of participants with baseline GA ≤ 24 weeks will be offered enrollment in a sub-study wherein ultrasounds will be collected serially every 4-6 weeks during pregnancy. This cut-off was chosen as a compromise between goals of including as many participants as possible and desiring to limit it to those in whom baseline GA is most likely to be accurate.

In a per-protocol analysis, only participants who missed ≤3 study visits will be included. In a separate per-protocol analysis, only participants who received at least 4 weeks of study food will be included.

Sub-group analyses will be done on the basis of baseline RBC-DHA content quartiles, baseline MUAC, enrollment gestational age, parity, and maternal age. For CBT, sub-groups will be ante- and postnatal depression.

9.4 STATISTICAL ANALYSES

9.4.1 GENERAL APPROACH

Categorical variables will be summarized as n (%). Continuous variables will first be plotted using probability density plots. If their distribution approximates normality, they will be summarized as mean (SD); otherwise, they will be summarized as median (IQR). Several key variables, such as gestational duration and MDAT, may be summarized using both. *P* values will be considered statistically significant when < 0.05. 95% CIs will be estimated for all between-group comparisons, including differences, ORs, and RRs. Neither *P* values nor 95% CIs will be adjusted for multiple comparisons.

9.4.2 ANALYSIS OF THE PRIMARY EFFICACY ENDPOINT(S)

Analyses will be performed on the modified intention-to-treat populations outlined **in section 9.3**. There will be no adjustment for multiplicity. The factorial components can be considered separate trials; it is expected that EPA and CBT could be additive in their effects but are not expected to be multiplicative. For co-primary outcomes gestational duration and MDAT in the M-RUSF+ vs. M-RUSF comparison, in this trial of a low-risk intervention in which outcomes may be correlated, it is deemed to be more important to maintain study power than to guard against inflating type I error.

Gestational duration
This will be calculated by adding the enrollment estimated gestational age (days) to the number of days between enrollment and date of delivery. The primary analysis will be done using the Wilcoxon-rank-sum test. The Hodges-Lehman (H-L) estimator will be used as the point estimate of the location shift. Because ties may be present, the 95% CI for the Hodges-Lehmann estimator will be computed using the normal approximation method with continuity correction (as in R's wilcox.test function).

Secondarily, adjusted analysis will be done using ordinal logistic regression. The proportional odds assumption will be assessed for treatment group; if deemed violated, an alternative will be chosen, such as partial proportional odds ordinal logistic regression. Covariates will be maternal age, GA at enrollment, and number of prior pregnancies. OR (95% CI) and difference in model derived medians with bootstrapped 95% CI will be estimated.

Malawi Developmental Assessment Tool
This will be calculated by the tablet program as an age-adjusted global z-score. Residual normality and homoscedasticity will be evaluated prior to use of linear regression. If assumptions are met, linear regression will be used to compare groups, with difference (95% CI) reported. If assumptions are not

met, Wilcoxon-rank-sum test will be done, with H-L median of differences and 95% CI reported. This procedure is necessary because we do not know / cannot predict how MDAT modeling errors will distribute in 9 mo children born to Sierra Leonean women who were undernourished during pregnancy. This unadjusted analysis will be the primary outcome analysis.

Similarly, it is unclear which variables might be valuable for adjustment. As a result, adjusted analyses will be considered exploratory, with possibilities including gestational age at enrollment, gestational weight/length, sex, maternal education level, maternal HIV status, and number of children sleeping in home.

Adapted Patient Health Questionnaire-9

In the same manner as the original PHQ-9, scores will be calculated by adding up the points across the 9-element test (min score 0, max score 27). This score is ordinal in nature. The primary outcome aPHQ-9 will be collected 8 weeks after enrollment in CBT vs. no CBT comparison, if possible; if the participant does not present for the 8-week timepoint collection, an aPHQ-9 score from alternative timepoint will be chosen in the following preference order: 9, 10, 11, 12, 7, 6, 13, 14, 15, or 16 weeks. The Wilcoxon-rank-sum test will be used to compare groups. The H-L estimator will be used as the point estimate of the location shift. Because ties will be present, the 95% CI for the Hodges-Lehmann estimator will be computed using the normal approximation method with continuity correction (as in R's wilcox.test function).

As a secondary analysis, adjusted analysis will be done using ordinal logistic regression. The proportional odds assumption will be assessed for treatment group; if deemed violated, an alternative will be chosen, such as partial proportional odds ordinal logistic regression. Covariates will be baseline aPHQ-9 score, maternal age, and maternal education level. OR (95% CI) and difference in model derived medians with bootstrapped 95% CI will be estimated.

9.4.3 ANALYSIS OF THE SECONDARY ENDPOINT(S)

These pre-planned secondary analyses will not depend on results of primary outcome analyses. Analyses will be performed on the modified intention-to-treat population among singleton live births. There will be no adjustment for multiplicity.

<u>For the M-RUSF+ vs. M-RUSF comparison:</u>

Early preterm birth

ePTB will be defined as duration of gestation (enrollment GA estimate + time between enrollment and date of delivery) < 34 weeks. It is a binary outcome. Logistic regression will be used to compare groups, with OR (95% CI) estimated.

Birth weight and length

These will be measured directly in duplicate with measurements averaged, often within 1 day of delivery and almost always within 2 days of delivery. Following expected confirmation of residual normality and homoscedasticity, values will be compared between treatment groups using linear regression with differences (95% CIs) estimated.

Low birth weight

PBW will be defined as birth weight < 2.5 kg. It is a binary outcome. Logistic regression will be used to compare groups, with OR (95% CI) estimated.

MDAT sub-domain z-scores
The same procedure will be used as described above for the MDAT global z-score primary efficacy outcome.

Neonatal mortality
Neonatal mortality will be defined as live birth with subsequent death < 28 days from date of birth. It is a binary outcome. Time-to-event analysis will be used to compare groups, with censoring for default during follow-up. The proportional hazards assumption will be assessed prior to use of Cox proportional hazards regression. HR (95% CI) will be estimated.

Preterm birth
PTB will be defined as duration of gestation (enrollment GA estimate + time between enrollment and date of delivery) < 37 weeks. It is a binary outcome. Logistic regression will be used to compare groups, with OR (95% CI) estimated.

aPHQ-9 scores
PHQ-9 scores will be measured every 4 weeks antenatally and each score will be tabulated by summing points on the 9-item scale (min 0, max 27). This is a longitudinal ordinal outcome. Longitudinal ordinal logistic regression will be used to compare groups, with random effect for participant intercept and slope. Treatment x time interaction will be the parameter of interest. OR with 95% CI will be estimated.

RBC-DHA content
Expressed as % of fatty acids. This variable is expected to have non-normal residuals and heteroscedasticity. Analysis will be done using the Wilcoxon-Mann-Whitney test. Median of differences and 95% CI will be estimated.

Placental weight
Placental weight will be measured as close to delivery as possible. Following expected confirmation of residual normality and homoscedasticity, values will be compared between treatment groups using linear regression with differences (95% CIs) estimated.

SGA
SGA will be defined as birth weight-for-gestational-age < 10th percentile. Birth weight will be measured as closely to time of delivery as possible. GA at birth will be calculated as enrollment GA estimate + time between enrollment and date of delivery. It is a binary outcome. Logistic regression will be used to compare groups, with OR (95% CI) estimated.

aPHQ-9 < 9
Remaining below 9 throughout antepartum period. Logistic regression will be used to compare groups. ORs with 95% CIs will be estimated.

aPHQ-9 < 5.
Remaining below 5 throughout antepartum period. Will be compared using logistic regression. ORs with 95% CIs will be estimated.

<u>For CBT vs. no CBT comparisons</u>**:**

Change in aPHQ-9 score between baseline and endline

This will be compared using the Wilcoxon rank-sum test. The H-L estimator will be used as the point estimate of the location shift. Because ties will be present, the 95% CI for the Hodges-Lehmann estimator will be computed using the normal approximation method with continuity correction (as in R's wilcox.test function).

Reduction in aPHQ-9 score from baseline to endline, at thresholds

Binary. Reduction from baseline of ≤3, >3, >5, >9, and >50% will be compared using logistic regression. ORs with 95% CIs will be estimated.

Endline aPHQ-9 score < 5.

Binary. Will be compared using logistic regression. ORs with 95% CIs will be estimated.

Endline aPHQ-9 < 9

Logistic regression will be used to compare groups. ORs with 95% CIs will be estimated.

Gestational duration

As specified previously. Only among antepartum participants.

Birth weight and length

Among participants enrolled with antepartum depression. These will be measured directly in duplicate with measurements averaged, often within 1 day of delivery and almost always within 2 days of delivery. Following expected confirmation of residual normality and homoscedasticity, values will be compared between treatment groups using linear regression with differences (95% CIs) estimated.

Gestational weight gain

Among participants enrolled with antepartum depression. This will be measured fortnightly and compared from time of enrollment into CBT until last visit prior to delivery. Values will be compared between treatment groups using linear regression with differences (95% CIs) estimated.

aPHQ-9 scores following endline score

Extending beyond the primary outcome timepoint. As part of COGENT, PHQ-9 scores will be measured every 4 weeks antenatally and at each postnatal visit (6 weeks, 3 months, 6 months, 9 months). The Wilcoxon rank-sum test will be used to compare scores at each postnatal visit among participants enrolled antepartum. The H-L estimator will be used as the point estimate of the location shift. Because ties will be present, the 95% CI for the Hodges-Lehmann estimator will be computed using the normal approximation method with continuity correction (as in R's wilcox.test function).

Longitudinal ordinal logistic regression will also be used to compare groups, with random effect for participant intercept and slope. All aPHQ-9s measured after enrollment will be included. Treatment x time interaction will be the parameter of interest. Baseline aPHQ-9 will be a covariate. OR with 95% CI will be estimated.

MDAT z-scores

As specified previously.

Adjusted analyses

Adjusted analyses will also be done for secondary outcomes change in aPHQ-9 between baseline and endline, reduction of aPHQ-9 between baseline and endline above thresholds (above), endline aPHQ-9 < 5. Analysis will be done for continuous outcomes using ordinal logistic regression. Analysis for binary outcomes will be done using logistic regression. Covariates will be baseline aPHQ-9 score, maternal age, and maternal education level. OR (95% CI) and difference in model derived medians with bootstrapped 95% CI will be estimated.

9.4.4 SAFETY ANALYSES

The primary safety endpoints will be maternal death, infant death, miscarriage, stillbirth, postnatal delivery (>42 weeks), maternal prepartum hospitalization, and peripartum complication (e.g., preeclampsia suspected) requiring transfer to facility with higher level of care. These will likely be presented in a table using summary statistics and compared between groups using logistic regression. Diarrhea rates will also be reported.

9.4.5 BASELINE DESCRIPTIVE STATISTICS

Baseline descriptive statistics will be presented as per the procedure outlined in **section 9.4.1.** There will be no statistical comparison between groups.

9.4.6 PLANNED INTERIM ANALYSES

Not applicable

9.4.7 SUB-GROUP ANALYSES

Sub-group analyses for primary and secondary endpoints in the M-RUSF+ vs. M-RUSF comparison will be done on the basis of baseline RBC-DHA content, baseline MUAC, enrollment gestational age, parity, and maternal age. We will likely model “sub-groups” as interaction terms between study group and variable of interest with the variable of interest taken as a continuous variable, with spline terms included if the relationship appears non-linear. Baseline RBC-DHA content was chosen because of prior RCT results suggesting greater benefit of antenatal DHA supplementation among mothers with lower baseline DHA status. Baseline MUAC was chosen as a surrogate for degree of undernutrition; it is possible the intervention might have differential impacts on this basis. Enrollment gestational age was chosen because most prior trials showing benefit of DHA enrolled more stringently, e.g. GA < 20 weeks, and it is possible those who receive supplementation longer might derive more benefit. Maternal age was chosen on the basis of a secondary analysis of the prior Sierra Leone maternal undernutrition study by our group, wherein offspring of adolescent mothers were found to derive less benefit from the combined intervention of food + anti-infective measures than adult mothers.[35] Parity was chosen because it predicts gestational duration.

Subgroups analysis for aPHQ-9 score for the CBT vs. no CBT comparison will be done for ante- vs. postnatal depression, maternal education level, and baseline aPHQ-9 score. P values will be estimated for the interaction terms in ordinal logistic regression. Baseline aPHQ-9 score will be modeled as a continuous variable. In addition, we will assess whether M-RUSF+ interacts with CBT to affect aPHQ-9 scores. This will be done by creating an interaction term between CBT and M-RUSF+ allocation in the relevant regressions.

9.4.8 TABULATION OF INDIVIDUAL PARTICIPANT DATA

Individual participant data will not be provided in the study report.

9.4.9 EXPLORATORY ANALYSES

We will explore whether M-RUSF+ might reduce the incidence of depression (PHQ-9 ≥ 9), increase placental weight, alter placental tissues, increase birth chest and head circumference. We will explore whether CBT improves breastfeeding practices.

Intrauterine growth parameters will be assessed on serial USs among a subset of participants with enrollment GA ≤ 24 weeks. Machine learning algorithms will be developed to assess intrauterine growth longitudinally as well as assess changes in head circumference, estimated weight, and femur length. Data are expected to be analyzed using linear mixed models with random effects for participant and time.

If M-RUSF+ prolongs gestation and improves MDAT global z-scores, we will perform a mediation analysis with gestational duration as the mediating variable.

We reserve the right to explore additional analyses that become of interest, and will label them as exploratory in nature in publications.

10 SUPPORTING DOCUMENTATION AND OPERATIONAL CONSIDERATIONS

10.1 REGULATORY, ETHICAL, AND STUDY OVERSIGHT CONSIDERATIONS

10.1.1 INFORMED CONSENT PROCESS

10.1.1.1 CONSENT/ASSENT AND OTHER INFORMATIONAL DOCUMENTS PROVIDED TO PARTICIPANTS

Consent forms describing in detail the study intervention, study procedures, and risks are given to the participant and written (or thumb print) documentation of informed consent is required prior to administering study intervention. The following consent materials are submitted with this protocol:

M-RUSF+ vs. M-RUSF study consent
M-RUSF+ vs. M-RUSF study assent
CBT vs. no CBT study consent
CBT vs. no CBT study assent

10.1.1.2 CONSENT PROCEDURES AND DOCUMENTATION

Head chiefs for all catchment areas will be informed of the study and their consent will be obtained prior to trial initiation. This process has been part of the clinical trials routine for the PI's research group in Malawi and Sierra Leone for two decades and has been successful.

All consent processes will be performed by Sierra Leonean research nurses in participant native language (e.g., Mende, Krio, etc.). Based on prior experience, it is expected that most participants will not be literate. Thus, much of the consent process is expected to be verbal in nature, with thumbprint consent documentation in the event of participant consent or assent. Because so much of the consent process will rely on our research nurses verbalizing study procedures, potential benefits, risks etc., these nurses will undergo extensive education and training around the trial and consent processes prior to study initiation. This process is essential for the trial to reach as many of the most vulnerable Sierra Leonean women as possible. In addition, for women who are illiterate or unable to understand the consent proceedings (e.g., due to low level of schooling), an impartial witness will be present during the entire consent procedure to witness it and sign the informed consent form.

Informed consent is a process that is initiated prior to the individual's agreeing to participate in the study and continues throughout the individual's study participation. Consent forms will be SLESRC, PBSL, and WUSTL IRB-approved and the participant will be asked to read and review the document if they are literate; otherwise, Sierra Leonean research nurses will read and review the document for participants in their native language (with an impartial witness also present). The Sierra Leonean research nurses will explain the research study to the participant and answer any questions that may arise. A verbal explanation will be provided in terms suited to the participant's comprehension of the purposes, procedures, and potential risks of the study and of their rights as research participants. Participants will have the opportunity to carefully review the written consent form and/or ask questions prior to signing. The participants will have the opportunity to discuss the study with their family or surrogates or think

about it prior to agreeing to participate. The participant will sign or, if illiterate, apply a thumbprint to the informed consent document prior to joining the study. Participants will be informed that participation is voluntary and that they may withdraw from the study at any time, without prejudice. The informed consent process will be conducted and documented in the source document (including the date), and the form signed by all necessary parties, before the participant undergoes any study-specific procedures. The rights and welfare of the participants will be protected by emphasizing to them that the quality of their medical care will not be adversely affected if they decline to participate in this study.

In the event of enrolling a participant under 18 years of age and unmarried, parental/guardian consent will be required, with participant assent. The same procedures as described above will be undertaken.

10.1.2 STUDY DISCONTINUATION AND CLOSURE

This study may be temporarily suspended or prematurely terminated if there is sufficient reasonable cause. If the study is prematurely terminated or suspended, the PI will promptly inform study participants, the IRBs, and sponsor and will provide the reason(s) for the termination or suspension. Study participants will be contacted, as applicable, and be informed of changes to the schedule of study visits.

Circumstances that may warrant termination or suspension include, but are not limited to:

- Determination of unexpected, significant, or unacceptable risk to participants
- Insufficient compliance to protocol requirements
- Data that are not sufficiently complete and/or evaluable

Study may resume once concerns about safety, protocol compliance, and data quality are addressed, and satisfy the PI and IRB.

10.1.3 CONFIDENTIALITY AND PRIVACY

Participant confidentiality and privacy are strictly held in trust by the participating investigators and their staff. This confidentiality is extended to cover testing of biological samples and genetic tests in addition to the clinical information relating to participants. Therefore, study data and all other information generated will be held in strict confidence. No information concerning the study or the data will be released to any unauthorized third party without PI approval.

All research activities will be conducted in as private a setting as possible. Because the study will largely take place at government-run antenatal clinics, there will be limitations as to the degree of privacy possible. However, for sensitive activities such as gestational age ultrasounds and cognitive behavioral therapy, privacy will be maintained by using a separate room or facility.

Study participant contact information will be securely stored at the two research bases of operation in Pujehun and Kono Districts in Sierra Leone for internal use during the study. At the end of the study, all records will continue to be kept in a secure location for as long a period as dictated by the reviewing IRB.

Study participant research data, which is for purposes of statistical analysis and scientific reporting, will be transmitted to and stored on a WUSTL-based secure cloud computing site, Box. An exception is ultrasound videos, which will be stored using the secure Butterfly App and, once internet service is reached, will be uploaded to WUSTL Box. Ultrasound video recordings will also be made available to collaborators at UNC as per the data sharing agreement. Such stored data will not include the participant's contact or identifying information. Rather, individual participants and their research data will be identified by a unique study identification number. The study data entry and study management systems used by research staff will be secured and password protected. At the end of the study, all study databases will be de-identified and archived securely at WUSTL.

10.1.4 FUTURE USE OF STORED SPECIMENS AND DATA

Data collected for this study will be analyzed and stored at WUSTL in the PI's secure cloud database. After the study is completed, the de-identified, archived data will be transmitted to and stored at WUSTL, for use by other researchers including those outside of the study once approval has been obtained by the PI.

With the participant's approval and as approved by local IRBs, de-identified biological samples will be stored at WUSTL in the PI's laboratory freezers. These samples could be used to study various physiologic and pathologic processes, including but not limited to preterm labor, neonatal mortality, lipid metabolism and its genetic implications, DHA trafficking, metabolomics, infant growth, and cognitive development. The potential for future use will be explicit in the trial consent forms. The WUSTL repository will also have a code-link that will allow linking the biological specimens with the phenotypic data from each participant, maintaining the blinding of the identity of the participant. This potential use will be made clear in the consent.

During the conduct of the study, an individual participant can choose to withdraw consent to have biological specimens stored for future research. However, withdrawal of consent with regard to bio-sample storage may not be possible after the study is completed, as there is unlikely to be contact with study participants after their involvement has finished.

10.1.5 KEY ROLES, STUDY TEAM, AND STUDY GOVERNANCE

Principal Investigators: Mark Manary, MD, Washington University in St. Louis
National PI, Aminata Koroma MS. Director of Nutrition, Ministry of Health & Sanitation of Sierra Leone
Co-PI: Kevin Stephenson, MD, Washington University in St. Louis
Co-Investigator: D. Taylor Hendrixson, MD, University of Washington
Study Monitor: Donna Wegner, Washington University in St. Louis
Local Monitor: Hannah Yankson
Medical Monitor: Indi Trehan, MD MPH, University of Washington
Pujehun District Collaborator, Dr. Amara Stevens Ngegbai MD
Ibrahim Affie Bangura, Director of Finances and Human Resources
Joshua Abioseh Duncan, Coordinator for Cognitive Behavioral Therapy, Mental Health Coalition of Sierra Leone
Chief of Food Production, Tabita Kamara PhD, Project Peanut Butter

Research Associate TBA, Washington University in St. Louis
Nurse-midwives (4) fluent in local language Mende, having completed nursing school and midwife program and with 5 years' experience, Project Peanut Butter
Counselors, Project Peanut Butter
Cognitive development assessment team, Project Peanut Butter
Drivers, Project Peanut Butter

10.1.6 SAFETY OVERSIGHT

The data and safety monitoring plan will involve a chain of reporting of AEs and SAEs within predetermined time windows as indicated in section 8.3. All research staff will be empowered to report safety concerns to the Research Associate (RA).

10.1.7 CLINICAL MONITORING

Clinical site monitoring is conducted to ensure that the rights and well-being of trial participants are protected, that the reported trial data are accurate, complete, and verifiable, and that the conduct of the trial is in compliance with the currently approved protocol/amendment(s), with International Conference on Harmonisation Good Clinical Practice (ICH GCP), and with applicable regulatory requirement(s).

The foundational component of study monitoring will be the responsibility of all research staff who interact with participants, provide clinical care, and collect data. The nurse-midwives, RA, and visiting MDs will ensure professional and appropriate clinical care for participants each study day. There will be frequent training for consenting, ultrasonography, anthropometric measurements, and food disbursement to ensure integrity of study procedures. Daily checks of all data cards prior to participants leaving the clinic will be done to evaluate for missing or illogical data. Participants will be asked to bring back their empty sachets each visit to assess for correct food disbursement. Medication provision will be documented with dates on study cards. Supply lists will be kept updated daily to ensure there are no stock-outs.

In addition, the PI Mark Manary, Kevin Stephenson, and Taylor Hendrixson will provide on-site monitoring at least every 2 months throughout the study. This will involve visit clinics each day with the research team, overseeing each aspect of care and data collection, and evaluation of data entry.

The database will be checked each week remotely from St. Louis for missing data and illogical entries. Double entry from primary data cards will be done and checked against the initial entry for errors.

10.1.8 QUALITY ASSURANCE AND QUALITY CONTROL

As stated in section 10.1.7, the most important aspect of quality assurance and control will be the daily work of the research staff in the field in Sierra Leone. Protocols have been developed for each technical procedure, including ultrasonography, blood spot collection, cord blood collection, and placental weight measurement. All relevant individuals will undergo training prior to trial initiation and intermittently

during the trial at least q6mo or more frequently if issues arise. All data collected will be checked daily prior to discharging a participant from clinic to assess for missingness and implausibility. There will be checks on accuracy of study food disbursement and timeliness of medication administration by comparison of expectations based on scheduling with documented provision. There will be assessments as to the quality of CBT with random visits by the clinical coordinator overseeing the counselors. Data cards will be scanned and transmitted to the secure cloud database weekly alongside the updated database for review for completeness and plausibility. Any missing data or data anomalies will be communicated to the sites for clarification/resolution.

10.1.9 DATA HANDLING AND RECORD KEEPING

10.1.9.1 DATA COLLECTION AND MANAGEMENT RESPONSIBILITIES

Data collection is the responsibility of the clinical trial staff at the site under the supervision of the Research Associate. The RA is responsible for ensuring the accuracy, completeness, legibility, and timeliness of the data reported. The RA will have oversight in the form of remote monitoring using the cloud repository with weekly assessments of all data collected as well as in-person oversight with at least bi-monthly visits from the PI or designees.

Standardized hardcopy study cards will be used for recording data for each participant enrolled in the study. After clinic, these data will be entered daily into a secured electronic database at the research centers in Sierra Leone, followed by upload to the WUSTL-based secure cloud repository. Hardcopy study cards will also be scanned and uploaded to the same repository weekly. All data will be double-entered by different individuals. Discrepancies will be assessed by review of the primary data card.

Ultrasound videos will be stored on the Butterfly App and the secure cloud-based repository once WiFi is accessible. UNC collaborator has also been granted access to deidentified videos to be shared through Butterfly and stored securely.

10.1.9.2 STUDY RECORDS RETENTION

Study documents should be retained for a minimum of 6 years after the study has completed, in line with the WUSTL IRB policy.

10.1.10 PROTOCOL DEVIATIONS

A protocol deviation is any noncompliance with the clinical trial protocol. The noncompliance may be either on the part of the participant, the investigator, or the study site staff. As a result of deviations, corrective actions are to be developed by the site and implemented promptly.

It is the responsibility of research staff to use continuous vigilance to identify deviations. It is the responsibility of the lead research assistants in Sierra Leone to report deviations to the PI within 1 working day of identification of the protocol deviation. All deviations must be addressed in study source documents and reported to the Principal Investigator. Protocol deviations must be sent to PBSL and the

reviewing Institutional Review Board (IRB) per their policies. All research team members are responsible for knowing and adhering to PBSL and the reviewing IRB requirements.

10.1.11 PUBLICATION AND DATA SHARING POLICY

The PI will determine authorship on primary and secondary publications of data derived from this trial and published by the PI's research group. De-identified data will be shared with PI approval beginning 6 months after primary trial publication.

10.1.12 CONFLICT OF INTEREST POLICY

The funding agency will play no role in the design, implementation, analysis, or decision to publish results of this trial. Persons with actual conflicts of interest will not be allowed to participate in the trial in any manner. Furthermore, persons who have a perceived conflict of interest will be required to have such conflicts managed in a way that is appropriate to their participation in the design and conduct of this trial.

10.2 ADDITIONAL CONSIDERATIONS

Not applicable

10.3 ABBREVIATIONS

AE	Adverse event
aPHQ-9	Adapted PHQ-9
BMI	Body-mass index
CBT	Cognitive behavioral therapy
CFR	Code of Federal Regulations
CHW	Community health worker
FAMLI	Fetal Age Machine Learning Initiative
GA	Gestational age
ICH GCP	International Conference on Harmonization Good Clinical Practice
IRB	Institutional Review Board
LAZ	Length-for-age z-score
MDAT	Malawi Developmental Assessment Tool
M-RUSF	Maternal ready-to-use supplementary food
M-RUSF+	Maternal ready-to-use supplementary food+
mITT	Modified intention-to-treat
MUAC	Mid-upper arm circumference
PHQ-9	Patient Health Questionnaire-9
PPB	Project Peanut Butter
RA	Research Associate
RUSF	Ready-to-use supplementary food
RUTF	Ready-to-use therapeutic food
SAE	Serious adverse event
SLESRC	Sierra Leone Ethics Review Committee
UP	Unanticipated problem
US	United States of America
WLZ	Weight-for-length z-score
WUSTL	Washington University in St. Louis

10.4 PROTOCOL AMENDMENT HISTORY

Version	Date	Description of Change	Brief Rationale
1.1	1-Aug-2023	Inclusion criteria (age cut-off/marital status for consent vs. assent), add impartial witness for illiterate enrollees, edit food stability testing plan	To align with Pharmacy Board of Sierra Leone guidelines
1.2	6-March-2024	Addition of serial ultrasound assessments on subgroup of participants	To improve understanding of intrauterine growth in undernutrition
1.3	8-July-2025	Increased trial enrollment to 1,660 from 1,600	To assure adequate sample size for gestational duration outcome

11 REFERENCES

1. Carshon-Marsh R, Aimone A, Ansumana R, et al. Child, maternal, and adult mortality in Sierra Leone: nationally representative mortality survey 2018–20. *The Lancet Global Health*. 2022-01-01 2022;10(1):e114-e123. doi:10.1016/s2214-109x(21)00459-9

2. Desyibelew HD, Dadi AF. Burden and determinants of malnutrition among pregnant women in Africa: A systematic review and meta-analysis. *PLoS One*. 2019-09-06 2019;14(9):e0221712. doi:10.1371/journal.pone.0221712

3. Young F, Melissa, Ramakrishnan U. Maternal Undernutrition before and during Pregnancy and Offspring Health and Development. *Ann Nutr Metab*. 2020-01-01 2020;76(Suppl. 3):41-53. doi:10.1159/000510595

4. Stephenson K, Callaghan-Gillespie M, Maleta K, et al. Low linoleic acid foods with added DHA given to Malawian children with severe acute malnutrition improve cognition: a randomized, triple blinded, controlled clinical trial. *Am J Clin Nutr*. 2021-11-02 2021;doi:10.1093/ajcn/nqab363

5. Callaghan-Gillespie M, Schaffner AA, Garcia P, et al. Trial of ready-to-use supplemental food and corn-soy blend in pregnant Malawian women with moderate malnutrition: a randomized controlled clinical trial. *Am J Clin Nutr*. 2017-04-01 2017;106(4):1062-1069. doi:10.3945/ajcn.117.157198

6. Hendrixson DT, Smith K, Lasowski P, et al. A novel intervention combining supplementary food and infection control measures to improve birth outcomes in undernourished pregnant women in Sierra Leone: A randomized, controlled clinical effectiveness trial. *PLOS Medicine*. 2021-09-28 2021;18(9):e1003618. doi:10.1371/journal.pmed.1003618

7. Middleton P, Gomersall JC, Gould JF, Shepherd E, Olsen SF, Makrides M. Omega-3 fatty acid addition during pregnancy. *Cochrane Database Syst Rev*. 2018-11-15 2018;2018(11)doi:10.1002/14651858.cd003402.pub3

8. Best KP, Gibson RA, Makrides M. ISSFAL statement number 7 – Omega-3 fatty acids during pregnancy to reduce preterm birth. *Prostaglandins, Leukotrienes and Essential Fatty Acids*. 2022-11-01 2022;186:102495. doi:10.1016/j.plefa.2022.102495

9. Makrides M, Best K, Yelland L, et al. A Randomized Trial of Antenatal n−3 Fatty Acid Supplementation and Preterm Delivery. *New England Journal of Medicine*. 2019-09-12 2019;381(11):1035-1045. doi:10.1056/nejmoa1816832

10. Simmonds L, Sullivan T, Skubisz M, et al. Omega‐3 fatty acid supplementation in pregnancy—baseline omega‐3 status and early preterm birth: exploratory analysis of a randomised controlled trial. *BJOG*. 2020-07-01 2020;127(8):975-981. doi:10.1111/1471-0528.16168

11. Brenna JT. Animal studies of the functional consequences of suboptimal polyunsaturated fatty acid status during pregnancy, lactation and early postnatal life. *Maternal & Child Nutrition*. 2011-04-01 2011;7:59-79. doi:10.1111/j.1740-8709.2011.00301.x

12. Bourre JM. Effects of nutrients (in food) on the structure and function of the nervous system: update on dietary requirements for brain. Part 2 : macronutrients. *J Nutr Health Aging*. Sep-Oct 2006;10(5):386-99.

13. McNamara RK, Carlson SE. Role of omega-3 fatty acids in brain development and function: potential implications for the pathogenesis and prevention of psychopathology. *Prostaglandins Leukot Essent Fatty Acids*. Oct-Nov 2006;75(4-5):329-49. doi:10.1016/j.plefa.2006.07.010

14. Martinez M. Tissue levels of polyunsaturated fatty acids during early human development. *J Pediatr*. Apr 1992;120(4 Pt 2):S129-38. doi:10.1016/s0022-3476(05)81247-8

15. Gould JF, Makrides M, Gibson RA, et al. Neonatal Docosahexaenoic Acid in Preterm Infants and Intelligence at 5 Years. *New England Journal of Medicine*. 2022-10-27 2022;387(17):1579-1588. doi:10.1056/nejmoa2206868

16. Klatt KC, Mcdougall MQ, Malysheva OV, et al. Antenatal choline supplementation improves biomarkers of maternal docosahexaenoic acid status among pregnant participants consuming supplemental DHA: a randomized controlled trial. *Am J Clin Nutr*. 2022-05-16 2022;doi:10.1093/ajcn/nqac147

17. Shortis E, Warrington D, Whittaker P. The efficacy of cognitive behavioral therapy for the treatment of antenatal depression: A systematic review. *J Affect Disord*. Jul 1 2020;272:485-495. doi:10.1016/j.jad.2020.03.067

18. O'Connor E, Rossom RC, Henninger M, Groom HC, Burda BU. Primary Care Screening for and Treatment of Depression in Pregnant and Postnatal Women. *JAMA*. 2016-01-26 2016;315(4):388. doi:10.1001/jama.2015.18948

19. Huang L, Zhao Y, Qiang C, Fan B. Is cognitive behavioral therapy a better choice for women with postnatal depression? A systematic review and meta-analysis. *PLoS One*. 2018-10-15 2018;13(10):e0205243. doi:10.1371/journal.pone.0205243

20. Olsen SF, Sorensen JD, Secher NJ, et al. Randomised controlled trial of effect of fish-oil supplementation on pregnancy duration. *Lancet*. Apr 25 1992;339(8800):1003-7. doi:10.1016/0140-6736(92)90533-9

21. Wood JL, Allison RG. Effects of consumption of choline and lecithin on neurological and cardiovascular systems. *Fed Proc*. Dec 1982;41(14):3015-21.

22. Schermuly-Haupt M-L, Linden M, Rush AJ. Unwanted Events and Side Effects in Cognitive Behavior Therapy. *Cognit Ther Res*. 2018-06-01 2018;42(3):219-229. doi:10.1007/s10608-018-9904-y

23. Simopoulos AP. Human requirement for N-3 polyunsaturated fatty acids. *Poult Sci*. Jul 2000;79(7):961-70. doi:10.1093/ps/79.7.961

24. Koletzko B, Cetin I, Thomas Brenna J. Dietary fat intakes for pregnant and lactating women. *British Journal of Nutrition*. 2007-11-01 2007;98(5):873-877. doi:10.1017/s0007114507764747

25. Carlson SE, Gajewski BJ, Valentine CJ, et al. Higher dose docosahexaenoic acid supplementation during pregnancy and early preterm birth: A randomised, double-blind, adaptive-design superiority trial. *EClinicalMedicine*. 2021-06-01 2021;36:100905. doi:10.1016/j.eclinm.2021.100905

26. Makrides M, Gibson RA, Mcphee AJ, et al. Effect of DHA Supplementation During Pregnancy on Maternal Depression and Neurodevelopment of Young Children. *JAMA*. 2010-10-20 2010;304(15):1675. doi:10.1001/jama.2010.1507

27. Carlson SE, Colombo J, Gajewski BJ, et al. DHA supplementation and pregnancy outcomes. *Am J Clin Nutr*. Apr 2013;97(4):808-15. doi:10.3945/ajcn.112.050021

28. Yan J, Jiang X, West AA, et al. Maternal choline intake modulates maternal and fetal biomarkers of choline metabolism in humans. *Am J Clin Nutr*. May 2012;95(5):1060-71. doi:10.3945/ajcn.111.022772

29. Otten J, Hellwig J, Meyers L, eds. *Dietary reference intakes: the essential guide to nutrient requirements*. National Academy of Sciences; 2006.

30. *Dietary Reference Intakes for Vitamin A, Vitamin K, Arsenic, Boron, Chromium, Copper, Iodine, Iron, Manganese, Molybdenum, Nickel, Silicon, Vanadium, and Zinc*. 2001.

31. *Dietary Reference Intakes for Vitamin C, Vitamin E, Selenium, and Carotenoids*. 2000.

32. James G, Stephenson K, Callaghan-Gillespie M, et al. Docosahexaenoic Acid Stability in Ready-to-Use Therapeutic Food. *Foods*. 2023-01-09 2023;12(2):308. doi:10.3390/foods12020308

33. Pokaprakarn T, Prieto JC, Price JT, et al. AI Estimation of Gestational Age from Blind Ultrasound Sweeps in Low-Resource Settings. *NEJM Evidence*. 2022-04-26 2022;1(5)doi:10.1056/evidoa2100058

34. Sockol LE. A systematic review of the efficacy of cognitive behavioral therapy for treating and preventing perinatal depression. *J Affect Disord*. May 15 2015;177:7-21. doi:10.1016/j.jad.2015.01.052

35. Koroma AS, Ellie M, Bangura K, et al. Supplementary feeding and infection control in pregnant adolescents—A secondary analysis of a randomized trial among malnourished women in Sierra Leone. *Maternal & Child Nutrition*. 2023-01-01 2023;19(1)doi:10.1111/mcn.13456
