# Supplementary material for: Cognitive Behavioral Therapy for Individuals With Low Literacy and Perinatal Depression: A Randomized Clinical Trial
Source: JAMA Netw Open. 2026 May 7;9(5):e2611101. doi: 10.1001/jamanetworkopen.2026.11101 (PMC13153995; doi:10.1001/jamanetworkopen.2026.11101)
Supplement: Supplement 2. — eMethods. Sample Size, Randomization, Adapted CBT Intervention Procedure, Data Analysis, and Data Management eTable 1. Selected Baseline Characteristics of Enrolled Participants by Study Arm and Presence of Primary Outcome Data eTable 2. Adjusted Efficacy Outcomes eTable 3. Efficacy Outcomes Stratified by Baseline aPHQ-9 Score eTable 4. Efficacy Outcomes From a Sensitivity Analysis Imputing a 2-Point Worsening in aPHQ-9 Scores in Place of Missing Outcome Data eTable 5. Efficacy Outcomes From a Sensitivity Analysis Imputing a 9-Point Improvement in aPHQ-9 Scores in Place of Missing Outcome Data eTable 6. Distribution of Postintervention Assessment Timing eTable 7. Efficacy Outcomes Stratified by Timing of Outcome Assessment eTable 8. Efficacy Outcomes Stratified by Session Attendance eFigure 1. Adapted PHQ-9 (aPHQ-9) Questionnaire eFigure 2. Effect of CBT Displayed in Symptom-Days eReferences. [file jamanetwopen-e2611101-s002.pdf]

Supplemental Online Content

Kleban E, Lee A, Koroma A, et al. Cognitive behavioral therapy for individuals with low literacy and perinatal depression: a randomized clinical trial. *JAMA Netw Open*. 2026;9(5):e2611101. doi:10.1001/jamanetworkopen.2026.11101

eMethods. Sample Size, Randomization, Adapted CBT Intervention Procedure, Data Analysis, and Data Management
eTable 1. Selected Baseline Characteristics of Enrolled Participants by Study Arm and Presence of Primary Outcome Data
eTable 2. Adjusted Efficacy Outcomes
eTable 3. Efficacy Outcomes Stratified by Baseline aPHQ-9 Score
eTable 4. Efficacy Outcomes From a Sensitivity Analysis Imputing a 2-Point Worsening in aPHQ-9 Scores in Place of Missing Outcome Data
eTable 5. Efficacy Outcomes From a Sensitivity Analysis Imputing a 9-Point Improvement in aPHQ-9 Scores in Place of Missing Outcome Data
eTable 6. Distribution of Postintervention Assessment Timing
eTable 7. Efficacy Outcomes Stratified by Timing of Outcome Assessment
eTable 8. Efficacy Outcomes Stratified by Session Attendance
eFigure 1. Adapted PHQ-9 (aPHQ-9) Questionnaire
eFigure 2. Effect of CBT Displayed in Symptom-Days
eReferences.

This supplemental material has been provided by the authors to give readers additional information about their work.

© 2026 Kleban E et al. *JAMA Network Open*.

eMethods. Sample Size, Randomization, Adapted CBT Intervention Procedure, Data Analysis and Data Management

Part A: Sample Size

The Cognitive Behavioral Therapy (CBT) Trial was powered to detect a clinically meaningful difference in depressive symptoms between intervention and control groups. Based on pilot data from Pujehun District, the standard deviation of adjusted Patient Health Questionnaire-9 (aPHQ-9) scores among undernourished pregnant and postpartum women was estimated at four points. At target enrollment of 75 participants per arm (150 total) and assuming 10% loss to follow-up, this sample size provided 80% power at a two-sided $\alpha=0.05$ to detect a 2-point difference between groups, corresponding to a Cohen's d of 0.5. Power was calculated directly for the Wilcoxon-Mann-Whitney test using G*Power (67 per group required), and confirmed by a simulation-based power analysis using the Wilcoxon rank-sum test (59 per group required). The conservative estimate was used and inflated by 10% for anticipated loss to follow-up, yielding 75 per arm.[1,2] The calculation assumed equal allocation, independence of observations, and similar variance across groups. The anticipated retention rate was based on pilot program retention in the same district.

Part B: Randomization

A research staff member not otherwise involved in the trial prepared blocks of 24 small opaque envelopes, each containing one of six symbols, with four of each symbol per block. Three symbols were assigned to each arm. Each block of 24 was placed in a larger, opaque envelope. Participants blindly selected a smaller envelope to determine allocation.

Part C: Adapted CBT Intervention Procedure

Overview of Approach

The adapted CBT intervention was based on core principles of CBT, which emphasize recognizing maladaptive patterns of thinking, understanding how these thoughts shape emotions and actions, and practicing alternative ways of responding. Counselors were trained to guide participants in identifying stressors, clarifying goals, and practicing practical strategies for managing distress. Sessions were collaborative, with women choosing priority problems to work on each week and counselors facilitating participant-led problem solving.

Counseling techniques

Counselors used a structured set of techniques applied flexibly across sessions:

(1) Mood monitoring: Women reflected on changes in their emotional state since the prior visit, encouraging awareness of links between daily events and mood.
(2) Exploring thoughts and feelings: Counselors asked the participant about her feelings about her ongoing challenges and helped her to articulate how these influenced her well-being.
(3) Visualization and goal identification: Women were supported in imagining feasible short-term improvements, which helped reduce a sense of hopelessness and led to the selection of concrete, attainable goals.
(4) Cognitive reframing: Counselors encouraged participants to consider alternative, more helpful perspectives on their difficulties and to contrast current challenges with potential solutions.
(5) Problem-solving: Women practiced generating and evaluating strategies to address identified problems.

Adaptations to CBT for low-literacy and cultural relevance

To ensure the intervention was acceptable in this culture and to women with little or no literacy, several modifications were made:

(1) Elimination of written exercises, replacing them with verbally guided discussions and simple pictorial tools.
(2) Use of locally familiar expressions to describe emotional distress and feelings.
(3) Integration of culturally meaningful metaphors and examples to illustrate CBT concepts.
(4) Attention to social, communal, and spiritual aspects that shape well-being in this context.
(5) Simplified processes for identifying goals, aligned with local norms around planning and decision-making.

Session structure and homework

Sessions followed a general sequence (review of mood and homework, identification of a priority issue, practice of CBT strategies, and planning next steps) but remained flexible to accommodate the participant's needs. Once a problem had been sufficiently addressed, counselors could move on to additional concerns.

Counselors guided women through simple, tailored out-of-session tasks designed to reinforce skills from each session. These could include identifying obstacles, applying CBT strategies to a recent event, or practicing specific actions relative to the week's goal. Homework did not involve reading or writing. Tasks were completed verbally, through pictures, or through real-life practice. For example, a participant who expressed disengagement from daily activities might be

© 2026 Kleban E et al. *JAMA Network Open*.

encouraged to prepare a brief verbal explanation of something she learned or accomplished for discussion at the next session.

Delivery and setting

Counseling was intended to occur at the government clinic where the participant was enrolled. However, home visits were conducted when travel was challenging. All sessions took place in private or semi-private locations, including clinic rooms, outdoor shaded areas, participant homes, or occasionally a vehicle when privacy elsewhere was not possible.

Part D: Data Analysis

In two post-hoc sensitivity analyses, participants without endline aPHQ-9 data had values imputed. In one scenario, assuming poor results among those with missing data, endline aPHQ-9 scores were imputed by adding 2 points to baseline scores. In an alternative, better-case scenario, 9 points were subtracted from baseline scores to impute endline scores. We conducted these worse- and better-case imputation scenarios using fixed offsets to baseline scores, as justified in prior methodological work.[3,4] Participants were analyzed according to their allocated group irrespective of treatment received or adherence to it.

Pre-specified secondary analyses adjusted for maternal age, education level, and baseline aPHQ-9 score were done for continuous outcomes using ordinal logistic regression and for binary outcomes using logistic regression. The proportional odds assumption was deemed met after plotting empirical logits against cut-point values for each treatment group and identifying parallelism (Harrell). Using ordinal logistic regression, we assessed for heterogeneity of effect in endline aPHQ-9 scores by plotting ORs and 95% CIs by subgroup as well as estimating a p value for the interaction term between study group and each subgroup. In all cases, OR>1 indicates greater odds for a lower aPHQ-9 score. Pre-specified subgroups were maternal age, education level, randomized nutrition supplement group, and baseline aPHQ-9; post-hoc subgroups were first pregnancy vs. other, father in home, antenatal vs. postnatal, and gestational age at CBT enrollment among participants enrolled antenatally.

Review of timing of outcome assessment relative to week of randomization revealed that control group participants were more likely to have had their outcome assessed earlier, relative to CBT participants. A post-hoc analysis comparing effect of CBT vs. control stratified by timing of outcome assessment was thus performed.

Inter-assessor reliability of aPHQ-9 assessment was analyzed using a two-way random-effects model and reported as ICC (95% CI). All statistical analyses were performed using R version 4.5.0.

Part E: Data Management

Data were collected on paper forms at each clinic and securely stored in the Project Peanut Butter office. Research staff double-entered all data into password-protected Microsoft Access databases and verified for accuracy. All paper copies were scanned as a backup. Data handling followed local and institutional guidelines for confidentiality and security.

© 2026 Kleban E et al. *JAMA Network Open*.

eTable 1. Selected Baseline Characteristics of Enrolled Participants by Study Arm and Presence of Primary Outcome Data[a]

Characteristic	Known primary outcome		Unknown primary outcome	
	**CBT (n = 75)**	**Control (n = 65)**	**CBT (n = 4)**	**Control (n = 9)**
Age, median (IQR), y[b]	19 (18, 22)	19 (18, 23)	19 (18, 27)	21 (18, 23)
MUAC at enrollment, median (IQR), cm	22.7 (22.0, 23.0)	22.4 (21.4, 22.9)	22.0 (20.9, 22.2)	22.0 (20.4, 22.6)
Weeks in supplementary feeding trial before enrollment in this trial, median (IQR)[c]	4 (0, 10)	4 (0, 10)	11 (4, 31)	0 (0, 4)
0	36 (48.0)	28 (43.1)	1 (25.0)	5 (55.6)
≤ 4	14 (18.7)	9 (13.8)	0 (0.0)	2 (22.2)
> 4	25 (33.3)	28 (43.1)	3 (75.0)	2 (22.2)
Pregnancy status at enrollment				
Antenatal	68 (90.6)	57 (87.7)	2 (50.0)	7 (77.8)
Gestational age at enrollment, median (IQR), wk[d]	22.5 (19.0, 28.2)	20.9 (17.2, 26.1)	20.8 (8.0, 33.6)	24.4 (21.0, 27.3)
Postnatal	7 (9.3)	8 (12.3)	2 (50.0)	2 (22.2)
Weeks since delivery at enrollment	13.2 (12.3, 13.9)	6.6 (6.2, 10.0)	26.7 (13.7, 39.6)	5.7 (5.4, 6.0)
Mother's education level				
None	13 (17.3)	18 (27.7)	1 (25.0)	3 (33.3)
Primary	6 (8.0)	10 (15.4)	1 (25.0)	1 (11.1)
Secondary or greater	56 (74.7)	37 (56.9)	2 (50.0)	5 (55.6)
Currently in school[e]	30 (40.0)	16 (24.6)	2 (50.0)	0 (0.0)
History of miscarriage or stillbirth	2 (2.7)	6 (9.2)	0 (0.0)	1 (11.1)
Number of adults in household, median (IQR)	4 (3, 5)	4 (3, 5)	2 (2, 4)	4 (2, 6)
Two or more children live in household	55 (73.3)	52 (80.0)	3 (75.0)	6 (66.7)
Father lives in household	51 (68.0)	42 (64.6)	3 (75.0)	5 (55.6)
Household roof made of thatch	8 (10.7)	8 (12.3)	2 (50.0)	0 (0.0)
Pit latrine used for stool disposal	63 (84.0)	53 (81.5)	4 (100.0)	8 (88.9)
Household has electricity	7 (9.3)	11 (16.9)	0 (0.0)	0 (0.0)
Household Food Insecurity Access Scale score (past 4 weeks)				
Food secure	2 (2.7)	7 (10.8)	0 (0.0)	1 (11.1)
Mildly food insecure	0 (0.0)	1 (1.5)	0 (0.0)	0 (0.0)
Moderately food insecure	31 (41.3)	18 (27.7)	1 (25.0)	3 (33.3)
Severely food insecure	42 (56.0)	39 (60.0)	3 (75.0)	5 (55.6)
aPHQ-9 score at time of enrollment, median (IQR)	11 (10, 12)	11 (9, 12)	12 (10, 13)	10 (9, 11)
Score of 9	10 (13.3)	17 (26.2)	1 (25.0)	4 (44.4)
Score 10-12	50 (66.7)	39 (60.0)	2 (50.0)	5 (55.6)
Score ≥ 13	15 (20.0)	9 (13.8)	1 (25.0)	0 (0.0)

Abbreviations: aPHQ-9, 9-item adapted Patient Health Questionnaire
[a] Values are No. (%) unless otherwise indicated
[b] n = 2 missing from CBT and n = 1 missing from control, all with known primary outcomes
[c] CBT trial participants are a subset of a larger supplementary feeding study, details of which can be found in Supplement 1.
[d] n = 2 missing from control due to miscarriage prior to gestational age estimation being possible, one with known primary outcomes
[e] n = 1 missing from control, with unknown primary outcome

© 2026 Kleban E et al. *JAMA Network Open*.

eTable 2. Adjusted Efficacy Outcomes[a]

Outcome	CBT (n = 79)	Control (n = 74)	Odds Ratio[b] (95% CI)	*P* value[c]
Endline aPHQ-9				
No. (%) of participants[d]	73 (92.4)	64 (86.4)		
Median (IQR)	2 (1, 4)	7 (3, 9)	11.09 (5.43, 22.62)	<0.001
Secondary outcomes				
No. (%) of participants	73 (92.4)	64 (86.4)		
Change in aPHQ-9, median (IQR)	-9 (-11, -7)	-4 (-7, -2)	11.36 (5.52, 23.37)	<0.001
Reduction in aPHQ-9 score from baseline to endline, No. (%)				
≤ 3 points	3 (4.0)	29 (44.6)	0.05 (0.01, 0.15)	<0.001
> 3 points	72 (96.0)	36 (55.4)	20.80 (6.58, 93.08)	<0.001
> 5 points	66 (88.0)	26 (40.0)	15.32 (6.17, 42.01)	<0.001
> 9 points	30 (40.0)	4 (6.2)	19.28 (5.26, 102.69)	<0.001
> 50%	68 (90.7)	29 (44.6)	18.67 (7.10, 57.00)	<0.001
Endline aPHQ-9 < 5	59 (78.7)	22 (33.8)	11.47 (4.89, 29.28)	<0.001
Postnatal outcomes among antenatal enrollees				
No. (%) of antenatal enrollees	70 (88.6)	64 (86.5)		
No. (%) at 1.5 mo postnatal	60 (85.7)	54 (84.4)		
aPHQ-9 score, Median (IQR)	2 (0, 3)	3 (0, 6)	2.87 (1.43, 5.74)	0.003
aPHQ-9 < 5, No. (%)	50 (83.3)	30 (55.6)	4.40 (1.84, 11.29)	0.001
No. (%) at 3 mo postnatal	59 (84.3)	50 (78.1)		
aPHQ-9 score, Median (IQR)	2 (0, 4)	4 (1, 7)	2.86 (1.39, 5.90)	0.004
aPHQ-9 < 5, No. (%)	49 (83.1)	27 (54.0)	4.76 (1.92, 12.67)	0.001
No. (%) at 6 mo postnatal	53 (75.7)	45 (70.3)		
aPHQ-9 score, Median (IQR)	2 (0, 4)	3 (0, 6)	1.97 (0.95, 4.06)	0.07
aPHQ-9 < 5, No. (%)	40 (75.5)	28 (62.2)	2.06 (0.82, 5.31)	0.13
No. (%) at 9 mo postnatal	50 (71.4)	45 (70.3)		
aPHQ-9 score, Median (IQR)	0 (0, 3)	2 (0, 5)	3.19 (1.44, 7.07)	0.004
aPHQ-9 < 5, No. (%)	44 (88.0)	33 (73.3)	3.37 (1.10, 11.67)	0.041

Abbreviations: aPHQ-9, 9-item adapted Patient Health Questionnaire
[a] Values are median (IQR) unless otherwise indicated. All analyses are adjusted for maternal age, education level, and baseline aPHQ-9 score. Two CBT participants and one control participant did not provide their age and were excluded from analyses.
[b] For continuous outcomes, this represents an odds ratio for a lower aPHQ-9 score. Values > 1 indicate greater odds for lower scores for CBT compared with control. For binary outcomes, this represents an odds ratio, estimated using logistic regression. Values > 1 indicate greater odds for CBT relative to control.
[c] The Wald test was used to estimate p values for all outcomes.
[d] Maternal age missing for CBT (n = 2) and control (n = 1) groups leading to exclusion from adjusted analyses.

© 2026 Kleban E et al. *JAMA Network Open.*

eTable 3. Efficacy Outcomes Stratified by Baseline aPHQ-9 Score[a]

Outcome	CBT (n = 79)	Control (n = 74)	Comparison[b] (95% CI)	*P* value[c]
Has endline aPHQ-9 score	75 (94.9)	65 (87.8)		
Baseline aPHQ-9 ≥ 10	65 (86.7)	48 (73.8)		
Endline aPHQ-9, median (IQR)	3 (1, 4)	8 (4, 10)	-5 (-6, -3)	<0.001
Endline aPHQ-9 < 5	50 (76.9)	12 (25.0)	9.75 (3.87, 26.34)	<0.001
Baseline aPHQ-9 ≥ 11	50 (63.3)	36 (48.6)		
Endline aPHQ-9, median (IQR)	3 (1, 4)	8 (4, 10)	-5 (-6, -3)	<0.001
Endline aPHQ-9 < 5	40 (80.0)	9 (25.0)	11.56 (3.89, 38.15)	<0.001
Baseline aPHQ-9 ≥ 12	34 (43.0)	23 (31.1)		
Endline aPHQ-9, median (IQR)	3 (2, 4)	9 (5, 10)	-5 (-7, -3)	<0.001
Endline aPHQ-9 < 5	26 (76.4)	5 (21.7)	11.08 (2.86, 51.70)	<0.001
Baseline aPHQ-9 ≥ 13	15 (19.0)	9 (12.2)		
Endline aPHQ-9, median (IQR)	3 (2, 4)	9 (6, 10)	-6 (-8, -2)	0.010
Endline aPHQ-9 < 5	12 (80.0)	2 (22.2)	12.10 (1.40, 181.13)	0.010

Abbreviations: aPHQ-9, 9-item adapted Patient Health Questionnaire
[a] Values are No. (%) unless otherwise indicated.
[b] For continuous outcomes, this represents the Hodges-Lehmann median of differences, with 95% confidence intervals calculated using normal approximation with continuity correction. Values < 0 indicate lower scores for CBT compared with control. For binary outcomes, this represents an odds ratio, estimated using logistic regression. Values > 1 indicate greater odds for CBT relative to control.
[c] For continuous outcomes, p values were estimated using the Wilcoxon rank-sum test. For binary outcomes, the Wald test was used to estimate p values.

© 2026 Kleban E et al. *JAMA Network Open*.

eTable 4. Efficacy Outcomes from a Sensitivity Analysis Imputing a 2-point Worsening in aPHQ-9 Scores in Place of Missing Outcome Data[a]

Outcome	CBT (n = 79)	Control (n = 74)	Comparison[b] (95% CI)	*P* value[c]
Endline aPHQ-9	3 (1, 4)	8 (3, 10)	-4 (-5, -3)	<0.001
Change in aPHQ-9, endline - baseline	-9 (-10, -7)	-3 (-7, -1)	-4 (-6, -4)	<0.001
Reduction in aPHQ-9 score from baseline to endline, No. (%)				
≤ 3 points	7 (8.9)	38 (51.4)	0.09 (0.03, 0.22)	<0.001
> 3 points	72 (91.1)	36 (48.6)	10.86 (4.65, 28.75)	<0.001
> 5 points	66 (83.5)	26 (35.1)	9.37 (4.48, 20.75)	<0.001
> 9 points	30 (38.0)	4 (5.4)	10.71 (3.92, 37.80)	<0.001
> 50%	68 (86.1)	29 (39.2)	9.59 (4.49, 21.98)	<0.001
Endline aPHQ-9 < 5, No. (%)	59 (74.7)	22 (29.7)	6.97 (3.48, 14.50)	<0.001

Abbreviations: aPHQ-9, 9-item adapted Patient Health Questionnaire
[a] Values are median (IQR) unless otherwise indicated.
[b] For continuous outcomes, this represents the Hodges-Lehmann median of differences, with 95% confidence intervals calculated using normal approximation with continuity correction. Values < 0 indicate lower scores for CBT compared with control. For binary outcomes, this represents an odds ratio, estimated using logistic regression. Values > 1 indicate greater odds for CBT relative to control.
[c] For continuous outcomes, p values were estimated using the Wilcoxon rank-sum test. For binary outcomes, the Wald test was used to estimate p values.

© 2026 Kleban E et al. *JAMA Network Open*.

eTable 5. Efficacy Outcomes from a Sensitivity Analysis Imputing a 9-point Improvement in aPHQ-9 Scores in Place of Missing Outcome Data[a]

**Outcome**	**CBT (n = 79)**	**Control (n = 74)**	**Comparison[b] (95% CI)**	***P* value[c]**
Endline aPHQ-9	2 (1, 4)	5 (2, 9)	-3 (-4, -2)	<0.001
Change in aPHQ-9, endline - baseline	-9 (-10, -7)	-5 (-8, -2)	-4 (-5, -3)	<0.001
Reduction in aPHQ-9 score from baseline to endline, No. (%)				
≤ 3 points	3 (3.8)	29 (39.2)	0.06 (0.01, 0.18)	<0.001
> 3 points	76 (96.2)	45 (60.8)	16.33 (5.41, 70.93)	<0.001
> 5 points	70 (88.6)	35 (47.3)	8.67 (3.92, 20.95)	<0.001
> 9 points	30 (38.0)	4 (5.4)	10.71 (3.92, 37.80)	<0.001
> 50%	72 (91.1)	38 (51.4)	9.74 (4.17, 25.80)	<0.001
Endline aPHQ-9 < 5, No. (%)	63 (79.7)	31 (41.9)	5.46 (2.71, 11.45)	<0.001

Abbreviations: aPHQ-9, 9-item adapted Patient Health Questionnaire
[a] Values are median (IQR) unless otherwise indicated.
[b] For continuous outcomes, this represents the Hodges-Lehmann median of differences, with 95% confidence intervals calculated using normal approximation with continuity correction. Values < 0 indicate lower scores for CBT compared with control. For binary outcomes, this represents an odds ratio, estimated using logistic regression. Values > 1 indicate greater odds for CBT relative to control.
[c] For continuous outcomes, p values were estimated using the Wilcoxon rank-sum test. For binary outcomes, the Wald test was used to estimate p values.

© 2026 Kleban E et al. *JAMA Network Open.*

eTable 6. Distribution of Postintervention Assessment Timing

**Measure**	**CBT (n=75)**	**Control (n=65)**	**Total (n=140)**
Week post-randomization of endline assessment			
7	1 (1.3)	9 (13.8)	10 (7.1)
8	30 (40.0)	39 (60.0)	69 (49.3)
9-10	18 (24.0)	10 (15.4)	28 (20.0)
11-12	18 (24.0)	4 (6.2)	22 (15.7)
13-14	7 (9.3)	1 (1.5)	8 (5.7)
15-16	1 (1.3)	2 (3.1)	3 (2.1)
Median (IQR) of weeks post-randomization	10 (8, 12)	8 (8, 9)	8 (8, 10)

Abbreviations: CBT, cognitive behavioral therapy; IQR, interquartile range.
Values are No. (%) unless otherwise indicated. Endline was defined as the aPHQ-9 score collected within a pre-specified 7–16 week window post-randomization, prioritized by proximity to the target 8-week assessment.

© 2026 Kleban E et al. *JAMA Network Open*.

eTable 7. Efficacy Outcomes Stratified by Timing of Outcome Assessment[a]

Outcome	CBT (n = 79)	Control (n = 74)	Comparison[b] (95% CI)
Has endline aPHQ-9 score	75 (94.9)	65 (87.8)	
7 weeks after randomization	1 (1.3)	9 (13.8)	
Endline aPHQ-9, median (IQR)	3 (3, 3)	5 (5, 8)	-2 (-7, 1)
Endline aPHQ-9 < 5	1 (100.0)	2 (22.2)	NA
8 weeks after randomization	30 (40.0)	39 (60.0)	
Endline aPHQ-9, median (IQR)	2 (1, 5)	7 (3, 10)	-4 (-6, -2)
Endline aPHQ-9 < 5	22 (73.3)	14 (35.9)	4.79 (1.56, 16.05)
9-10 weeks after randomization	18 (24.0)	10 (15.4)	
Endline aPHQ-9, median (IQR)	2 (0, 3)	7 (4, 10)	-5 (-8, -2)
Endline aPHQ-9 < 5	17 (94.4)	3 (30.0)	32.30 (2.80,1858.96)
11-12 weeks after randomization	18 (24.0)	4 (6.2)	
Endline aPHQ-9, median (IQR)	3 (0, 5)	5 (2, 9)	-2 (-8, 2)
Endline aPHQ-9 < 5	13 (72.2)	2 (50.0)	2.48 (0.14–43.45)
13-14 weeks after randomization	7 (9.3)	1 (1.5)	
Endline aPHQ-9, median (IQR)	4 (2, 6)	5 (5, 5)	-1 (-5, 3)
Endline aPHQ-9 < 5	5 (71.4)	0 (0.0)	NA
15-16 weeks after randomization	1 (1.3)	2 (3.1)	
Endline aPHQ-9, median (IQR)	2 (2, 2)	4 (4, 4)	-2 (-3, -1)
Endline aPHQ-9 < 5	1 (100.0)	1 (50.0)	NA

Abbreviations: aPHQ-9, 9-item adapted Patient Health Questionnaire

[a] Values are No. (%) unless otherwise indicated.

[b] For continuous outcomes, this represents the Hodges-Lehmann median of differences, with 95% confidence intervals calculated using normal approximation with continuity correction. Values < 0 indicate lower scores for CBT compared with control. For binary outcomes, this represents an odds ratio, estimated using logistic regression. Values > 1 indicate greater odds for CBT relative to control. NA indicates not able to be estimated.

[c] For continuous outcomes, p values were estimated using the Wilcoxon rank-sum test. For binary outcomes, the Wald test was used to estimate p values.

© 2026 Kleban E et al. *JAMA Network Open*.

eTable 8. Efficacy Outcomes Stratified by Session Attendance[a]

Outcome	CBT (n = 79)
Has endline aPHQ-9 score	75 (94.9)
Attended 1 session	4 (5.1)
Endline aPHQ-9, median (IQR)	7 (6, 7)
Endline aPHQ-9 < 5	0 (0.0)
Attended 2 sessions	2 (2.7)
Endline aPHQ-9, median (IQR)	4 (3, 4)
Endline aPHQ-9 < 5	2 (100.0)
Attended 3 sessions	1 (1.3)
Endline aPHQ-9, median (IQR)	3 (3, 3)
Endline aPHQ-9 < 5	1 (100.0)
Attended 5 sessions	2 (2.7)
Endline aPHQ-9, median (IQR)	4 (4, 5)
Endline aPHQ-9 < 5	1 (50.0)
Attended 6 sessions	66 (88.0)
Endline aPHQ-9, median (IQR)	2 (0, 4)
Endline aPHQ-9 < 5	55 (83.3)

Abbreviations: aPHQ-9, 9-item adapted Patient Health Questionnaire
[a] Values are No. (%) unless otherwise indicated.

© 2026 Kleban E et al. *JAMA Network Open*.

eFigure 1. Adapted PHQ-9 (aPHQ-9) Questionnaire

COGENT Study – PHQ-9 Questionnaire **STUDY ID**: _____________

Date: ____/____/____ Mother's Name: ______________________ Clinic: _______________________

Over the last 2 weeks, how often have you been bothered by any of the following problems?

Insay di las 2 wik dɛm, ɔmɔs tɛm ɛni wan pan dɛn prɔblɛm dɛm ya dɔn mɔna yu?

Q		Not at all<br><br>I nɔ de apin at ɔl<br>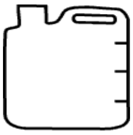	Once or twice<br><br>I de apin bɔt na lɛkɛ wan or tu tem insay di las/pas tu wik<br>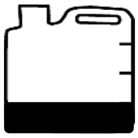	More than a week<br><br>I don de apin lɛkɛ tri to fayv tem inay di pas wan wik<br>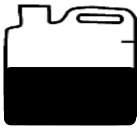	Nearly every day<br><br>I don de apin naw Ɔlmos evri de insay di wik<br>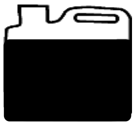	Comments
1	Little interest or pleasure in doing things<br><br>Yu no de enjoy som pan di tin den we yu bin de enjoy fo du trade	0	1	2	3	
2	Feeling down, depressed, or hopeless<br><br>Yu nɔ gladi, yu at pwɛl en yu nɔ abop se enitin go bɛtɛ ɔ chanj atɔl	0	1	2	3	
3	Trouble falling or staying asleep, or sleeping too much<br><br>I mɔna fɔ mɛk slip tek yu ɔ yu nɔ de te pa slip/slip fɔ lɔŋ ɔ yu de slip slip tumɔs.	0	1	2	3	
4	Feeling tired or having little energy<br><br>De fil taya ɔ nɔ get bɛtɛ ɛnaji	0	1	2	3	
5	Poor appetite<br><br>Nɔ de fil fɔ it bɛtɛ	0	1	2	3	

© 2026 Kleban E et al. *JAMA Network Open.*

6	Feeling bad about yourself — or that you are a failure or have let yourself or your family down<br><br>De fil bad bɔt yu sɛf – ɔ lɛk yu dɔn fel na layf ɔ lɛk yu dɔn fɔm pa yusɛf ɔ yu famili.	0	1	2	3	
7	Trouble concentrating on things, such as doing household chores<br><br>I mɔna fɔ mek yu put yu maynd/at pa sɔmtin, lɛk fɔ do os wok dɛm	0	1	2	3	
8	Moving or speaking so slowly that other people could have noticed? Or the opposite — being so restless that you have been moving around a lot more than usual<br><br>De du tin dɛm ɔ tɔk tu slo dat ɔda pipul dɛm kin ebul fɔ tek notis? Ɔ de mago mago ɔ nɔ ebul fɔ stɛdi wan ples ɛn de pas pas bɔku pas aw yu kin de.	0	1	2	3	
9	Thoughts that you would be better off dead or of hurting yourself in some way<br><br>De tink se I go bɛtɛ fɔ yu if yu jis day ɔ du bad tin to yu sɛf sɔm kayn we.	0	1	2	3	

TOTAL SCORE: ______________

© 2026 Kleban E et al. *JAMA Network Open*.

eFigure 2. Efficacy of CBT expressed as symptom-days

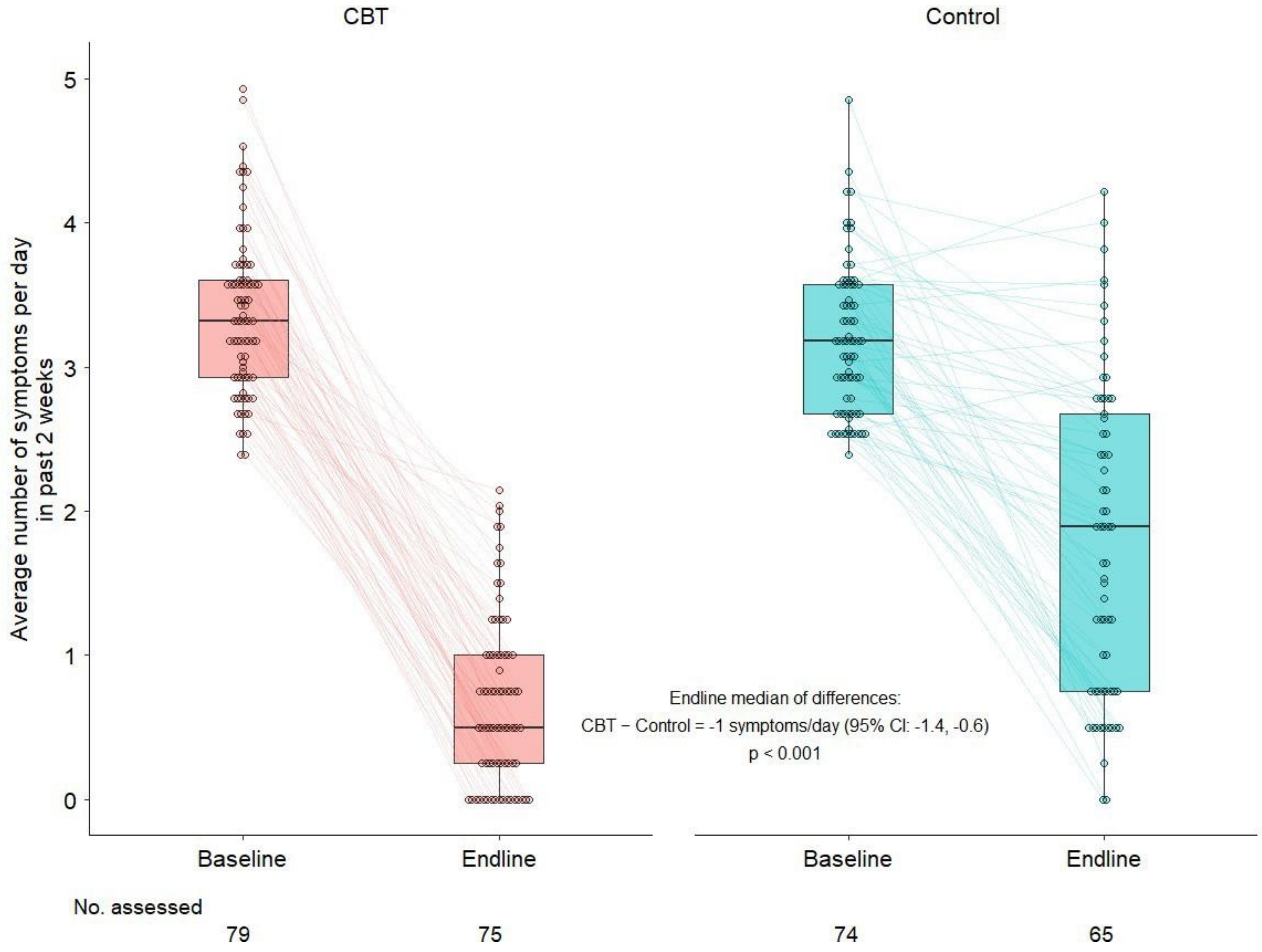

Item-level aPHQ-9 scores were back-converted to symptom-days in the following manner: 0 = 0 days, 1 = 3.5 days (midpoint of 1-6), 2 = 9 days (midpoint of 7-11), and 3 = 13 days (midpoint 12-14). The central horizontal line of the box plot represents the median of symptom-days, while the upper and lower edges of the box represent the IQR and the error bars represent 95% of the data range. The dots represent individual symptom-day values which are jittered horizontally in proportion to the number of data points at each score. Lines connect baseline and endline scores, when both are present. The Hodges-Lehmann median of differences is presented alongside a continuity-corrected 95% CI and p value estimated using the Wilcoxon rank-sum test.

© 2026 Kleban E et al. *JAMA Network Open*.

eReferences

1. Waqas A, Zafar SW, Akhtar P, Naveed S, Rahman A. Optimizing cognitive and behavioral approaches for perinatal depression: A systematic review and meta-regression analysis. *Glob Ment Health (Camb)*. 2023;10:e22. doi:10.1017/gmh.2023.8

2. Pettman D, O'Mahen H, Blomberg O, Svanberg AS, von Essen L, Woodford J. Effectiveness of cognitive behavioural therapy-based interventions for maternal perinatal depression: a systematic review and meta-analysis. *BMC Psychiatry*. 2023;23(1):208. doi:10.1186/s12888-023-04547-9

3. Cro S, Morris TP, Kenward MG, Carpenter JR. Sensitivity analysis for clinical trials with missing continuous outcome data using controlled multiple imputation: A practical guide. *Statistics in Medicine*. 2020;39(21):2815-2842. doi:10.1002/sim.8569

4. White IR, Carpenter J, Horton NJ. A mean score method for sensitivity analysis to departures from the missing at random assumption in randomised trials. *Stat Sin*. 2018;28(4):1985-2003. doi:10.5705/ss.202016.0308

© 2026 Kleban E et al. *JAMA Network Open*.
