# Supplementary material for: Cognitive Behavioral Therapy for Individuals With Low Literacy and Perinatal Depression: A Randomized Clinical Trial
Source: JAMA Netw Open. 2026 May 7;9(5):e2611101. doi: 10.1001/jamanetworkopen.2026.11101 (PMC13153995; doi:10.1001/jamanetworkopen.2026.11101)
Supplement: Supplement 3. — Data Sharing Statement [file jamanetwopen-e2611101-s003.pdf]

Data Sharing Statement

Kleban. Cognitive Behavioral Therapy for Individuals With Low Literacy and Perinatal Depression: A Randomized Clinical Trial. *JAMA Netw Open*. Published online May 7, 2026. doi:10.1001/jamanetworkopen.2026.11101

Data

Additional Information: COGENT - Improving COgnition and GEstational duration with targeted NuTrition, https://clinicaltrials.gov/study/NCT05949190 , National Clinical Trial (NCT) Identified Number: NCT05949190
Data available: Yes
Data types: Deidentified participant data, Data dictionary
How to access data: This data will be made available upon request to Mark J Manary (manarymj@wustl.edu).
When available: With publication

Supporting Documents

Document types: Statistical/analytic code
How to access documents: The supporting documents will be available as supplementary material with the publication
When available: With publication

Additional Information

Who can access the data: Any investigator who can submits a request.
Types of analyses: Standard comparisons of groups.
Mechanisms of data availability: Upon request from the Washington University repository
Any additional restrictions: None
